# hUC-MSC-mediated recovery of subacute spinal cord injury through enhancing the pivotal subunits β3 and γ2 of the GABA_A_ receptor

**DOI:** 10.7150/thno.72015

**Published:** 2022-03-28

**Authors:** Tingting Cao, Huan Chen, Weiping Huang, Sisi Xu, Peilin Liu, Weiwei Zou, Mao Pang, Ying Xu, Xiaochun Bai, Bin Liu, Limin Rong, Zhong-Kai Cui, Mangmang Li

**Affiliations:** 1 Department of Cell Biology, School of Basic Medical Sciences, Southern Medical University, Guangzhou, 510515, China.; 2 Department of Spine Surgery, The Third Affiliated Hospital of Sun Yat-sen University, Guangzhou, 510515, China.; 3 Guangdong Provincial Center for Engineering and Technology Research of Minimally Invasive Spine Surgery, 510630, China.; 4 Guangdong Provincial Center for Quality Control of Minimally Invasive Spine Surgery, 510630, China.

**Keywords:** spinal cord injury, human umbilical cord mesenchymal stem cells, transcriptome, γ-aminobutyric acid type A (GABA_A_) receptor, K^+^-Cl^⁻^ cotransporter 2

## Abstract

**Rationale:** Spinal cord injury (SCI) remains an incurable neurological disorder leading to permanent and profound neurologic deficits and disabilities. Human umbilical cord-derived mesenchymal stem cells (hUC-MSCs) are particularly appealing in SCI treatment to curtail damage, restore homeostasis and possible neural relay. However, the detailed mechanisms underlying hUC-MSC-mediated functional recovery of SCI have not been fully elucidated. The purpose of our current study is to identify novel therapeutic targets and depict the molecular mechanisms underlying the hUC-MSC-mediated recovery of subacute SCI.

**Methods:** Adult female rats suffering from subacute incomplete thoracic SCI were treated with intrathecal transplantation of hUC-MSCs. The beneficial effects of hUC-MSCs on SCI repair were evaluated by a series of behavioral analyses, motor evoked potentials (MEPs) recording of hindlimb and immunohistochemistry. We carried out extensive transcriptome comparative analyses of spinal cord tissues at the lesion site from the subacute phase of SCI (sub-SCI) either treated without (+PBS) or with hUC-MSCs (+MSC) at 0 (sub-SCI), 1, 2, and 4 weeks post-transplantation (wpt), as well as normal spinal cord segments of intact/sham rats (Intact). Adeno-associated virus (AAV)-mediated neuron-specific expression system was employed to functionally screen specific γ-aminobutyric acid type A receptor (GABA_A_R) subunits promoting the functional recovery of SCI *in vivo*. The mature cortical axon scrape assay and transplantation of genetically modified MSCs with either overexpression or knockdown of brain-derived neurotrophic factor (BDNF) were employed to demonstrate that hUC-MSCs ameliorated the reduction of GABA_A_R subunits in the injured spinal cord *via* BDNF secretion *in vitro* and *in vivo*, respectively.

**Results:** Comparative transcriptome analysis revealed the GABAergic synapse pathway is significantly enriched as a main target of hUC-MSC-activated genes in the injured spinal cord. Functional screening of the primary GABA_A_R subunits uncovered that Gabrb3 and Garbg2 harbored the motor and electrophysiological recovery-promoting competence. Moreover, targeting either of the two pivotal subunits β3 or γ2 in combination with/without the K^+^/Cl^-^ cotransporter 2 (KCC2) reinforced the therapeutic effects. Mechanistically, BDNF secreted by hUC-MSCs contributed to the upregulation of GABA_A_R subunits (β3 & γ2) and KCC2 in the injured neurons.

**Conclusions:** Our study identifies a novel mode for hUC-MSC-mediated locomotor recovery of SCI through synergistic upregulation of GABA_A_R β3 and γ2 along with KCC2 by BDNF secretion, indicating the significance of restoring the excitation/inhibition balance in the injured neurons for the reestablishment of neuronal circuits. This study also provides a potential combinatorial approach by targeting the pivotal subunit β3 or γ2 and KCC2, opening up possibilities for efficacious drug design.

## Introduction

The traumatic spinal cord injury (SCI) remains an incurable neurological disorder in clinics due to its complex pathophysiology. Poor understanding of the pathophysiologic and molecular mechanisms of SCI hampers the development of effective therapeutic strategies. To conquer the challenge, several recent studies have performed RNA-seq on injured spinal cords in animal models and analyzed the stage-associated pathological events at transcriptional levels 1-4. In particular, the synaptic transmission-related genes, including ion channels, neurotransmitter receptors, and axon/dendrite-related genes, are decreased right after SCI and remain low, indicating a permanent impairment of neurotransmission 1. Therefore, the ultimate objective of SCI research is to develop strategies to improve or restore the disrupted neural circuits. Recently, cell therapies, especially mesenchymal stem cell (MSC) transplantation, have garnered much attention due to various benefits, including immunomodulation, anti-inflammation, anti-apoptosis, axon regeneration, and remyelination 5, 6. Although the paracrine capacity of MSCs most likely contributes to their therapeutic effects, the targeting cell types of MSCs and detailed mechanisms have not been elucidated.

We previously characterized the dose effect of hUC-MSC transplantation on transcriptional regulation of 40 genes encoding main subunits of different ion channels and receptors in the injured spinal cord 7. Only the effective dosages could significantly reverse the decrease in all γ-aminobutyric acid (GABA) type A receptor (GABA_A_R) subunits caused by SCI. Our data demonstrated GABA_A_Rs were potentially therapeutic targets of hUC-MSCs in the injured spinal cord. GABA as well as glycine are the main inhibitory neurotransmitter of CNS, and primarily mediate fast inhibition. GABAergic transmission is mediated by GABA receptors which can be categorized into two major types based on their functions, ionotropic GABA_A_Rs and GABA_C_Rs, as well as metabotropic GABA_B_Rs 8, 9. GABA_A_Rs are heteropentameric ligand-gated chloride (Cl⁻)-permeable channels with different combinations of subunits encoded by at least 19 genes (α1-6, β1-3, γ1-3, δ, ε, θ, π, ρ1-3 and τ) in mammals 10. The binding of GABA to GABA_A_Rs opens the Cl⁻ channels. However, depolarizing or hyperpolarizing effect of GABA is also dependent on the intracellular Cl⁻ concentration ([Cl⁻]_i_), which is regulated by cation-chloride cotransporters (CCCs), including the K⁺-Cl⁻ cotransporter 2 (KCC2) and Na^+^-K^+^-2Cl⁻ cotransporter 1 (NKCC1) in CNS. Therefore, both the activities of GABA_A_Rs and CCCs are critical for the GABAergic inhibition. In healthy mature neurons, intracellular Cl⁻ is usually maintained at a low concentration because of the high expression of Cl⁻ extruder KCC2, enabling inhibition of hyperpolarizing GABAergic signaling 11. Recent advancements have indicated that KCC2 activity is involved in a number of diseases including epilepsy, schizophrenia, as well as neuropathic pain and impaired locomotor function following SCI 12. Functional coupling of GABA_A_Rs and KCC2 are able to shape GABAergic signaling and neuronal connectivity. Nonetheless, little is known about the effect of hUC-MSC transplantation on the regulation of GABA_A_Rs and KCC2 after SCI.

In order to identify novel therapeutic targets and depict the molecular mechanisms underlying the hUC-MSC-mediated recovery of subacute SCI, we carried out a time-course transcriptome analysis of spinal cord tissues at the lesion site from a subacute phase of SCI either treated without or with hUC-MSCs to screen the potential direct and/or indirect therapeutic targets of hUC-MSCs. Surprisingly, our results have identified that the restoration of GABA_A_R subunits β3 or γ2 in the injured spinal neurons leads to the functional recovery to an extent identical to KCC2. Furthermore, combinational treatment with either of these two subunits and KCC2 reinforced the therapeutic effects, opening up possibilities for efficacious drug design for SCI repair.

## Results

### hUC-MSC-activated and -repressed genes are functionally associated with neural regeneration and elimination of astrocytic scarring and inflammation

**Figure 1:**
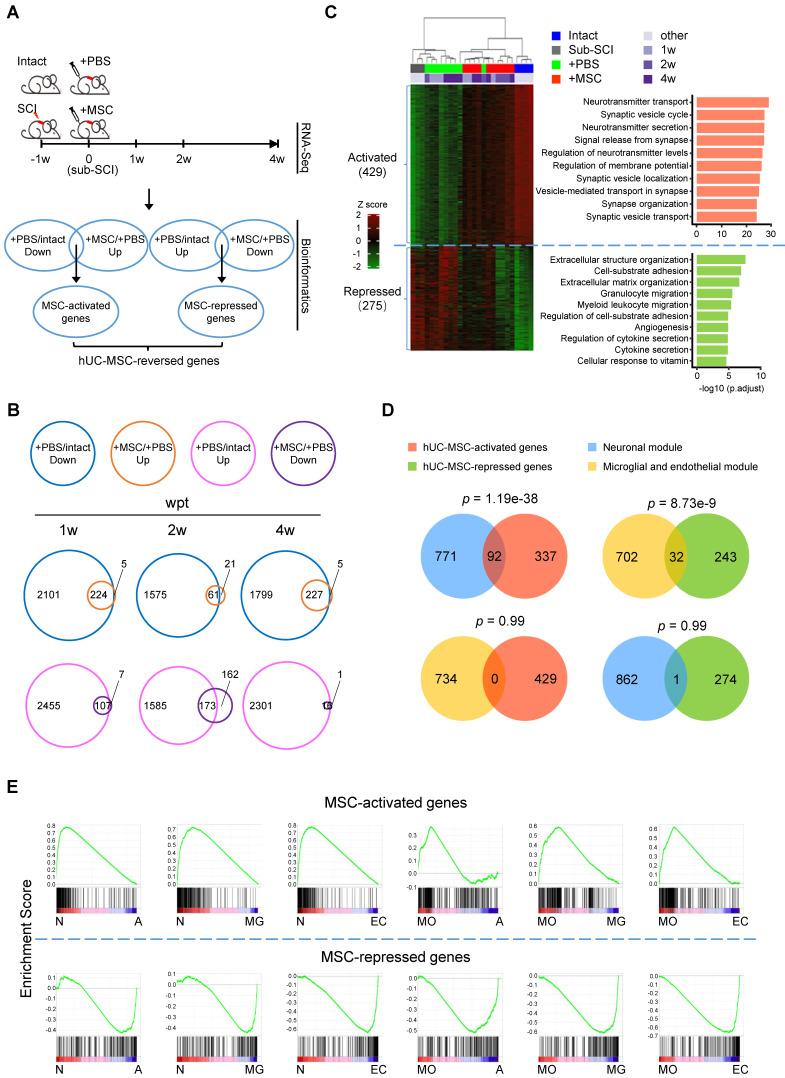
**Comparative transcriptome analysis identifies hUC-MSC-reversed genes in the injured spinal cord.** (A) The experimental design for spinal cord transcriptome screen for hUC-MSC-reversed transcripts in rat models with subacute SCI (sub-SCI) treated with or without hUC-MSCs. The incomplete thoracic SCI was performed one week (-1w) before the hUC-MSC transplantation (0w), and 5-mm spinal cords centered at the lesion site from indicated groups were sampled for RNA-sequencing at 1w, 2w, and 4w post-transplantation. Intact, normal rats; sub-SCI, rats with subacute SCI; +PBS, rats with sub-SCI intrathecally injected with phosphate-buffered saline (PBS); +MSC, rats with sub-SCI transplanted with hUC-MSCs. (B) Venn diagrams showing the number of hUC-MSC-reversed transcripts at 1, 2, and 4 wpt, respectively. The overlap between genes downregulated by SCI (blue circle) and genes upregulated by MSC transplantation (orange circle) represents the hUC-MSC-activated genes (top). The overlap between genes upregulated by SCI (carmine circle) and genes downregulated by the MSC transplantation (purple circle) represents the hUC-MSC-reversed genes (bottom). (C) Hierarchical clustering based on the 704 hUC-MSC-reversed DEGs among all 26 samples. Genes are rank-ordered by the expression variances shown by z-score (represented by a color bar on the left side). (Right) The top ten biological processes for the hUC-MSC-activated (red) and -repressed (green) genes ranked by adjusted *p* value. (D) Venn diagram showing the overlap between hUC-MSC-activated (red) and -repressed genes (green) and neuronal module (blue) and microglial and endothelial module (orange). (E) Gene Set Enrichment Analysis (GSEA) showing the association of the hUC-MSC-activated and -repressed genes with the genes highly expressed in neurons (N), myelinating oligodendrocytes (MO), astrocytes (A), microglia (MG) as well as endothelial cells (EC), respectively.

To explore the molecular mechanisms underlying the therapeutic effects of hUC-MSC transplantation on subacute SCI and identify the possible direct and/or indirect targets of hUC-MSCs in the damaged spinal cord, we carried out extensive transcriptome analyses of spinal cord tissues at the lesion site from the subacute phase of SCI (sub-SCI) either treated without (+PBS) or with hUC-MSCs (+MSC) at 0 (sub-SCI), 1, 2 and 4 weeks post-transplantation (wpt), as well as comparable spinal cord segments of intact/sham rats (Intact) (Figure 1A). The survival of hUC-MSC xenografts in the rat spinal cord was monitored by *in vivo* imaging of lipophilic fluorescent dye-labeled MSCs (Figure S1A) and quantified by the amplification of human-specific gene *Xrcc5* at day 10 (d10) and day 20 (d20) post-transplantation (Figure S1B). The beneficial effects of hUC-MSCs were evaluated by a series of behavioral analyses at one-week interval for 4 weeks (Figure S1C) and motor evoked potentials (MEPs) recording of hindlimb at the endpoint (Figure S1D-E), both indicating the functional recovery based on the reestablishment of neuronal relay. It is worth noting that, to rule out the possible sequence contamination resulting from the residual hUC-MSCs in the rat spinal cord at 4 wpt, the clean reads were first mapped to the human reference genome to filter out the human reads, thereafter, the filtered non-human reads were further aligned to the rat reference genome in order to obtain the data generated from the rat spinal cord. Multiple pairwise comparisons were performed and total 7329 differentially expressed genes (DEGs) (with |log_2_FC| > 1 and adjusted *p* < 0.01) were identified based on three contrasts, sub-SCI *vs*. Intact, +PBS *vs*. Intact, and +MSC *vs.* +PBS at different time points. Both the principal component analysis (PCA) and unbiased hierarchical clustering of the top 5000 DEGs revealed that +MSC samples were clustered more closely to intact ones compared to that in the +PBS group, indicating hUC-MSC treatment reversed the SCI-associated gene signature toward that of intact spinal cord (Figure S1F-G).

To further unravel the possible therapeutic targets of hUC-MSCs in the injured spinal cord, we performed the comprehensive comparisons to identify DEGs reversed by hUC-MSC transplantation (Figure 1A, bioinformatics). We defined the genes downregulated by SCI (+PBS* vs*. Intact) but upregulated by the MSC treatment (+MSC *vs*. +PBS) at different time points as hUC-MSC-activated genes, which may be associated with the normal physiological function of spinal cords and hUC-MSC-mediated recovery; and the genes upregulated by SCI (+PBS* vs*. Intact) but downregulated by MSC treatment (+MSC *vs*. +PBS) at different time points as hUC-MSC-repressed genes, which may be involved in pathological events induced by SCI. A total of 224, 61, and 227 hUC-MSC-activated genes and 107, 173, and 16 hUC-MSC-repressed genes were identified at 1, 2, and 4 wpt, respectively, suggesting most of the MSC-reversed genes are associated with returning functional impairments to normal (Figure 1B). Using this approach, we identified 704 hUC-MSC-reversed genes, among which 429 (60.9%) were activated while 275 (39.1%) were repressed (Figure 1C, left). Unbiased hierarchical sample clustering demonstrated that the intact group clustered together with all three +MSC-2w samples and one +MSC-4w sample, whereas sub-SCI group was much closer to +PBS group than +MSC group. One +PBS-2w sample clustered with +MSC-2w as an outlier may represent better spontaneous recovery from certain individual. Gene Ontology (GO) enrichment analysis revealed that hUC-MSC-activated genes were involved in neurons/synaptic transmission, while hUC-MSC-repressed genes were involved in inflammatory response as well as glial scar formation, axonal degeneration and demyelination (Figure 1C, right and Table S1). Kyoto Encyclopedia of Genes and Genomes (KEGG) pathway analysis revealed the top enriched signaling pathways for hUC-MSC-activated and -repressed genes are neuroactive ligand-receptor interaction (adjusted* p* = 2.52×10^-13^) and ECM-receptor interaction (adjusted* p* = 7.35×10^-5^), respectively (Table S1). These results indicated that the hUC-MSC-mediated restoration of spinal functions was mainly through anti-inflammation response, reducing glial scar formation, and maintaining neurotransmission to prevent the destruction of the neural circuits after SCI.

Next, we investigated the relationships between these two gene sets and two highly conserved gene coexpression modules of human spinal cord 13, *i.e.*, a neuronal module implicated in the synaptic transmission and a microglial and endothelial module related to inflammatory and wound responses, respectively. The venn diagrams showed that the hUC-MSC-activated and -repressed genes were significantly associated with the neuronal module (*p* = 1.19×10^-38^) and the microglial and endothelial module (*p* = 8.73×10^-9^), respectively (Figure 1D). Altogether, hUC-MSC-activated and -repressed genes are functionally over-represented the genes associated with the neuronal regeneration and inflammation/scar formation, respectively.

To further confirm that hUC-MSC-activated and -repressed genes exhibited distinct functions, we employed the Gene Set Enrichment Analysis (GSEA) for biological characterization of the hUC-MSC-reversed genes. We compared the hUC-MSC-activated and -repressed gene sets to the cell-type specific gene expression profiles of neurons, myelinating oligodendrocytes (MOs), astrocytes, microglia and endothelial cells 14, correlating with the phenotypic expression signature of neuronal protection and inflammation/scar formation. As shown in Figure 1E, hUC-MSC-activated genes are significantly enriched in genes that expressed in the neurons and MOs, whereas hUC-MSC-repressed genes are highly associated with the genes predominantly expressed in astrocytes, microglia and endothelial cells. In summary, hUC-MSC-activated and -repressed genes are functionally segregated and associated with neuronal protection, inflammation and extracellular matrix/scar formation, respectively.

### The GABAergic synapse pathway is significantly enriched in hUC-MSC-activated genes in the injured spinal cord

**Figure 2:**
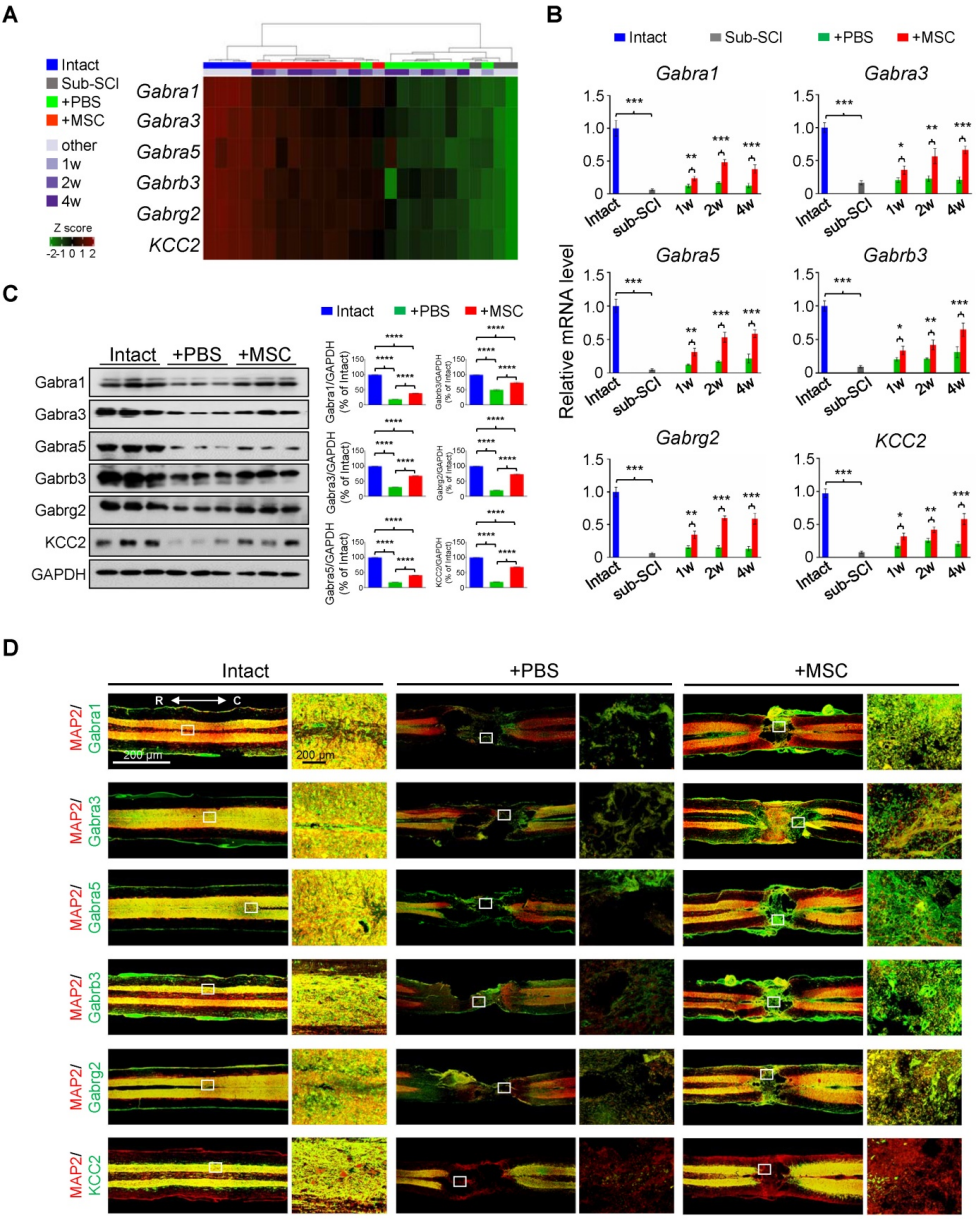
**GABA_A_R subunits are highly enriched in hUC-MSC-activated genes.** (A) Heatmap of the hierarchical clustering of the GABA_A_R subunits and KCC2 across all samples. (B) Time course of real-time PCR analysis of GABA_A_R subunits in the rat model of subacute SCI treated with or without hUC-MSCs (n = 3/group/time point). The data are presented as mean ± SEM. *p < 0.05; **p < 0.01; ***p < 0.001 by two-way ANOVA. (C) Left, Western blot analysis to measure the protein levels of Gabra1, Gabra3, Gabra5, Gabrb3, Gabrg2 and KCC2 in the injured rat spinal cord treated without or with hUC-MSCs at 4 wpi. GAPDH served as an internal control. Right, quantitative analyses of Western blotting (n = 3/group). Data are presented as mean ± SEM. ****p < 0.0001 by one-way ANOVA. (D) Representative IF images of coronal sections across the lesion epicenter stained for MAP2 (green), different GABA receptor subunits (red) and KCC2 (red) in indicated groups at 4 wpt. R, rostral; C, caudal. Scale bar, 200 μm. The images shown are representative of six slides from three rats of each group.

In light of the extensively investigated gene expression profiles of pathological events after SCI, we particularly focused on the hUC-MSC-activated genes to explore the potential therapeutic targets of MSCs in the injured spinal cord. Intriguingly, among the significantly enriched GO terms activated by the hUC-MSC transplantation, many GABA receptor subunit genes were present, such as the ion channel complex (adjusted *p* = 2.93×10^-30^), GABAergic synapse (adjusted* p* = 6.68×10^-19^), and GABA-gated chloride ion channel activity (adjusted *p* = 4.58×10^-5^) (Table S2). We identified 12 GABA receptor subunits and KCC2 as hUC-MSC-activated genes (Figure 2A and Figure S2A), in which GABA_A_R subunits *Gabra1*, *Gabra3*, *Gabra5*, *Gabrb2*, *Gabrb3*, and *Gabrg2* are preponderant in the spinal cord 15. The role of GABA_A_Rs and GABAergic transmission in neuropathic pain and spasticity has been extensively investigated 16-18. However, their roles in promoting the functional recovery of SCI as a coordinator of KCC2 to maintain the excitation/inhibition (E/I) balance have been underappreciated in mammals. Considering this, we selected *Gabra1*, *Gabra3*, *Gabra5*, *Gabrb3*, *Gabrg2* as candidate genes for further investigation, and *KCC2* served as a positive control (Figure 2A). First, the repression of these GABA_A_R genes by SCI and the reversal induction by hUC-MSC transplantation at different time points were validated by real-time qPCR (Figure 2B and Figure S2A) and Western blotting (Figure 2C). To further visualize the expression of GABA_A_R subunits in the injured spinal cord without or with hUC-MSC transplantation, we carried out the immunofluorescence (IF) staining to the spinal sections through the lesion epicenter (Figure 2D and Figure S2B). In the intact spinal cord, a widespread distribution of the five GABA_A_R subunits in the gray matter was observed, partially overlapping with the neuronal markers (NeuN and MAP2). KCC2 expression was mainly localized in dendrites and axons in the gray matter, but in dendrites penetrating the white matter other than cell soma. SCI caused a remarkable reduction of all subunits. In contrast, hUC-MSC transplantation partially rescued the decrease in GABA_A_R subunits as well as KCC2. Taken together, *GABA_A_Rs* as hUC-MSC-activated genes are highly enriched and act as potential targets of hUC-MSCs.

### Bicuculline compromises the hUC-MSC-mediated functional recovery

**Figure 3:**
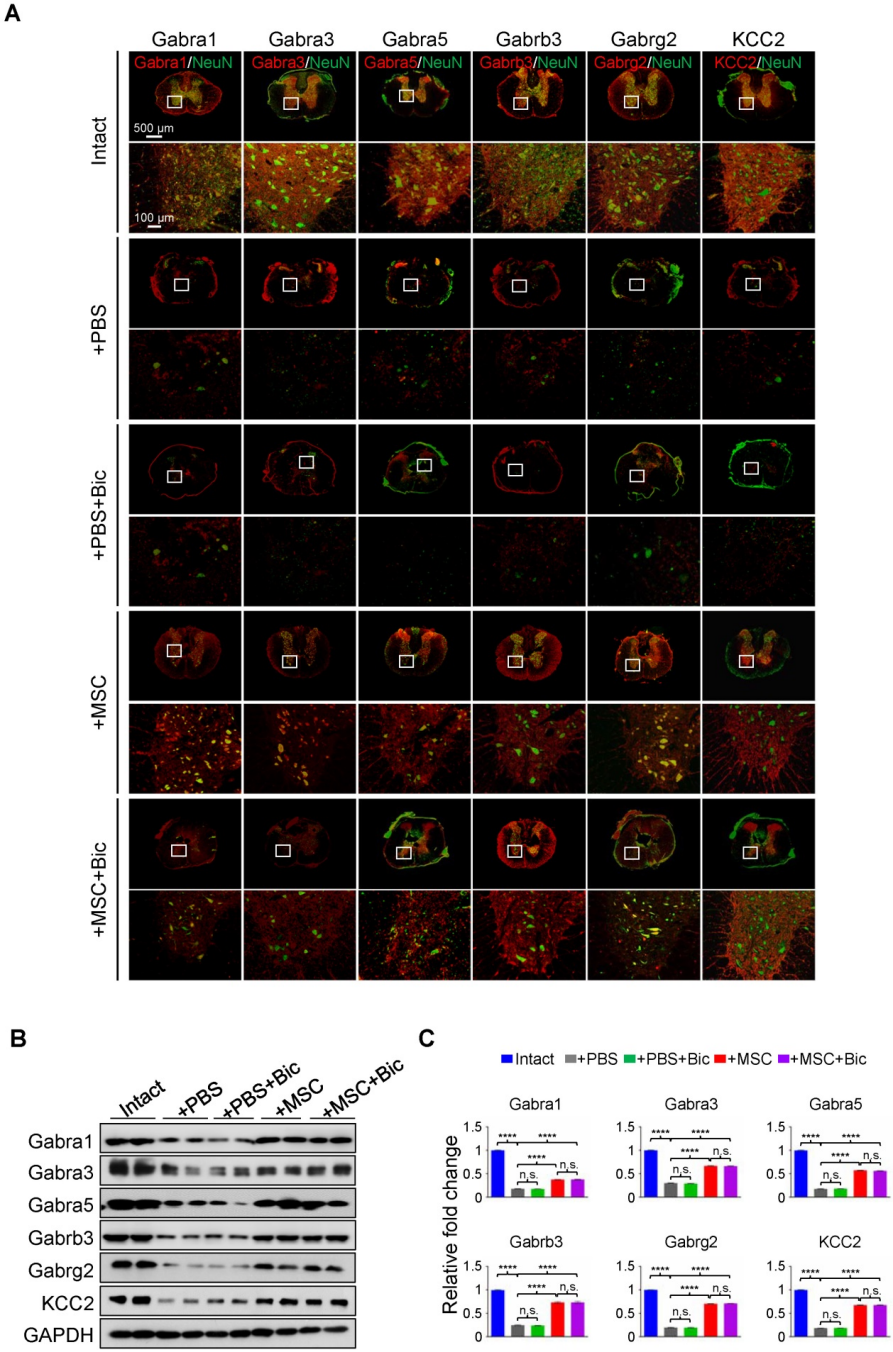

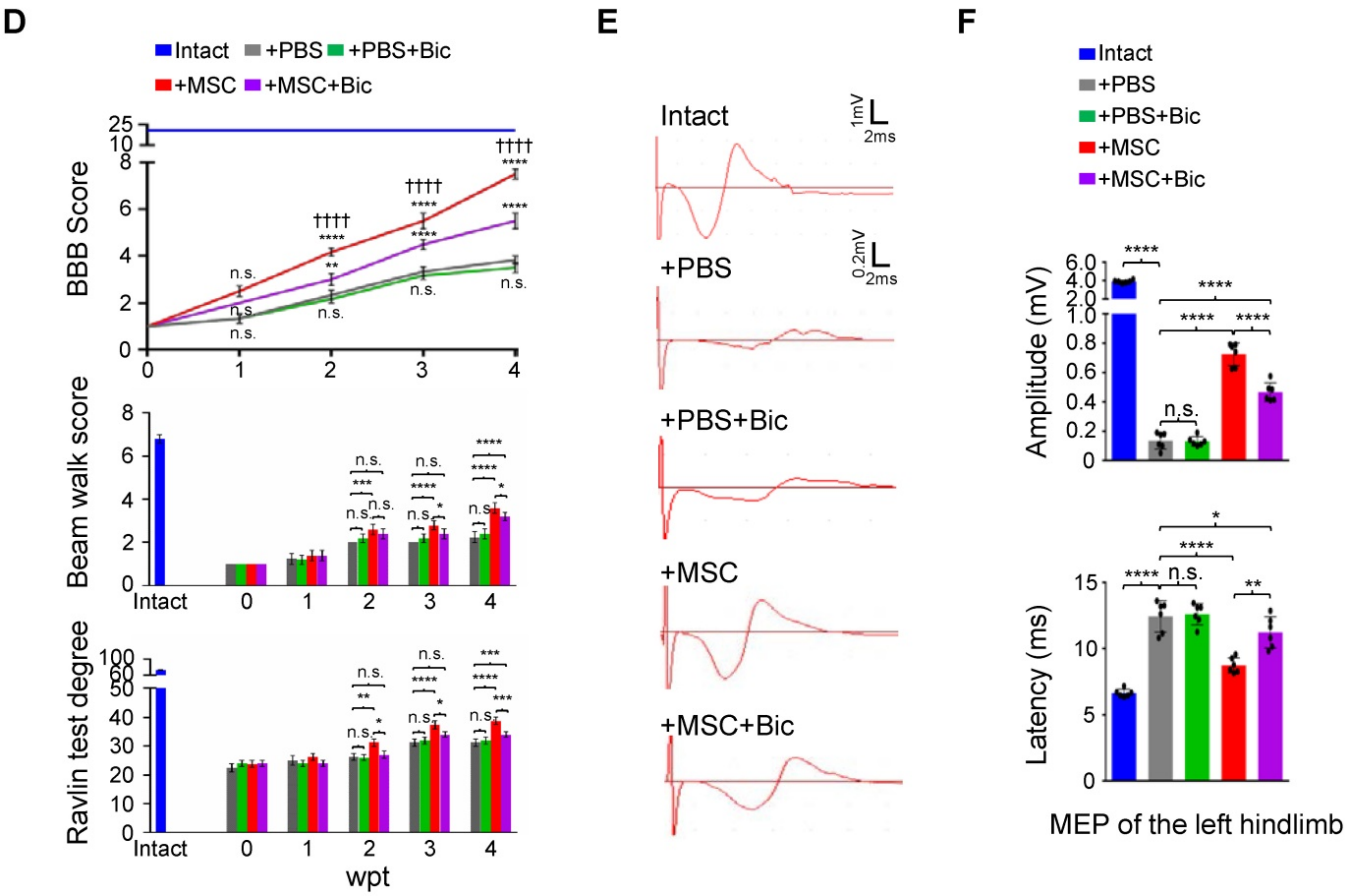
** hUC-MSC-mediated functional recovery of SCI is attenuated by bicuculline treatment.** (A) Representative IF images of transverse spinal cord sections from the Intact, +PBS, +PBS+Bic, +MSC, and +MSC+Bic groups at 4 wpt. Double immunostaining of NeuN (green) with Gabra1, Gabra3, Gabra5, Gabrb3, Gabrg2 and KCC2 (red). Bic, bicuculline. Lower panels, higher-magnification view of the white rectangle in main panels. Scale bar, 500 μm (main panels); 100 μm (lower panels). The images shown are representative of six slides from three rats of each group. (B) Western blot analyses of Gabra1, Gabra3, Gabra5, Gabrb3, Gabrg2, KCC2 and GAPDH in the lesion sites from different groups at 4 wpt. (C) Quantification of B (n = 3/group). Data are presented as mean ± SEM. n.s., not significant; ****p < 0.0001 by one-way ANOVA. (D) A series of behavioral tests to evaluate the effects of bicuculline treatment on hUC-MSC-mediated functional recovery (n = 12/group/time point). The color key is used throughout all panels. Data are presented as mean ± SEM. n.s., not significant; *p < 0.05; **p < 0.01; ***p < 0.001, ****p < 0.0001 by two-way ANOVA. ††††*p* < 0.0001 refers to +MSC *vs.* +MSC+Bic. (E) Representative MEP recordings of rats with different treatments after SCI. (F) Quantification and statistical analyses of amplitude and latency period of MEPs (n = 6/group). The data are presented as mean ± SEM. n.s., not significant, *p < 0.05, **p < 0.01, ****p < 0.0001 by one-way ANOVA.

Previous studies have shown that pharmacological targeting of GABA_A_Rs can alleviate neuropathic pain in a peripheral nerve injury model 19, yet no evidence has been provided regarding the role of any GABA_A_R subunit on hindlimb functional recovery in SCI rats. We first applied sustained administration of bicuculline, a competitive antagonist of GABA_A_Rs 20, either with or without hUC-MSC transplantation to assess the effects of GABA_A_Rs on hUC-MSC-mediated SCI repair. IF staining showed that the general expression levels and/or distributions of different GABA_A_R subunits were not obviously affected by bicuculline treatment at the epicenter (Figure 3A). The quantitative analysis of Western blotting also revealed that there was no significant difference in the protein levels of all GABA_A_R subunits between treatments with and without bicuculline under SCI or MSC transplantation conditions (Figure 3B-C). However, bicuculline administration significantly diminished the hUC-MSC-mediated improvement of locomotor function and MEP in SCI rats with barely any effect on the spontaneous recovery, suggesting the disturbing influence of GABA_A_R antagonist on the enhanced relay efficiency of spinal circuitry promoted by hUC-MSCs (Figure 3D-F). Altogether, our results demonstrate that the effects of hUC-MSC transplantation on functional recovery were restrained in the presence of bicuculline without affecting the protein levels of GABA_A_Rs.

### Overexpression of GABA_A_R subunits in neurons partially mimics the functional recovery mediated by hUC-MSC transplantation after SCI

**Figure 4:**
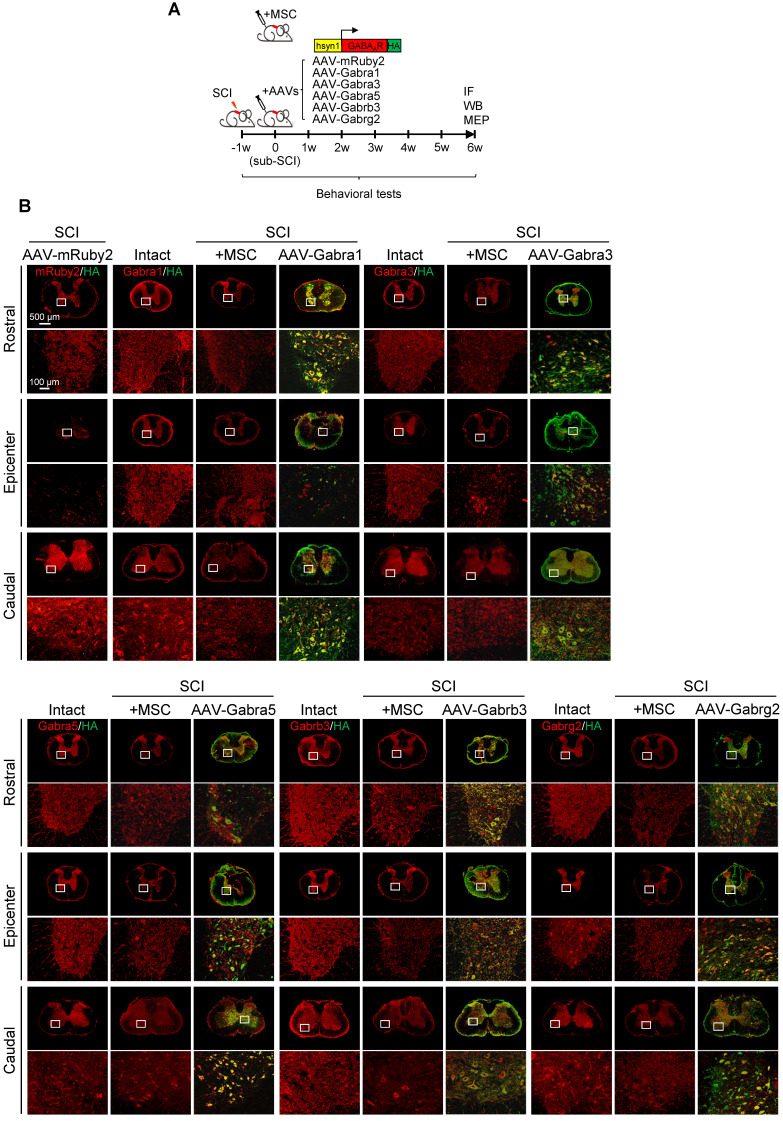

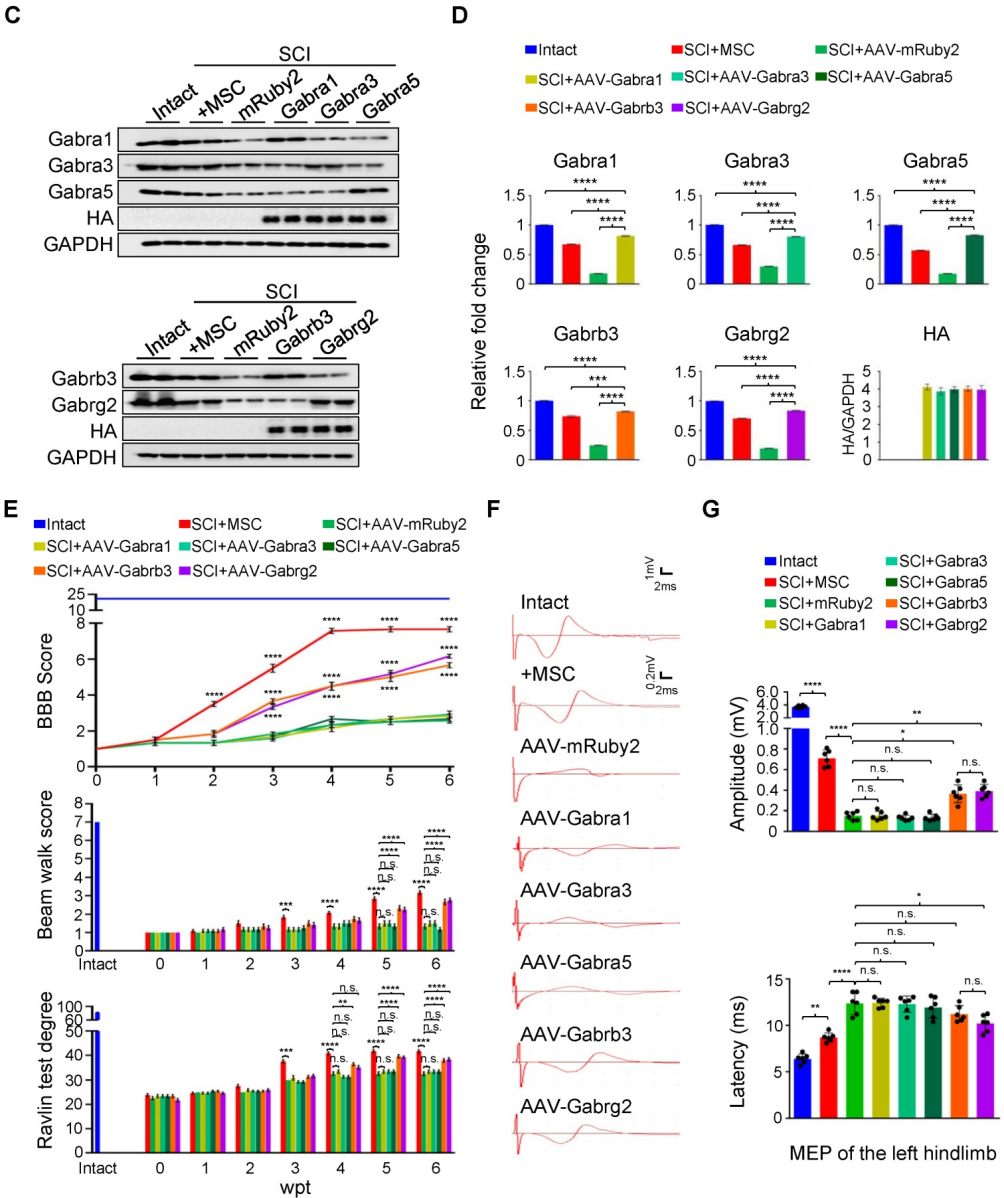
** GABA_A_R subunit-overexpressing in spinal neurons partially mimics the functional recovery mediated by hUC-MSC transplantation.** (A) Schematic for intrathecal AAV-mediated gene delivery to neurons of the injured spinal cord. (B) Representative IF images of transverse spinal cord sections co-immunostained with individual anti-GABA_A_R (red) and anti-HA (green) antibodies at T2 (Rostral), T10 (Lesion Epicenter) and L3 (Caudal) levels in rats with indicated treatments 6 weeks after injection. Lower panels, higher magnification view of the region indicated by the white rectangle in upper panels. Scale bar, 500 μm (upper panel), 100 μm (lower panel). (C) Western blot analyses of Gabra1, Gabra3, Gabra5, Gabrb3, Gabrg2, KCC2 and GAPDH in the lesion sites from different groups. (D) Quantification of C (n = 3/group). Data were presented as mean ± SEM. ****p < 0.0001 by one-way ANOVA. (E) Behavioral tests to evaluate the effects of AAV-mediated GABA_A_R subunit overexpression on functional recovery compared with hUC-MSC transplantation after SCI (n = 12/group/time point). The color key is used throughout all panels. Data are presented as mean ± SEM. n.s., not significant; *p < 0.05; **p < 0.01; ***p < 0.001, ****p < 0.0001 between the AAV-mRuby2 and other groups by two-way ANOVA. (F) Representative MEP recordings of rats with different treatments after SCI. (G) Quantification and statistical analyses of F (n = 6/group). The data are presented as mean ± SEM. n.s., not significant, *p < 0.05, **p < 0.01, ***p < 0.001, ****p < 0.0001 by one-way ANOVA.

Given that hUC-MSCs boosted the expression of GABA_A_R subunits in the injured spinal cord, while the GABA_A_R antagonist significantly attenuated hUC-MSC-mediated locomotor recovery, we hypothesized that targeting GABA_A_Rs could provide a feasible strategy for SCI repair. To test this hypothesis and gain further insights into the role of hUC-MSC-mediated GABA_A_R upregulation, we first tempted to clarify that overexpression of certain subunit *in vivo* could mimic the functional recovery mediated by hUC-MSCs. We employed the AAV-PHP.eB capsids developed by Gradinaru's group 21 to deliver a gene of interest under human synapsin 1 (hsyn1) promoter for highly efficient transduction of neurons throughout the rat spinal cord (Figure 4A). To distinguish the exogenous gene expression from the endogenous protein, each GABA_A_R subunit was HA-tagged at the C-terminal. The intrathecal AAV injection was performed at the L6 level of rat spinal cord one week after SCI. AAV-mRuby2 served as a negative control. The IF staining with both anti-HA and anti-GABA_A_R subunit antibodies was performed to verify the overexpression and distribution of each GABA_A_R subunit at the T2, T10 and L3 levels of rat spinal cords, representing the rostral, epicenter and caudal locations, respectively. Generally, a widespread expression pattern of the five GABA_A_R subunits in the gray matter was observed and no obvious differences in the signal intensity and distribution for each GABA_A_R subunit were detected between T2 and L3 levels of the intact spinal cord (Figure 4B, Intact panels). SCI caused the markedly downregulation of all subunits at the lesion epicenter and moderate decrease in caudal locations compared to the intact control (Figure 4B, SCI panels). The AAV treatment significantly increased the GABA_A_R subunit expression, especially in the region of soma and proximal dendrites of neurons compared to their original endogenous localization (Figure 4B, high magnification images). Western blotting also confirmed the successful overexpression of each subunit, to an extent of ~80% of the intact tissue and significantly higher than that of the MSC transplantation (Figure 4C-D). To monitor the locomotor activity of hindlimbs, we then performed weekly behavioral tests for 6 weeks post-treatment (Figure 4E). In five different GABA_A_R subunit-overexpressing groups, significantly higher BBB scores were observed only in the AAV-Gabrb3 and AAV-Gabrg2 groups as early as 3 weeks after injection compared to that of the AAV-mRuby2 control, which was one week later than that of MSC transplantation. The functional improvements of AAV-Gabrb3 and AAV-Gabrg2 were gradually increased to a less extent than that of hUC-MSCs at the endpoint, probably because 2-4 weeks were necessary for AAV to achieve a steady-state expression level. Consistent with the behavioral results, AAV-Gabrb3 and AAV-Gabrg2-treated SCI rats exhibited significant increment in MEP activity in hindlimb to the cortical stimulation compared to mRuby2 and three other subunits (Figure 4F-G). However, the extent of amplitude increment and latency decrement of MEP signals in SCI rats treated with Gabrb3 and Gabrg2 was significantly lower than that of MSC transplantation, indicating that the restoration of GABA_A_R subunit expression is one of the mechanisms underlying hUC-MSC-mediated functional recovery. Thus, the neuron-specific overexpression of GABA_A_R subunits β3 and γ2 counteract the SCI-induced depletion, leading to significant functional recovery to an extent less than hUC-MSCs. To the best of our knowledge, this study, for the first time, has established a connection between hUC-MSC-mediated locomotor recovery and the activity of GABA_A_Rs after SCI in mammals.

### Restoration of KCC2 expression in combination with GABA_A_Rs enhances the functional recovery

**Figure 5:**
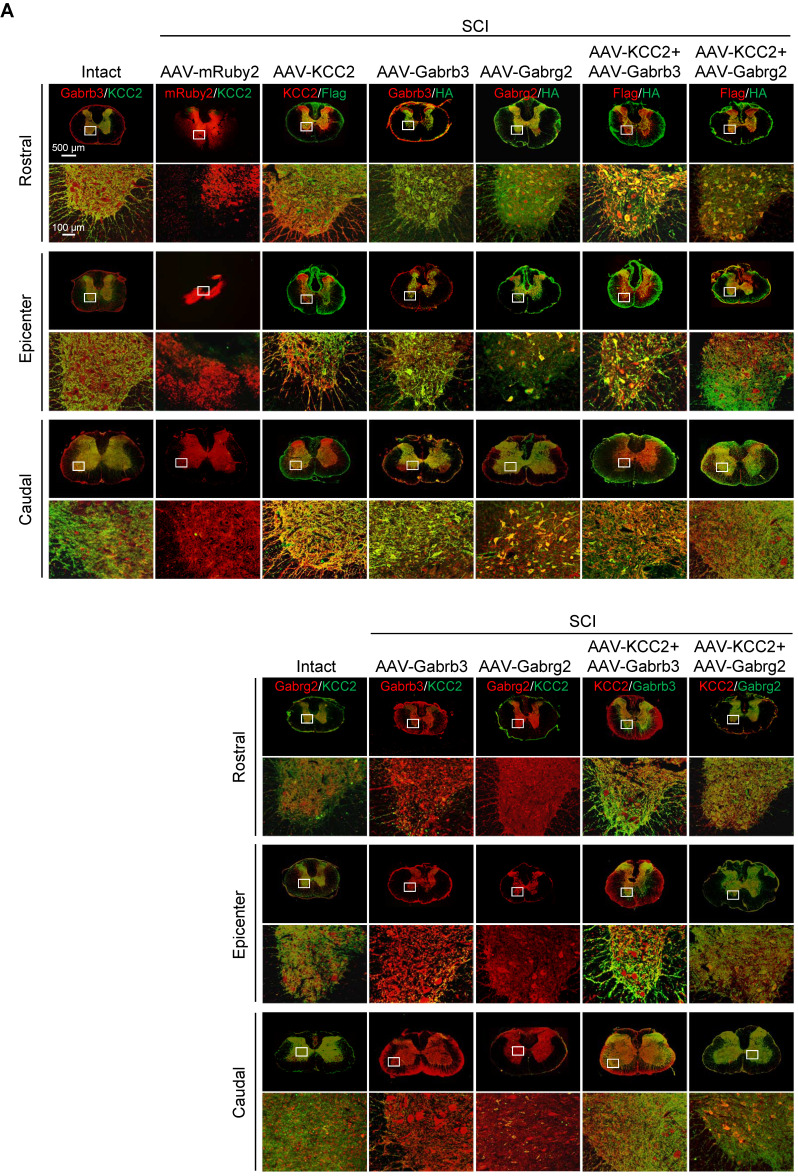

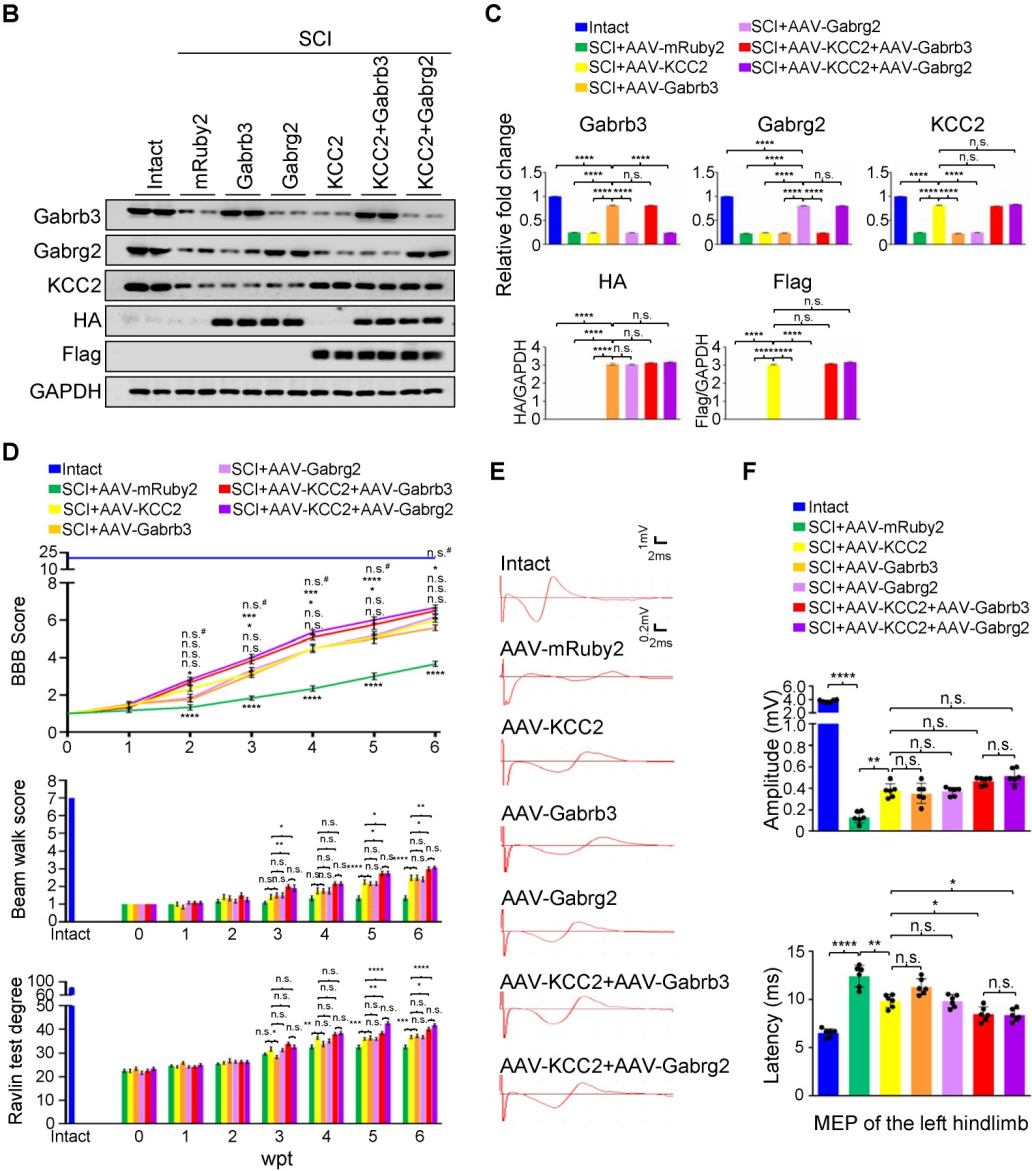
**Superior effects of the combination of GABA_A_R subunits and KCC2 on subacute SCI.** (A) Representative IF images of transverse spinal cord sections at T2 (Rostral), T10 (Lesion Epicenter) and L3 (Caudal) levels in rats with indicated treatments, immunostained with antibodies specifically targeting individual GABA_A_R, HA-tag and Flag-tag 6 weeks after injection. Lower panels, higher magnification view of the region indicated by the white rectangle in upper panels. Scale bar, 500 μm (upper panel), 100 μm (lower panel). (B) Western blot analyses of Gabrb3, Gabrg2 protein levels with protein-specific and anti-HA antibodies, and KCC2 with anti-KCC2 and anti-Flag antibodies in the lesion sites from different groups 6 weeks after injection. GAPDH served as an internal control. (C) Quantification of B (n = 3/group). The data are presented as mean ± SEM. n.s., not significant; ****p < 0.0001 by one-way ANOVA. (D) Behavioral tests to compare the effects of AAV-mediated GABA_A_R subunit overexpression alone and together with KCC2 on the functional recovery of SCI (n = 12/group/time point). The data are presented as mean ± SEM. For BBB score, n.s., not significant; *p < 0.05; ***p < 0.001, ****p < 0.0001 refers to SCI+AAV-KCC2 *vs.* other groups. n.s.^#^, not significant refers to SCI+AAV-KCC2+AAV-Gabrb3 *vs.* SCI+AAV-KCC2+AAV-Gabrg2. For Beam walk and Ravlin test, n.s., not significant; *p < 0.05; **p < 0.01; ***p < 0.001, ***p < 0.0001 by two-way ANOVA. (E) Representative MEP recordings of rats with different treatments after SCI. (F) Quantification and statistical analyses of the amplitude and latency of MEPs (n = 6/group). The data are presented as mean ± SEM. n.s., not significant, *p < 0.05, **p < 0.01, ***p < 0.001, ****p < 0.0001 by one-way ANOVA.

KCC2 is a crucial regulator of GABA-mediated hyperpolarization. In mature neurons, the increased expression of KCC2 leads to low [Cl^-^]_i_, which cooperates with GABA_A_R activation to allow Cl^-^ influx leading to hyperpolarization 11. A recent study has shown both pharmacological enhancement and exogeneous expression of KCC2 in inhibitory interneurons promote the functional recovery of mouse model with SCI through reactivating the spared dormant relay circuits 22. Considering that the hUC-MSC treatment also increased the expression of KCC2 in the injured spinal cord, and the two GABA_A_R subunits were both capable of improving hindlimb locomotor performance after SCI, it is plausible to speculate that their combinations could further benefit Cl⁻ homeostasis in neurons that may result in better neurological recovery. We used AAV injection to deliver Flag-tagged KCC2 alone or in combination with HA-tagged Gabrb3 and Gabrg2 in rats with sub-SCI, respectively, and assessed the outcomes among different groups. The results showed that endogenous KCC2 was mainly colocalized with Gabrb3 and Gabrg2 in dendrites and axons other than cell soma as shown before (Figure 5A, Intact panels). SCI dramatically depleted the expression of three proteins which was shown in SCI rats injected with AAV-mRuby2 (Figure 5A, AAV-mRuby2 panels). AAV-KCC2 distribution pattern was mostly the same as the endogenous KCC2, while with more signals in cell soma. The quantitative analysis of Western blotting showed that the individual or combinatorial AAV-mediated overexpression restored the expression of KCC2, Gabrb3, and Gabrg2 to the levels closer to the intact group (Figure 5B-C). Over 6 weeks after treatment, KCC2 overexpression alone demonstrated a similar extent of beneficial effects on locomotor (Figure 5D) and electrophysiological (Figure 5E-F) recoveries as two effective GABA_A_R subunits. When combined with either of the two GABA_A_R subunits, KCC2 showed superior effects than the individual overexpression (Figure 5D). Interestingly, both combinatorial treatments slightly but significantly shortened the latency without affecting the amplitude of MEP, compared to the individual treatment (Figure 5E-F), suggesting the pivotal role of synergistic cooperation of GABA_A_R subunits and KCC2 in maintaining the accurate neuronal E/I balance to facilitate the relay efficiency of spinal circuitry. No differences between the two combinatorial AAV treatments in terms of both behavioral and MEP signals were detected, indicating KCC2 holds similar effects on GABA_A_R subunit β3 and γ2. Thus, co-expression of GABA_A_R β3 or γ2 together with KCC2 contributes to better functional recovery of SCI, suggesting a synergistic effect of GABA_A_Rs and KCC2 on reducing the neuronal excitability is a powerful strategy to enhance responsiveness to descending inputs, and to promote functional recovery of severe SCI.

### BDNF secreted from hUC-MSCs contributes to the induction of GABA_A_Rs in mature neurons after axonal lesion *in vitro*

What is the mechanism of the hUC-MSC-mediated upregulation of GABA_A_R subunits after transplantation? BDNF is well-known to regulate GABA_A_R gene transcription and it is one of the major neurotrophic factors, secreted by MSCs to exert neuroprotection including preventing apoptosis and supporting axonal growth 23-25. To assess whether hUC-MSCs rescue the depletion of GABA_A_R subunits through BDNF secretion in mature neurons after injury, we conducted the mature cortical axon scrape assay at day 21 *in vitro* (DIV) and co-cultured injured neurons with hUC-MSCs in the presence or absence of BDNF knockdown for another 7 days (Figure S3A). The BDNF knockdown efficiency was confirmed in shluc and shBDNF lentiviral-transduced hUC-MSCs before the scrape assay at both the mRNA and protein levels (Figure S3B-C). The axon regeneration indicated by βIII-tubulin and the expression levels of GABA_A_R β3, γ2 and KCC2 were monitored by IF staining. As shown in Figure S3D, few cortical axons regenerated spontaneously 7 days after axotomy. 50 ng/ml recombinant human BDNF (rhBDNF) treatment, as the positive control, significantly increased cortical axon regeneration. hUC-MSCs promoted the axonal regeneration to a less extent than that of rhBDNF, whilst BDNF knockdown significantly abolished the regenerative effect (Figure S3D-E). The expression of Gabrb3, Gabrg2 and KCC2 was much more enhanced in the cell soma or dendrites in the non-scratched zone with rhBDNF and hUC-MSC treatments compared to that without MSC or MSC-shBDNF treatments, indicating the significant role of BDNF in restoration of decreased GABA_A_R subunits and KCC2 expression after the injury. Although hUC-MSCs promoting the axonal regeneration were demonstrated by more βIII-tubulin-positive axons in the scratched zone, the regenerated axons were GABA_A_R and KCC2 double-negative, probably because of the short synapse formation during the early stage of axon regrowth 26. Thus, hUC-MSC-mediated restoration of GABA_A_R and KCC2 expression in injured mature neurons could be attributed to the secretion of BDNF.

### BDNF secreted by hUC-MSCs partially rescues the decrease in GABA_A_R subunits and improves the functional recovery of SCI *in vivo*

**Figure 6:**
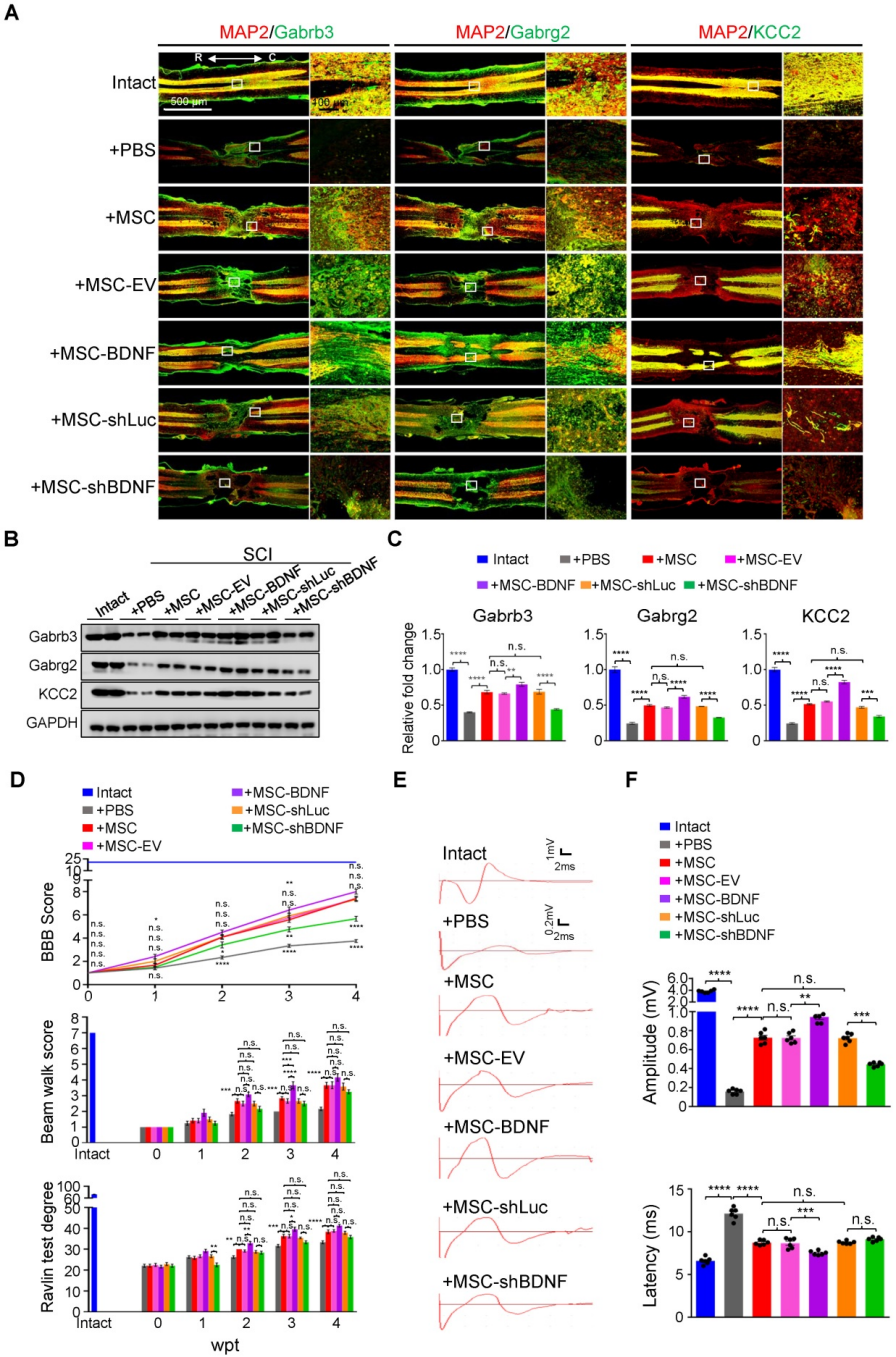
** hUC-MSCs partially rescue the SCI-induced depletion of GABA_A_R subunits through BDNF secretion *in vivo*.** (A) Representative IF images of coronal spinal cord sections around the lesion epicenter from indicated groups at 4 wpt. Double immunostaining of MAP2 (red) with Gabrb3, Gabrg2, and KCC2 (green). EV, empty vector; R, rostral; C, caudal. Right panels, higher-magnification images of the white rectangle in the left panels. Scale bar, 500 μm (left); 100 μm (right). The images shown are representative of six slides from three rats of each group. (B) Western blot analyses of Gabrb3, Gabrg2, KCC2, and GAPDH in the lesion sites from different groups at 4 wpt. (C) Quantification of B (n = 3/group). Data are presented as mean ± SEM. n.s., not significant; **p < 0.01; ***p < 0.001; ****p < 0.0001 by one-way ANOVA. (D) A series of behavioral tests to evaluate the effects of BDNF on the hUC-MSC-mediated functional recovery (n = 12/group/time point). The color key is used throughout all panels. Data are presented as mean ± SEM. n.s., not significant; *p < 0.05; **p < 0.01; ***p < 0.001; ****p < 0.0001 by two-way ANOVA. (E) Representative MEP recordings of rats from different groups at 4 wpt. (F) Quantification and statistical analyses of amplitude and latency of MEPs (n = 6/group). The data are presented as mean ± SEM. n.s., not significant; **p < 0.01; ***p < 0.001; ****p < 0.0001 by one-way ANOVA.

To further prove that BDNF secreted by hUC-MSCs is capable of reversing the downregulation of GABA_A_R subunits and KCC2 in the injured spinal cord, we transplanted the SCI rats with genetically programmed hUC-MSCs with either overexpression (MSC-BDNF) or knockdown (MSC-shBDNF) of BDNF parallelly to their controls, *i.e.*, MSCs-empty vector (MSC-EV) or MSC-shLuc (Figure 6A-C and Figure S4). As expected, three MSC controls (MSC, MSC-EV, and MSC-shLuc) achieved similar effects on SCI repair. Compared to controls, BDNF overexpression further enhanced the therapeutic effects of hUC-MSCs which was significantly compromised by BDNF knockdown (Figure 6E-F). Notably, BDNF knockdown only significantly decreased the elevated amplitude without increasing the latency of MEP recordings compared to MSC controls, supporting that BDNF produced by hUC-MSCs promotes not only propriospinal relay connections but also neural regeneration. Therefore, BDNF secreted by hUC-MSCs ameliorates the reduction of GABA_A_R subunits as well as KCC2 in the injured spinal neurons and leads to superior locomotor performance after SCI.

## Discussion

Studies from our group and others have demonstrated the application of hUC-MSCs to facilitate the reestablishment of spinal circuit and functional recovery of incomplete SCI in animal models and humans 5, 6. It is generally acknowledged that the therapeutic effect of MSC transplantation for SCI is mainly based on their remarkable paracrine activity. However, few studies have focused on the spinal transcriptome from the perspective of hUC-MSC-mediated recovery to elucidate the molecular mechanisms, through which MSCs orchestrate the neural protective functions. The growing interest in unravelling the mechanisms of hUC-MSCs promoting SCI repair inspired us to conduct a time-course transcriptome analysis of spinal cord tissues at the lesion site from a subacute phase of SCI treated without or with hUC-MSCs. We screened hUC-MSC-reversed genes, which may represent the hUC-MSC-mediated recovery by comparing spinal cord transcriptomes with and without cell transplantation. Consistent with previous reports, hUC-MSC transplantation represses genes involved in the extracellular structure organization, leukocyte migration, response to wounding, and inflammation response, which may contribute to reducing glial scar formation and eliminating inflammation. Surprisingly, we have identified the GABAergic synapse pathway as one of the major targets of hUC-MSC-activated genes. Notably, several GABA_A_R subunit genes are highly induced in a hUC-MSC-dependent manner. The effect of hUC-MSCs on restoration of locomotor function can be attenuated by the daily injection of GABA_A_R antagonist, *i.e.,* bicuculline. Moreover, using an AAV-mediated *in vivo* overexpression approach, we have found that specific overexpression of Gabrb3 and Gabrg2 in neurons of the spinal cord leads to significant functional recovery in rats with subacute SCI. In addition, combinatorial overexpression of either the GABA_A_R subunits with KCC2 results in further improvement compared with the receptor subunit gene alone, suggesting the essential roles of neuronal Cl⁻ homeostasis and GABAergic inhibition mediated by the coordinated upregulation of KCC2 and GABA_A_Rs in SCI repair. Our results have introduced a brand-new mechanism in which hUC-MSCs synergistically upregulate GABA_A_Rs and KCC2 through BDNF secretion, to facilitate the reestablishment of spinal circuit after SCI.

Although several recent efforts have depicted the biological/pathological processes post-SCI at transcriptional levels through high-throughput sequencing 1-4, little is known on pivotal therapeutic targets for the restoration of impaired spinal function yet. To unravel the possible direct and/or indirect therapeutic targets of hUC-MSCs in the injured spinal cord, we compared the transcriptome of injured spinal cords treated with or without hUC-MSCs at various time points. Firstly, based on our studies, a few but detectable hUC-MSCs survived in the injured rat spinal cord at day 20 post-transplantation (Figure S1A-B) 27. To distinguish confounding reads of the residual hUC-MSCs from that of the rat spinal cord at 4 wpt, we mapped the clean reads to the human reference genome to filter out the human reads before alignment to the rat reference genome. Generally, less than 1% of the sequence reads of each library were aligned to the human genome and more than 85% of sequence reads produced by this double-genome alignment pipeline were rat sequences, suggesting limited survival of hUC-MSC xenografts in rats. Secondly, we used stringent criteria that genes with |log_2_FC| > 1 and adjusted *p* < 0.01 found by DESeq2 were considered as DEGs to obtain confidential targets. Furthermore, we did not perform simple comparisons between all treated groups (+PBS and +MSC) and the intact group but developed a comparative analysis strategy aimed to identify the possible spinal targets modulated by the hUC-MSC transplantation (Figure 1A). According to our results, most of the hUC-MSC-reversed genes, including both activated and repressed genes, were overlapped with SCI-regulated genes (Figure 1B), indicating the successful screening.

The Cl⁻ homeostasis and GABAergic system play important roles in maintaining spinal integrity and performance. Normal motor function depends on the GABAergic signaling in the spinal cord, and the rough pharmacological blockade of GABA_A_Rs by bicuculline treatment could disrupt motor function regardless of MSC-enhanced recovery. It is noteworthy that the dosage of bicuculline used in the current study was subthreshold for seizures according to the literature 28-30. Regarding bicuculline-induced convulsions, the subcutaneous doses of 1 mg/kg and 4 mg/kg achieved the time to maximum concentration (Tmax) of bicuculline concentration in the brain at 10- and 30-min post-injection in rats, respectively. The occurrence of convulsions was detected from immediateness until 110 min after drug injection, consistently with the pharmacokinetics of bicuculline 31. Therefore, the behavioral tests and the MEP recording carried out every week in this study were far beyond the response time course of bicuculline. Accordingly, we did not observe abated behavioral scores (Figure 3D) or cortical MEP signals (Figure 3E-F) of +PBS+bic group compared to that of the vehicle-treated group, suggesting the bicuculine administration and corresponding monitoring methods used in our study did not cause motor disturbances in rats with sub-SCI. However, the therapeutic effect of hUC-MSCs was attenuated in the presence of bicuculline (Figure 3). Therefore, the compromised MSC-mediated functional repair caused by the concomitant treatment of bicuculline may not be the consequence of bicuculline-induced seizures and/or spasticity in the hindlimbs but the specific inhibition on GABA_A_Rs.

Cl⁻ is one of the important anions in the mammalian CNS, and neuronal Cl⁻ homeostasis is crucial for the essential process involved in both neuronal signaling and cell survival 12, 32. The adequate regulation of [Cl⁻]_i_ is tightly associated with the accurate neuronal E/I balance, which is critical for precise descending propagation of the brain command signal. Thus, dysregulation of intracellular Cl⁻ interfering with neuronal excitability and neurotransmission is implicated in several neurological and psychiatric diseases. Given the great importance of both GABA_A_Rs and KCC2 in maintaining the E/I balance, several neurological and psychiatric disorders are associated with their deficiency, resulting in hyperexcitability of neuronal networks, such as epilepsy, neuropathic pain, posttraumatic spasticity, Huntington disease, schizophrenia and Rett syndrome 33-37. Maintenance of the E/I balance was achieved, either by pharmacologically targeting the activation of GABA_A_Rs, which could downregulate motoneuron hyperexcitability and diminish spasticity; or by rescuing KCC2 downregulation *via* NKCC1 antagonists, KCC2 enhancers, KCC2 expression and exercise 18, 38-41, with few studies concentrating on the effect of coordination between GABA_A_Rs and KCC2. Our results revealed that the specific overexpression of GABA_A_R subunit β3 or γ2 in the spinal neurons can favor locomotor network improvement to a similar extent as KCC2 expression, especially in combination with KCC2, facilitated the functional recovery of SCI, suggesting the restoration of the Cl⁻ homeostasis and GABAergic inhibition in neurons may play a pivotal role on neuronal signaling and cell survival across the spinal network after SCI. Functional recovery mediated by hUC-MSCs in SCI could at least be partially accounted for either eliminating the excitotoxic death of neurons or improving the impaired neurotransmission. However, the specific type of targeting neurons has not been elucidated in recent study due to the lack of transgenic rats. To figure out the types of propriospinal neurons and their inhibition responsible for hUC-MSC-mediated repair, we plan to overexpress GABA_A_R subunits and KCC2 in Vglut2-Cre, Vgat-Cre and Chat-Cre transgenic mice, which restrict the expression in excitatory neurons, inhibitory neurons, and motor neurons, respectively. Another unsolved issue in this study is the reason for that GABA_A_R subunits β3 and γ2 rather than α1, α3 or α5 improved the functional recovery of SCI, despite the hUC-MSC transplantation exhibits the upregulation of all those subunits. The differences in subunit composition determine the pharmacological and biophysical properties of the GABA_A_Rs. Structural studies have identified that β3 locates at the β+/α- interface of GABA binding site, whereas γ2 at the α+/γ- interface of benzodiazepine binding site 42. Whether the subunit positioning contributes to the integrity of the receptor remains unclarified. It has been reported that the β3 subunit plays important roles in maintaining inhibitory tone in β1-β3 triple knockout neurons 43. The γ2 subunit, which contributes to receptors involved in both phasic and tonic inhibition, is required for receptor clustering and maintenance 44-46, and interacting with GABA receptor associated proteins 47. Further dissections of how the specific subunit perturbs GABAergic inhibition are guaranteed in our future study.

**Figure 7:**
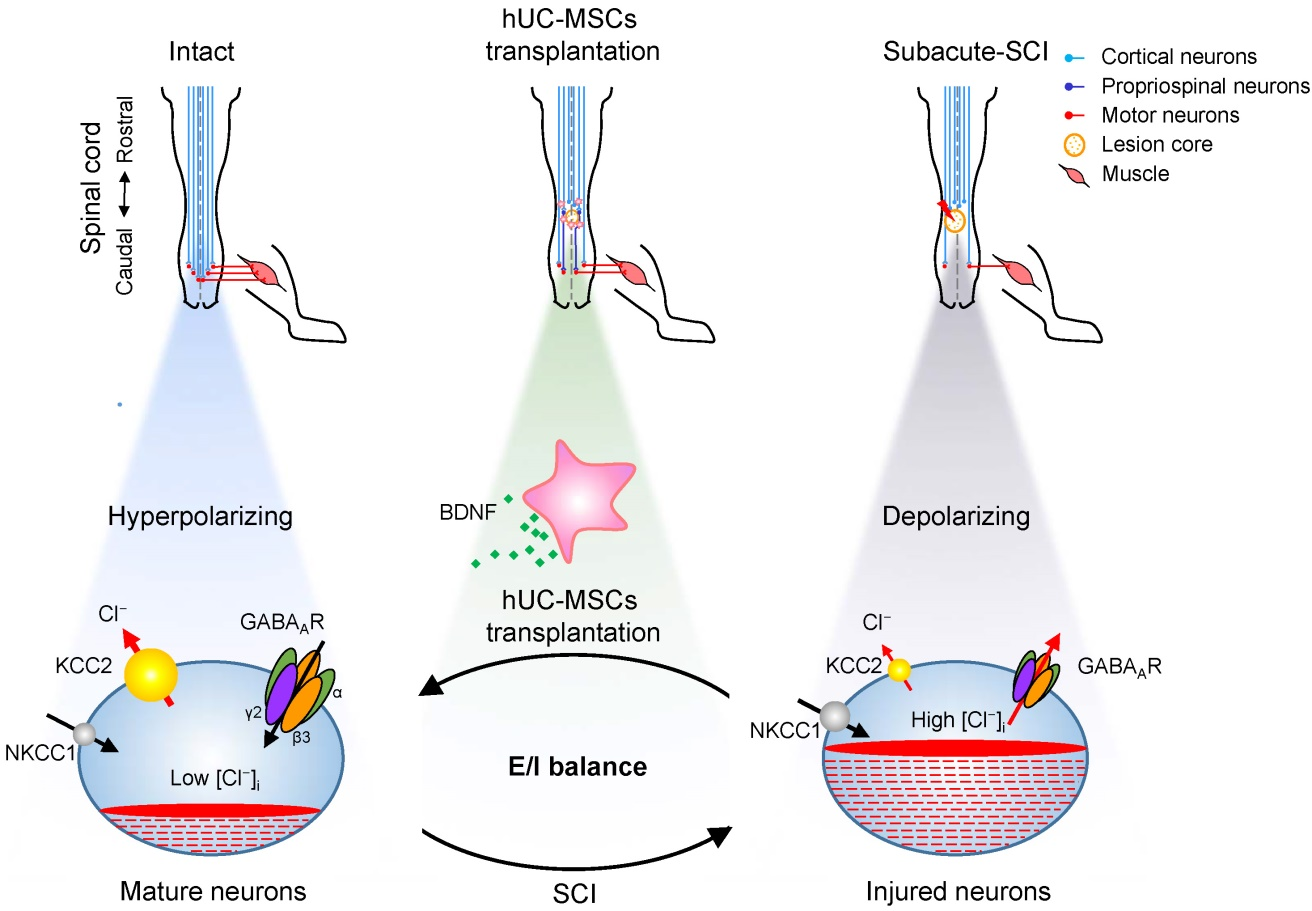
**Proposed model of hUC-MSC-mediated SCI repair through GABA_A_R- KCC2 coordination.** In the intact adult spinal cord, the low neuronal [Cl⁻]_i_ is achieved by the high expression of KCC2 and low expression of NKCC1 which enables the hyperpolarizing action of GABA through activation of GABA_A_Rs. The excitation/ inhibition (E/I) balance ensures the accurate propagation of descending inputs from the brain to hindlimb muscles through the spinal cord. SCI disrupts the E/I balance of the neuronal circuit. The decreased KCC2 in injured neurons causes developmental stage-like high neuronal [Cl⁻]_i_, resulting in an inhibitory-to-excitatory action of GABA. The integration of descending inputs into spinal circuits is compromised by the limited capacity of neuronal regeneration, resulting in hindlimb paralysis. hUC-MSC transplantation increases the expression of both GABA_A_Rs and KCC2 by BNDF secretion that promotes restoration of the E/I balance and/or neuronal survival and restores the perturbed spinal circuits to a more normal state.

Our results revealed a novel mode of hUC-MSC transplantation that specifically targeting the reactivation of GABA_A_R subunits β3 and γ2 in combination with KCC2 in spinal neurons to facilitate the functional recovery of SCI (Figure 7). SCI downregulates the KCC2 expression, leading to an increase in [Cl⁻]_i_, which could restore an immature neuron state with the functional transition of GABA_A_R activation from hyperpolarization to depolarization 48. Thereafter, this depolarization activates Ca^2+^ influx through voltage-gated calcium channels (VGCC) and induces BDNF, which in turn increases the expression and cell surface presence of GABA_A_Rs *via* the transcriptional and posttranslational regulations 49, 50. Although BDNF downregulates the KCC2 expression in healthy mature neurons, it upregulates KCC2 in injured neurons through increasing the GABA_A_-mediated [Ca^2+^]_i_ 51. Thus, the injured CNS tends to transit from the initial developmental state after injury to a physiologically mature state to reestablish the E/I balance and the relay circuit partially through BDNF. In light of this, transplantation of hUC-MSCs provides the injured spinal cord an extra supply of BDNF 25, 52, 53, which along with endogenous BDNF promote the restoration of GABA_A_Rs and KCC2. Therefore, our findings not only unravel the underlying mechanism of hUC-MSC therapy, but also put forward the possibility of pharmacologically targeting the specific GABA_A_R subunit for SCI treatment.

## Materials and Methods

### Ethics statement

Animal experiments were conducted in strict accordance with protocols and guidelines approved by the Institutional Animal Care and Use Committee of Southern Medical University (K2018018). The use of hUC-MSC was approved by the Stem Cell Clinical Trials Ethics Committee of the Third Affiliated Hospital of Sun Yat-sen University. All procedures using primary human cells were under the supervision of Cell-gene Therapy Translational Medicine Research Center in the Third Affiliated Hospital of Sun Yat-sen University.

### Plasmid construction

A double-stranded DNA fragment containing 3×Flag and 3×HA tags was synthesized commercially. 3×HA sequence was RCR amplified using the following primer set, 3×HA-Fwd: 5'- GAGCGCAGTCGAGAAGGTACCCCATGGGAATTCCTCGAGACTAGTTC-3' and 3×HA-Rev: 5'-AGAGGTTGATTATCGATAAGCTTTTAGGACCCAGCGTAATCTGGAA-3' and recombined into linearized pAAV-hSyn1-mRuby2 (Addgene, #99126) with KpnI/HindIII double-digestion to generate pAAV-hSyn1-3×HA. The same strategy was applied to replace mRuby2 with 3×Flag to generate pAAV-hSyn1-3×Flag, using the following primers, 3×Flag-Fwd: 5'-GAGCGCAGTCGAGAAGGTACCGAATTCGGATCCCCATGGGACTACAAGGACCATGACGG-3' and 3×Flag-Rev: 5'-AGGTTGATTATCGATAAGCTTtcaCTTGTCATCGTCGTCCTTGT-3'. Rat Gabra1, Gabra3, Gabra5, Gabrb3, Gabrg2 and Kcc2 cDNAs were cloned from the rat spinal cord. Cloning primers were listed in Table S4. The pAAV-hSyn1-Gabra1-3×HA, pAAV-hSyn1-Gabra3-3×HA, pAAV-hSyn1-Gabra5-3×HA, pAAV-hSyn1-Gabrb3-3×HA and pAAV-hSyn1-Gabrg2-3×HA vectors were generated by insertion of each cDNA into pAAV-hSyn1-3×HA linearized by KpnI/BamHI, respectively. Whereas Kcc2 was cloned into pAAV-hSyn1-3×Flag, with tag fusing to its C-terminal.

### Cell culture

The hUC-MSCs isolated from umbilical cords 54 were maintained in growth medium (DMEM (Corning, USA) containing 10% FBS, 1% glutamine, 1% MEM-NEAA, 20 ng/ml recombinant human bFGF (Genescript, Z02734-1) and 1× penicillin/streptomycin (Corning, 30-002-CI-100ml)) and passaged every 2 days. The characteristics of each cell batch were qualified before intrathecal transplantation. Details were provided in SI Materials and Methods. The passage number of hUC-MSCs used for transplantation in this study was lower than 5.

### Rat models of the subacute incomplete thoracic SCI and intrathecal hUC-MSC transplantation

250 g adult female Sprague-Dawley rats at the age of ~2 months were used in this study. The rat models of subacute incomplete SCI at the thoracic level were constructed and hUC-MSCs were administered intrathecally as previously described 7, 55. Briefly, the rats were allowed to acclimatize to the housing facility for at least 3 days before the experiment. All the animals were randomly grouped and intraperitoneally anesthetized with 1% pentobarbital sodium (25 mg/kg, Fluka, USA). A T9-T11 laminectomy was performed to expose the spinal cord at the thoracic level. The compression injury in the rat spinal cord was generated by an aneurysm clip (Sugita, Japan) with the clamping force of 60 *g* at T10 for 1 min. After surgery, animals received intramuscular injection of penicillin (50,000 unit/kg/day; Jusheng, PRC) for 3 days. Manual micturition and nursing care were applied twice daily to prevent associated complications. hUC-MSCs transplantation was performed at day 7 post injury (sub-SCI). Intrathecal administration of either 1×10^6^ cells/kg of hUC-MSCs (+MSC) or PBS (+PBS) at the lumbar spinal cord was performed using a micro-injection pump (R404, RWD, PRC). To suppress immunological rejection, rats were subcutaneously administered with cyclosporin A (10 mg/kg, Cyonse, PRC) daily over the entire experimental period. In this study, all rodents were routinely followed up for 30 days after intrathecal transplantation.

### Behavioral experiments

Locomotor function was evaluated weekly by two experienced examiners in a blinded fashion using a series of behavioral tests, including the Basso, Beattie and Bresnahan (BBB) scale, beam walk and Rivlin test, which were detailed previously. For all 3 measurements, we carried out three biological replicates for all groups (n = 4/group/replicate).

### Motor evoked potential (MEP) recording

MEP recording was performed as previously described. In brief, 6 rats from each group were randomly selected at the endpoint of the experiment (n = 2/group/replicate). Anesthetized animals were fixed in a stereotaxic apparatus and their motor cortices were exposed. Stimulating electrode was placed in the representative hindlimb area of the right motor cortex, and the recording electrode was inserted into the gastrocnemius muscle of the contralateral hindlimbs. MEP signals were acquired using the BL-420 A/F Data Acquisition Analysis System (TAIMENG SOFTWARE, Chengdu, China) with the electrical stimulation parameters as follows, 10 V, 1 ms pulse width, 50 ms and 10 Hz frequency. The amplitude (mV) and latency (ms) were obtained.

### RNA-Seq and data analysis

5-mm spinal cord segments centered at the epicenter from rats with different treatments were collected for total RNA extraction. Total RNAs were sent to Novogene for library construction and high-throughput sequencing. Briefly, sequencing libraries were generated using the NEBNext^®^ UltraTM RNA Library Prep Kit for Illumina^®^ (NEB, USA) from RNAs with poly (A) tails that purified by poly-T oligo-attached magnetic beads. The library quality was assessed on the Agilent Bioanalyzer 2100 system. Libraries were sequenced on Illumina Hiseq platform to generate totally at least 8 G clean data/library with 150 bp paired-end reads. We sequenced total 26 libraries, including three biological replicates for each condition, besides four repeats for Intact, +MSC-4w and +PBS-4w groups, and two repeats for +MSC-2w group. Clean data were obtained by using the Trim Galore (v0.5.0) to remove reads containing adapter, ploy-N and low-quality reads (Q < 30). The remaining reads were then mapped to reference genome using the Hisat2 (v2.1.0). To filter out the reads that might generate from the residual hUC-MSCs in the rat spinal cord, the clean reads were firstly mapped to the human reference genome (GRCh38). Then, the filtered non-human reads were further aligned to the rat reference genome (Rnor6) to obtain the data generated from the rat spinal cord. Using the Samtools (v1.9) with the parameter -f 2, reads mapped in the proper pair were extracted. For downstream analyses, the reads number and TPM (Transcripts Per Million) value for each gene were calculated by featureCounts (v1.6.4) and StringTie (v1.3.5), respectively.

### Differential gene expression analysis

RNA-seq differential expression analysis was performed using the DESeq2 (v1.24.0) R package. Transcripts with |log_2_FC| > 1 and adjusted *p*-value < 0.01 were considered as differentially expressed genes (DEGs). DEGs were identified based on three contrasts, sub-SCI *vs*. Intact, +PBS *vs*. Intact, and +MSC *vs*. +PBS at different time points.

### Gene Ontology (GO) enrichment analysis and Kyoto Encyclopedia of Genes and Genomes (KEGG) pathway analysis

GO and KEGG analyses of DEGs were conducted using the clusterProfiler (v3.12.0) R package. GO terms, including the biological process (BP), molecular function (MF), and cellular component (CC) were investigated. The adjusted *p*-value < 0.05 was considered significantly enriched.

### Sample clustering

The top 5000 DEGs in DEGs List as mentioned above, were used to perform the Z-Score normalization. Then, we used the ComplexHeatmap (v2.0.0) 56 to draw the heatmap with the ward.D cluster method.

### Gene Set Enrichment Analysis (GSEA)

GSEA was performed using the GSEA software (v2.07) 57. The gene expression profiles of neurons, astrocytes, myelinating oligodendrocytes (MOs), microglia and endothelial cells were retrieved from GSE52564. Both MSC-repressed genes and MSC-activated genes were tested. We permutated the gene set for 1000 times rather than permutating the phenotype as the sample number is small.

### Venn diagram

hUC-MSC-activated and -repressed genes were used to overlap with consensus signature modules derived from Squair et al. 13. Venn diagrams were generated by ggplot2 (v3.2.1) and the *p* value was calculated by hypergeometric test.

### RT and quantitative real-time PCR

Briefly, total RNA from spinal cord segments was extracted with the TRIzol^®^ Reagent (Thermo fisher, 15596018). 1 ug of total RNA was converted into cDNA using the reverse transcription system (Thermo fisher, 4311235). Real-time PCR reactions were performed in triplicates using the SYBR Green PCR Master Mix (Yeasen, 11203ES08) on the StepOnePlus™ system (Applied Biosystems). The internal control was 28S rRNA. Real-time PCR primers used in this study were listed in the Table S4.

### Western blotting

Western blot analysis was performed as described before 58. Spinal cord segments were lysed in RIPA buffer (150 mM NaCl, 50 mM Tris-HCl pH 8.0, 5 mM EDTA, 1% Triton X-100, 0.1% SDS, 1% sodium deoxycholate, 1 mM PMSF, 5 mg/mL leupeptin (Roche, 11034626001), 5 mg/mL pepstatin (Roche, 11524488001), and 10 mg/mL aprotinin (Roche, 11583794001)). 50-100 μg of total proteins was separated on an SDS-PAGE gel and transferred onto nitrocellulose (NC) blotting membranes (GE, 1060001). The membrane was blocked in PBST (1× PBS with 0.05% Tween 20) plus 5% milk for 1 h at room temperature. After blocking, the membrane was incubated with primary antibodies, and then with the horseradish peroxidase (HRP)-coupled secondary antibody, and signals were developed using Western Lightning Plus ECL (Perkinelmer, NEL105001EA).

### Immunofluorescence (IF) staining and confocal microscopy

At the endpoint of the experiment, whole spinal cords were rapidly removed from the anesthetized rats and placed into ice-cold saline. The ~3-cm spinal cord centered at the T10 injury site was evenly divided into three segments, 1-cm tissue centered on the injury epicenter, 1-cm tissue rostral and 1-cm tissue caudal to the epicenter. Sections (20-μm thick) were cut and stained with primary antibodies overnight followed by incubation with the secondary antibody. Antibodies used for Immunofluorescence staining were listed in Table S3. Cell nuclei were stained with DAPI (Sigma, D9542-10MG). Transverse sections of spinal cords were photographed on the Zeiss AXIO scope A1 for both low (2.5x) and high magnification (20x) images. ImageJ software (Version 1.8.0) was used to quantify fluorescence intensity of different proteins.

### Bicuculline treatment

Rats with the subacute SCI were randomly assigned into four treatment groups, +PBS, +PBS+bicuculline, +MSC, and +MSC+bicuculline. Animals received either a control vehicle solution (sesame oil + 10% DMSO) or bicuculline (3.5 mg/kg; MCE, HY-N0219) combined without or with hUC-MSC transplantation. The day after hUC-MSC administration, all drugs were administered *via* daily subcutaneous injections in the scruff of the neck over the entire experimental period. Behavioral tests and MEP recording every week were carried out prior to the daily administration of bicuculline.

### Adeno-associated virus (AAV) production and intrathecal injection

The production of AAV was performed according to the protocol developed by Chillis et al. 21. Briefly, 293t cells were transfected with 3 plasmids, i.e., pAAV, pUCmini-iCAP-PHP.eB (Addgene, #103005) and pHelper (ZoomaBio, ZK736), at the ratio of 1:4:2. We transfected 40 µg of total DNA (5.7 µg of pAAV, 22.8 µg of pUCmini-iCAP-PHP.eB, and 11.4 µg of pHelper) into 293t cells in a 150-mm dish. The medium was changed 12 h after transfection. The medium containing AAV was collected at 72 h and 120 h post-transfection, respectively, and the cells were collected as well at 120 h post-transfection. AAV particles were released from cells by Salt-active nuclease (SAN; ArcticZymes, 70910-202) digestion and precipitated with particles in the supernatant medium together using PEG8000 (Sigma, 89510-1KG-F). AAV particles were further purified by ultracentrifugation in the iodixanol density gradient at 350,000 g for 2 h at 18 °C. Amicon Ultra-15 centrifugal filter devices (Millipore, UFC910024) were used to concentrate the virus in 500-ul DPBS. The virus titer was determined by real-time PCR using the following primers, WPRE-forward: 5'-GGCTGTTGGGCACTGACAAT-3'; WPRE-reverse: 5'-CCGAAGGGACGTAGCAGAAG-3'; hGH polyA-forward: 5'-GTGCCCACCAGCCTTGTC-3'; hGH polyA-reverse: 5'-TGTCTTCCCAACTTGCCCCTT-3'. The optimal dose for transducing spinal cord neurons of adult rats through intrathecal AAV administration was determined before overexpression (data not shown). The intrathecal viral injections of 5×10^12^ vg AAV particles in 40-ul DPBS were applied to the spinal cord of rats (200-250 g) with the subacute SCI at L5-L6 level by using a micro-injection pump (RWD, R404) at the rate of 6 μl/min.

### Lentivirus-based BDNF knockdown and overexpression in hUC-MSCs

Lentivirus was generated according to our previous protocol 58. Briefly, shRNAs targeting luciferase and human BDNF were cloned into the pLKO.1 vector (Addgene, #8453). The coding sequence of rat BDNF was cloned into lentiviral expression vector pLVX-IRES-puro (632183, Clontech) under the CMV promoter to generate pLVX-BDNF, and the empty vector (EV) was used as a negative control. The sequences of the shRNAs and primers for cloning BDNF from the rat brain were listed in Table S4. The lentiviral vectors were co-transfected with the packaging vectors, pCMV-deltaR8 (Addgene, #12263) and pCMV-VsVg (Addgene, #8454) into LentiX-293T cells to generate virus. Culture medium containing viruses was collected at 48 h and 72 h after the transfection, respectively. Transduced hUC-MSCs were selected with 2 μg/ml puromycin (Sigma) for 2 days. The knockdown and overexpression efficiency were validated by real-time PCR and Western blotting.

### Statistical analysis

Data are presented as mean ± SEM. One-way or Two-way ANOVA was performed to determine statistical significance between groups in experiments. For all analyses, significance was defined as † or *p < 0.05; †† or **p < 0.01; ††† or ***p < 0.001; *††††* or ****p < 0.0001; n.s.^#^ or n.s., not significant.

**Accession numbers.** The accession number for the RNA-seq data reported in this paper is GEO: GSE178564.

## Supplementary Material

Supplementary methods, figures and tables.

## Abbreviations

A: astrocytes
AAV: adeno-associated virus
BBB: basso, beattie and bresnahan
BDNF: brain-derived neurotrophic factor
BFGF: human basic fibroblast growth factor
Bic: bicuculline
BP: biological process
CC: cellular component
CCCs: cation-chloride cotransporters
CMV: cytomegalovirus
CNS: central nervous system
DEGs: differentially expressed genes
DIV: days in vitro
DMEM: dulbecco's modified eagle's medium
DMSO: dimethylsulfoxide
EC: endothelial cells
EDTA: ethylenediaminetetraacetic acid
E/I: excitation/inhibition
EV: empty vector
FBS: fetal bovine serum
GABA_A_R: γ-aminobutyric acid type A receptor
GAPDH: glyceraldehyde-3-phosphate dehydrogenase
GO: gene ontology
GSEA: gene set enrichment analysis
HRP: horseradish peroxidase
hSyn1: human synapsin 1
hUC-MSCs: human umbilical cord-derived mesenchymal stem cells
IF: immunofluorescence
KCC2: K^+^/Cl^-^ cotransporter 2
KEGG: Kyoto encyclopedia of genes and genomes
MAP2: microtubule-associated protein-2
MEM-NEAA: minimum essential medium-nonessential amino acid
MEPs: motor evoked potentials
MF: molecular function
MG: microglia
MOs: myelinating oligodendrocytes
nc: nitrocellulose
N: neurons
NeuN: neuronal nucleus
NKCC1: Na^+^-K^+^-2Cl⁻ cotransporter 1
PBS: phosphate buffered saline
PCA: principal component analysis
PCR: polymerase chain reaction
PMSF: phenylmethanesulfonyl fluoride
rhBDNF: recombinant human brain-derived neurotrophic factor
RIPA: radio immunoprecipitation assay
RT-qPCR: reverse transcription followed by quantitative polymerase chain reaction
SCI: spinal cord injury
SDS: sodium dodecyl sulfate
TMAX: time to maximum concentration
Tris-HCl: tris(hydroxymethyl) aminomethane-hydrogen chloride
VGCC: voltage-gated calcium channels
wpt: weeks post-transplantation
