# Supplementary material for: hUC-MSC-mediated recovery of subacute spinal cord injury through enhancing the pivotal subunits β3 and γ2 of the GABAA receptor
Source: Theranostics. 2022 Mar 28;12(7):3057–78. doi: 10.7150/thno.72015 (PMC9065192; doi:10.7150/thno.72015)
Supplement: Supplementary file 1 — Supplementary methods, figures and tables. [file thnov12p3057s1.pdf]

Supplemental Information

hUC-MSC-mediated recovery of subacute spinal cord injury through enhancing the pivotal subunits β3 and γ2 of the $GABA_A$ receptor

Tingting Cao [a, 1], Huan Chen [a, 1], Weiping Huang [a, 1], Sisi Xu [a], Peilin Liu [a], Weiwei Zou [a], Mao Pang [b, c, d], Ying Xu [b, c, d], Xiaochun Bai [a], Bin Liu [b, c, d], Limin Rong [b, c, d], Zhong-Kai Cui [a, *], Mangmang Li [a, *]

a. Department of Cell Biology, School of Basic Medical Sciences, Southern Medical University, Guangzhou, 510515, China.

b. Department of Spine Surgery, The Third Affiliated Hospital of Sun Yat-sen University, Guangzhou, China

c. Guangdong Provincial Center for Engineering and Technology Research of Minimally Invasive Spine Surgery, 510630, China

d. Guangdong Provincial Center for Quality Control of Minimally Invasive Spine Surgery, 510630, China

[1]These authors contributed equally to this work.

[*]To whom correspondence should be addressed. E-mail: zhongkaicui@smu.edu.cn, mangmangli@smu.edu.cn

Supplemental Experimental Procedures

Isolation and expansion of hUC-MSCs

Briefly, umbilical cord tissues collected within 2 h were disinfected with 75% ethanol and washed with 0.9% sodium chloride. Two arteries and one vein within the umbilical cord were dissected with surrounding Wharton's Jelly and mechanically cut into 1-cm^3 fragments, then digested in a mixed enzyme solution consisting of 10 mg/ml type I collagenase (Thermo fisher, 17100017) and 1.25 mg/ml hyaluronidase (Sigma, H3506-500mg) for ~3 h. Undigested tissue was removed by filtration and the released cells were washed twice with PBS and cultured in the hUC-MSC growth medium (low-glucose DMEM (Corning, 10-014-CV) containing 10% FBS, 1% glutamine (Corning, 25-005-CI), 1% MEM Nonessential Amino Acids (Thermo fisher, 11140050) and 1x penicillin/streptomycin (Corning, 30-002-CI-100ml)). Once the culture attained 80% confluency, the outgrown cells were passaged three times to remove unwanted cells and obtain the highest purity fraction for further study.

Human cell quantification

The number of survived hUC-MSCs transplanted in rat spinal cord was quantified through amplifying human-specific gene *Xrcc5* in genomic DNA from the rat spinal cord using quantitative real-time PCR (qPCR). Basically, certain amount of spinal cord specimens and/or cells were lysed in the house-made DNA extraction buffer (1 M Tris-HCl (pH 8.0), 0.5 M EDTA, 10% SDS, 5 M NaCl and 10 mg/ml proteinase K in ddH_2O) by agitating at 55°C overnight. DNA was precipitated and dissolved in 10 mM Tris-HCl. DNA concentrations were measured using the NanoDrop-1000 (Thermo Fisher Scientific, Inc., USA). qPCR was performed to quantify the level of *Xrcc5* from 100 ng of genomic DNA in a 10-μl PCR reaction volume on the StepOnePlus™system (Applied Biosystems-Thermo Fisher Scientific, USA). Serial dilutions of human

genomic DNA from hUC-MSCs (1000, 100, 10 and 1 cell) mixed with genomic DNA from plain rat spinal cord were used as the standard curve, and the theoretical number of hUC-MSCs in rat spinal cord was calculated accordingly.

Cell-labeling with DID or DiR and *in vivo* imaging

hUC-MSCs were labeled using lipophilic tracers DiD (aatbio, #22033) or DiR (aatbio, #22070) which are well excited at 633 nm and 750 nm, respectively. Briefly, 1×10^6 cells/ml were incubated with 5 μM of DID or DiR working solution for 20 min at 37 °C. The labeled cells were washed twice before resuspending in 40ul of PBS for intrathecal injection using a micro-injection pump (R404, RWD, PRC). 5cm spinal cord tissue (from 1cm rostral to 4 cm caudal from lesion epicenter) was harvested at day 10 (d10) and day 20 (d20) post-transplantation and fluorescent images of spine transplanted with or without labeled MSCs were acquired using In vivo Imaging System FX Pro (Bruker, MIAB1339627).

Principal component analysis (PCA)

The top 1000 DEGs with the most differences (Sort by standard deviation value between samples) were used to perform PCA. Plot3D (v1.3) R package was used to plot the figure.

Axon regeneration of mature cortical neuron *in vitro*

Primary cultures of rat cortical neurons and mature cortical axon scrape assay were performed as described previously [1]. Briefly, brains were removed from E18 Sprague-Dawley rat fetuses and cortical tissues were dissected and minced into small pieces. Chunks of cortex were dissociated in the dissection medium (DMEM (Corning, 10-013) supplemented with the B-27™ Supplement (Thermo Fisher, 17504044), 1 mM sodium pyruvate (Solarbio, sp0100), and 10 μg/ml gentamicin (Macklin, G810322-25g)) containing 4.2 mg/ml papain (KEH, 9001-73-4) and 0.02 mg/ml DNase I (Roche,

4716728001) for 1 h at 37 °C under gentle shaking. After enzyme digestion, cells were further dispersed by mechanical trituration and filtered through a 70-μm cell strainer. Cells were resuspended in the Neurobasal (Gibco, 21103-049) supplemented with the B-27, 0.5 mM GultaMAX-I (Thermo Fisher, 35050061), and 10 μg/ml gentamicin. 1.6×10^5 cells/well were plated in 24-well culture plates coated with 10 μg/ml poly-D-lysine (Sigma, P6407-5MG). 50% of culture medium was replaced every 7 days. On day 21 *in vitro* (DIV), 24-well cultures were scraped using the tip of a 10-μl pipette tip. Co-culture with hUC-MSCs was started 12 h post injury. Neurons were co-cultured with 0.8×10^4 hUC-MSCs, plated in the 24-well trans-well upper chamber with a 0.4-μm filter membrane (Corning, 353095) for 7 days. 50 ng/ml recombinant human BDNF (rhBDNF; Genscript, Z03208) was used as positive control. Cultures were then fixed and immunostained with different primary and secondary antibodies (Table S3). Images were obtained on an Olympus Fluoview FV1000 inverted fluorescence microscope. The immunofluorescence intensity of each protein was determined using the ImageJ software.

Reference

1. Huebner EA, Kim BG, Duffy PJ, Brown RH, Strittmatter SM. A multi-domain fragment of Nogo-A protein is a potent inhibitor of cortical axon regeneration via Nogo receptor 1. J Biol Chem. 2011; 286: 18026-36.

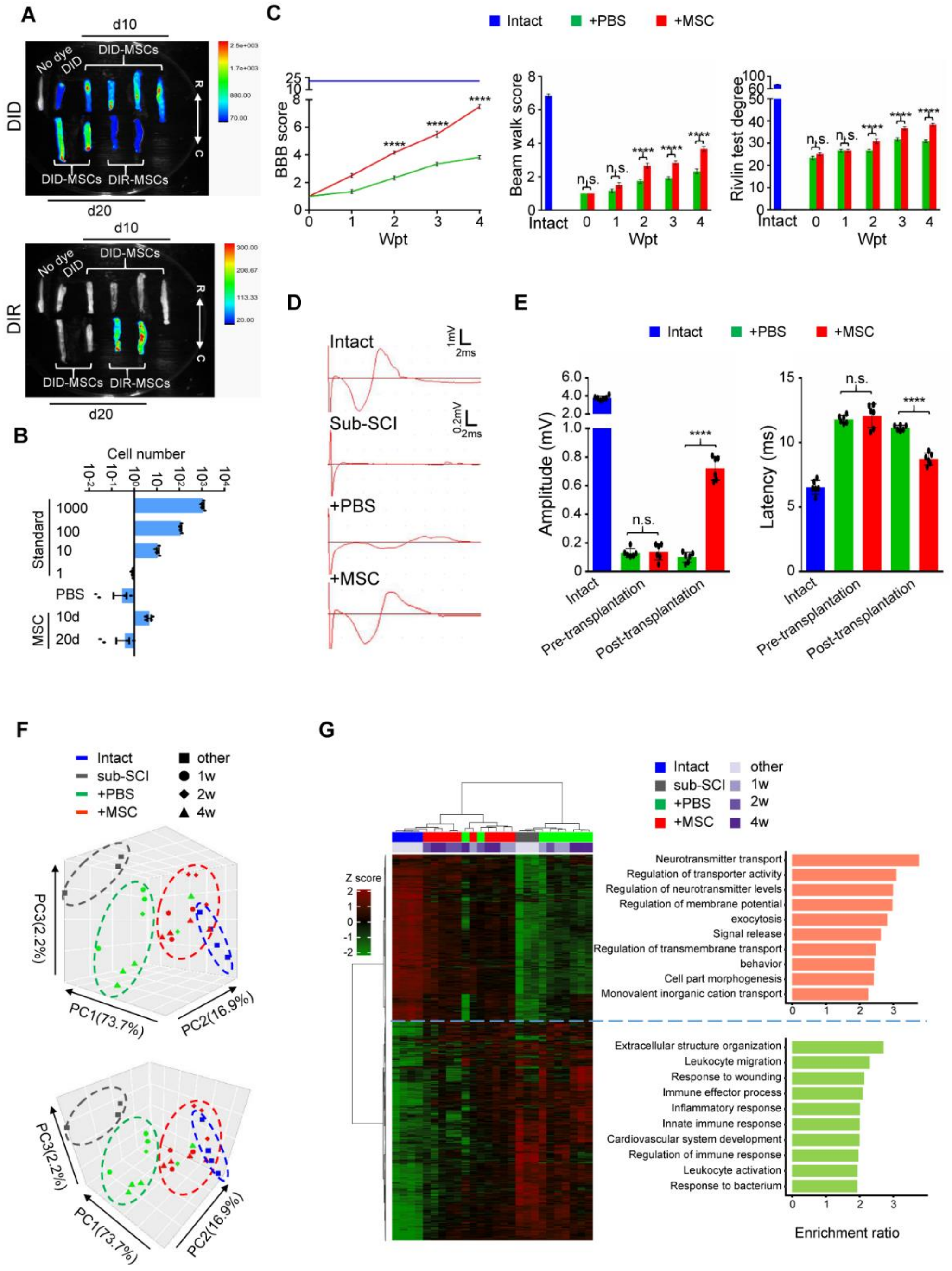

Figure S1 hUC-MSC transplantation exhibits functional recovery in the rat model of subacute SCI. (A) Representative fluorescence images of 5-cm rat spinal cords dissected after intrathecally injected with hUC-MSCs labeled with DiD (top) or DIR (bottom) at day 10 (d10) and day 20 (d20) post-transplantation. The spinal cords injected with PBS or DID working solution were used as negative controls. R, rostral;

C, caudal. (B) Real-time qPCR to quantify the number of survived hUC-MSCs in the rat spinal cords at d10 and d20 post-transplantation (n = 4). Data are presented as mean ± SEM. (C) A series of behavioral tests to evaluate the effects of hUC-MSCs on the functional recovery after SCI (n = 12/group/time point). The color key is used for C and E. Data are presented as mean ± SEM. n.s., not significant; ****$p < 0.0001$ by two-way ANOVA. (D) Representative MEP recordings of rats treated with or without hUC-MSCs at 4 wpt (n = 6/group). (E) Quantification and statistical analyses of D. Data are presented as mean ± SEM. n.s., not significant; ****$p < 0.0001$ by one-way ANOVA. (F) PCA of all samples. (G) Heat map of hierarchical clustering of all 26 samples based on the top 5000 DEGs.

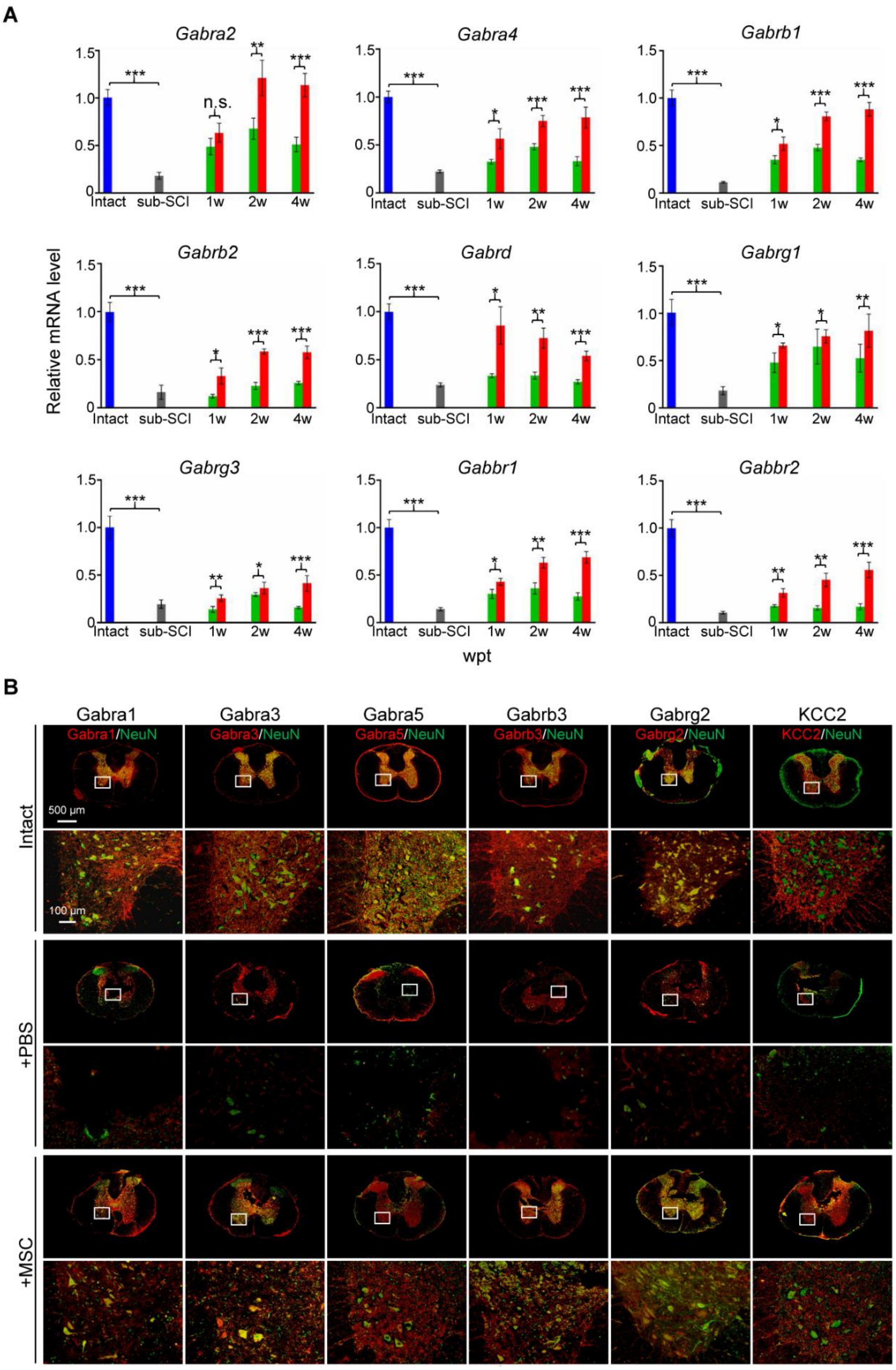

Figure S2 hUC-MSC transplantation increases GABA$_A$Rs in the injured spinal cord. (A) Time course of real-time PCR validation of hUC-MSC-activated GABA

receptor subunits in rat model of subacute SCI treated with or without hUC-MSCs (n = 3). Results are displayed as mean ± SEM. *$p < 0.05$; **$p < 0.01$; ***$p < 0.001$ by two-way ANOVA. (B) Representative IF images of transverse spinal cord sections from the Intact, -MSC and +MSC groups at 4 wpt immunostained with Gabra1, Gabra3, Gabra5, Gabrb3, Gabrg2, KCC2 (red) and NeuN (green). Lower panels, higher magnification view of the region indicated by the white rectangle in main panels. Scale bar, 500 μm (main panels), 100 μm (lower panels).

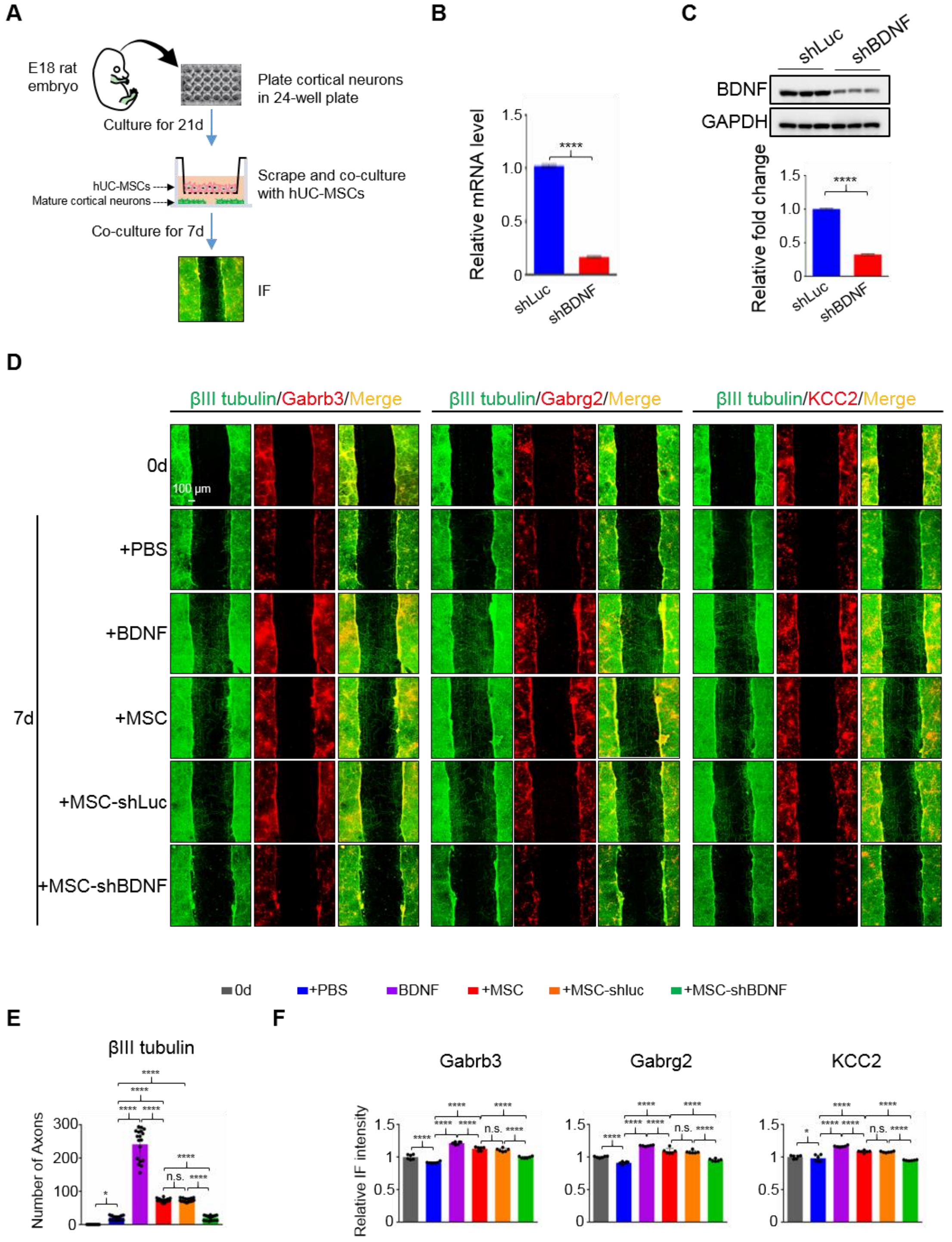

Figure S3 BDNF knockdown abrogates the effects of hUC-MSCs on axonal regeneration and the induction of GABA$_A$Rs in neurons after axonal lesion *in vitro*.

(A) Schematic for hUC-MSC-mediated mature cortical axon regeneration. (B) Real time-PCR to measure BDNF knockdown in hUC-MSCs (n = 3/group). Luc, Luciferase.

(C) Western blot analysis of BDNF in hUC-MSCs transduced with the lentiviral shluc and shBDNF (n = 3/group). In B and C, data are presented as mean ± SEM. ****$p < 0.0001$ by unpaired 2-tailed Student's t test. (D) Representative IF staining of regenerated axons of mature cortical neurons at day 0 and 7 after axotomy with indicated treatments, *i.e.*, 50 ng/ml rhBDNF, without or with hUC-MSCs, shluc and shBDNF transduced hUC-MSCs. Neurons were co-stained with anti-βIII tubulin (green), and anti-Gabrb3 (red), Gabrg2 (red), and KCC2 (red) antibodies. Scratch and non-scratch zones are denoted by the curly braces. Scale bars, 100 μm. (E) Quantification of βIII tubulin-positive regenerating axons in the scratched zone in D (n = 18). (F) Quantification of immunofluorescence intensities for Gabrb3, Gabrg2 and KCC2 in the non-scratched zone in D (n = 6). The color key is used for both E and F. The images were obtained from three independent experiments. The data are presented as mean ± SEM. n.s., not significant; *$p < 0.05$, **$p < 0.01$, ***$p < 0.001$, ****$p < 0.0001$ by one-way ANOVA.

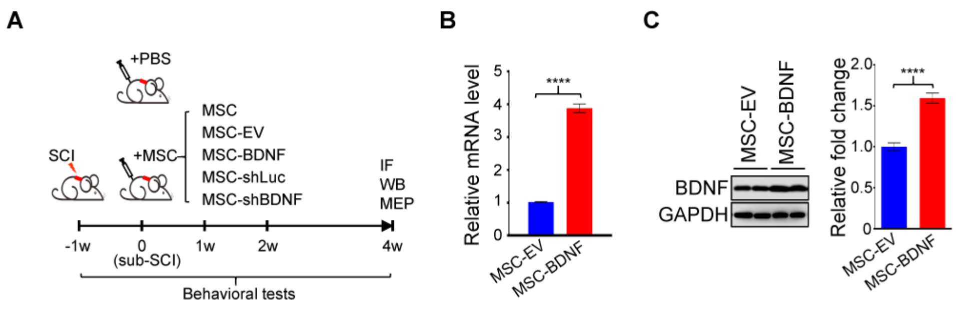

Figure S4 The transplantation of hUC-MSCs with either BDNF overexpression or knockdown into rats with sub-SCI. (A) The experimental scheme for investigating the role of BDNF secreted by hUC-MSCs on SCI repair in the rat models. (B) Real-time qPCR validation of BDNF overexpression in hUC-MSCs (n = 3/group). (C)

Western blotting and quantification analyses of BDNF overexpression in hUC-MSCs (n = 3/group). Results are displayed as mean ± SEM. ****$p < 0.0001$ by one-way ANOVA.

Table S1. GO and KEGG analyses of hUC-MSC-reversed genes.

Repressed genes_GO

ONTOLOGY	ID	Description	GeneRatio	BgRatio	pvalue	p.adjust	qvalue	geneID	Count
BP	GO:0043062	extracellular structure organization	23/260	313/19943	2.4824E-11	2.31558E-08	1.9551E-08	Col13a1/Col1a1/Fap/Emilin1/Postn/Col4a	23
BP	GO:0031589	cell-substrate adhesion	23/260	347/19943	1.9514E-10	1.13769E-07	9.6057E-08	Col13a1/Bst1/Col1a1/Emilin1/Plau/Postn	23
BP	GO:0030198	extracellular matrix organization	20/260	269/19943	4.2256E-10	2.18978E-07	1.8489E-07	Col13a1/Col1a1/Fap/Emilin1/Postn/Col4a	20
BP	GO:0097530	granulocyte migration	14/260	143/19943	5.7516E-09	2.68257E-06	2.2649E-06	Trpv4/Bst1/Lbp/Adam8/Trem1/Ccl6/Spp	14
BP	GO:0097529	myeloid leukocyte migration	16/260	203/19943	1.0842E-08	4.21408E-06	3.558E-06	Trpv4/Bst1/Emilin1/Lbp/Adam8/Trem1/C	16
BP	GO:0010810	regulation of cell-substrate adhesion	16/260	220/19943	3.3824E-08	1.1964E-05	1.0101E-05	Bst1/Col1a1/Emilin1/Plau/Postn/Ninj1/Da	16
BP	GO:0001525	angiogenesis	24/260	494/19943	3.5913E-08	1.1964E-05	1.0101E-05	Angptl4/Emilin1/Plau/Hmox1/Col4a1/Ada	24
BP	GO:0050707	regulation of cytokine secretion	16/260	225/19943	4.6315E-08	1.39073E-05	1.1742E-05	Trpv4/Postn/Htr2b/Tlr8/Srgn/Twist1/Fblr	16
BP	GO:0050663	cytokine secretion	17/260	256/19943	4.7709E-08	1.39073E-05	1.1742E-05	Trpv4/Postn/Htr2b/Trem1/Tlr8/Srgn/Twis	17
BP	GO:0071295	cellular response to vitamin	8/260	44/19943	8.8517E-08	2.4285E-05	2.0504E-05	Col1a1/Postn/Folr2/Pawr/Kank2/Ltbp1/S	8
BP	GO:0042060	wound healing	22/260	444/19943	9.7164E-08	2.51763E-05	2.1257E-05	Col1a1/Plau/Postn/Hmox1/Ninj1/Sdc1/C	22
BP	GO:0007160	cell-matrix adhesion	15/260	209/19943	1.0892E-07	2.67376E-05	2.2575E-05	Col13a1/Bst1/Emilin1/Plau/Postn/Ninj1/N	15
BP	GO:0010876	lipid localization	20/260	378/19943	1.3305E-07	3.02624E-05	2.5551E-05	Trpv4/Slco2a1/RGD1305807/Msr1/Lbp/P	20
BP	GO:0006869	lipid transport	19/260	343/19943	1.3626E-07	3.02624E-05	2.5551E-05	Trpv4/Slco2a1/RGD1305807/Msr1/Lbp/P	19
BP	GO:0038063	collagen-activated tyrosine kinase recep	5/260	11/19943	1.5706E-07	3.32971E-05	2.8113E-05	Col1a1/Col4a1/Col4a2/Col4a5/Col4a6	5
BP	GO:0050710	negative regulation of cytokine secretio	9/260	68/19943	2.3873E-07	4.84101E-05	4.0873E-05	Tlr8/Srgn/Fbln1/Fn1/Lrrc32/Serpinb1a/Ar	9
BP	GO:0050727	regulation of inflammatory response	19/260	358/19943	2.6385E-07	5.12756E-05	4.3293E-05	Trpv4/Bst1/Enpp3/Lbp/Adam8/Il20rb/Vsi	19
BP	GO:1990266	neutrophil migration	11/260	116/19943	3.519E-07	6.56502E-05	5.543E-05	Bst1/Lbp/Adam8/Trem1/Ccl6/Spp1/C5ar	11
BP	GO:0071621	granulocyte chemotaxis	11/260	119/19943	4.5602E-07	7.33401E-05	6.1922E-05	Trpv4/Bst1/Lbp/Trem1/Ccl6/Spp1/C5ar1/	11
BP	GO:0001818	negative regulation of cytokine producti	16/260	267/19943	4.8042E-07	7.46886E-05	6.3061E-05	Hmox1/Lbp/Il20rb/Vsig4/Tlr8/Srgn/Twist	16
BP	GO:0031670	cellular response to nutrient	10/260	98/19943	6.1068E-07	8.74974E-05	7.3876E-05	Col1a1/Postn/Hmox1/Folr2/Ptgs2/Pawr/	10
BP	GO:1903531	negative regulation of secretion by cell	15/260	239/19943	6.1472E-07	8.74974E-05	7.3876E-05	Hmox1/Tlr8/Igfbp3/Srgn/Crh/Fbln1/Fn1/	15
BP	GO:0006721	terpenoid metabolic process	9/260	76/19943	6.3029E-07	8.74974E-05	7.3876E-05	Akr1b10/Adh1/Adh6/Crh/Rbp1/Hmgcs2/	9
BP	GO:0033273	response to vitamin	13/260	178/19943	6.3785E-07	8.74974E-05	7.3876E-05	Col1a1/Postn/Folr2/Spp1/Ptgs2/Pawr/Ka	13
BP	GO:0038065	collagen-activated signaling pathway	5/260	14/19943	6.5911E-07	8.78312E-05	7.4157E-05	Col1a1/Col4a1/Col4a2/Col4a5/Col4a6	5
BP	GO:0071560	cellular response to transforming growt	15/260	246/19943	8.8553E-07	0.000114726	9.6865E-05	Col1a1/Htra3/Emilin1/Ltbp2/Postn/Col4a	15
BP	GO:0071559	response to transforming growth factor	15/260	253/19943	1.2593E-06	0.000154566	0.0001305	Col1a1/Htra3/Emilin1/Ltbp2/Postn/Col4a	15
BP	GO:0016101	diterpenoid metabolic process	8/260	64/19943	1.7653E-06	0.000200817	0.00016955	Akr1b10/Adh1/Adh6/Crh/Rbp1/Rarres2/	8
BP	GO:0051048	negative regulation of secretion	15/260	268/19943	2.5731E-06	0.000272745	0.00023028	Hmox1/Tlr8/Igfbp3/Srgn/Crh/Fbln1/Fn1/	15
BP	GO:0050900	leukocyte migration	17/260	341/19943	2.6731E-06	0.000277048	0.00023392	Trpv4/Bst1/Emilin1/Fut7/Lbp/Adam8/Tre	17
BP	GO:0019216	regulation of lipid metabolic process	17/260	354/19943	4.4092E-06	0.000428423	0.00036172	Angptl4/Apobec1/Htr2b/Apoc1/Dab2/Igf	17
BP	GO:0031960	response to corticosteroid	16/260	321/19943	5.2951E-06	0.000504003	0.00042554	Col1a1/Sdc1/Igfbp7/Ptgs2/Ahr/Gjb2/Crh/	16
BP	GO:0006720	isoprenoid metabolic process	9/260	98/19943	5.4265E-06	0.000506184	0.00042738	Akr1b10/Adh1/Adh6/Crh/Rbp1/Hmgcs2/	9
BP	GO:0001523	retinoid metabolic process	7/260	55/19943	7.0258E-06	0.000642519	0.00054249	Akr1b10/Adh1/Adh6/Rbp1/Rarres2/Cyp2	7
BP	GO:0007584	response to nutrient	17/260	371/19943	8.1839E-06	0.00073403	0.00061975	Col1a1/Postn/Hmox1/Folr2/Spp1/Ptgs2/	17
BP	GO:0032496	response to lipopolysaccharide	20/260	498/19943	9.2923E-06	0.000817726	0.00069042	Plau/Lbp/Mrc1/Cd68/C5ar1/Igfbp3/Ptgs2	20
BP	GO:0002792	negative regulation of peptide secretion	11/260	163/19943	1.0008E-05	0.000864357	0.00072979	Tlr8/Srgn/Crh/Fbln1/Fn1/Lrrc32/Serpinb1	11
BP	GO:0001819	positive regulation of cytokine productic	19/260	462/19943	1.1215E-05	0.000938184	0.00079213	Mapk13/Trpv4/Postn/Lbp/Htr2b/Adam8/	19
BP	GO:0010811	positive regulation of cell-substrate adh	10/260	135/19943	1.1265E-05	0.000938184	0.00079213	Emilin1/Ninj1/Dab2/Spp1/Abi3bp/Nid1/F	10
BP	GO:0030595	leukocyte chemotaxis	12/260	208/19943	1.9379E-05	0.001585699	0.00133883	Trpv4/Bst1/Lbp/Adam8/Trem1/Ccl6/Spp	12
BP	GO:1902622	regulation of neutrophil migration	6/260	44/19943	2.1596E-05	0.001736632	0.00146627	Bst1/Lbp/Adam8/C5ar1/Pawr/Ednra	6
BP	GO:0051216	cartilage development	12/260	214/19943	2.5684E-05	0.002030339	0.00171425	Trpv4/Col1a1/Col20a1/Col7a1/Wnt5b/Sn	12
BP	GO:0050709	negative regulation of protein secretion	10/260	149/19943	2.6628E-05	0.00206989	0.00174764	Tlr8/Srgn/Fbln1/Fn1/Lrrc32/Serpinb1a/Ar	10
BP	GO:1905954	positive regulation of lipid localization	8/260	92/19943	2.7119E-05	0.00207352	0.00175071	Trpv4/Msr1/Pltp/Apoc4/Dab2/Spp1/Crh/	8
BP	GO:0044403	symbiont process	16/260	368/19943	2.8737E-05	0.002161742	0.0018252	Ifitm1/Hmox1/Lbp/Ifitm2/Siglec1/Trem1/	16
BP	GO:0044419	interspecies interaction between organi	17/260	410/19943	2.9528E-05	0.00218603	0.0018457	Ifitm1/Lyz2/Hmox1/Lbp/Ifitm2/Siglec1/Tr	17
BP	GO:1905952	regulation of lipid localization	10/260	153/19943	3.3437E-05	0.002436736	0.00205738	Trpv4/Msr1/Pltp/Apoc4/Apoc1/Dab2/Spp	10
BP	GO:0030593	neutrophil chemotaxis	8/260	96/19943	3.6942E-05	0.002650735	0.00223806	Bst1/Lbp/Trem1/Ccl6/Spp1/C5ar1/Ednra/	8
BP	GO:0051384	response to glucocorticoid	14/260	297/19943	3.8138E-05	0.002695056	0.00227548	Sdc1/Igfbp7/Ptgs2/Ahr/Gjb2/Crh/Fn1/Hp	14
BP	GO:0045785	positive regulation of cell adhesion	17/260	424/19943	4.4935E-05	0.003082018	0.0026022	Emilin1/Ninj1/Adam8/Dab2/Spp1/Abi3bp	17
BP	GO:0042542	response to hydrogen peroxide	11/260	194/19943	5.0425E-05	0.003307107	0.00279225	Mapk13/Cdk1/Col1a1/Hmox1/Hk3/Sdc1/	11
BP	GO:0072376	protein activation cascade	7/260	74/19943	5.0484E-05	0.003307107	0.00279225	Col20a1/C1qc/Cfd/Vsig4/Fbln1/F5/C7	7
BP	GO:0045765	regulation of angiogenesis	14/260	305/19943	5.0876E-05	0.003307107	0.00279225	Emilin1/Hmox1/Stab1/Col4a2/C5ar1/Ptgs	14
BP	GO:0071622	regulation of granulocyte chemotaxis	6/260	51/19943	5.1053E-05	0.003307107	0.00279225	Trpv4/Bst1/Lbp/C5ar1/Ednra/Rarres2	6
BP	GO:0072672	neutrophil extravasation	4/260	17/19943	5.8779E-05	0.003755398	0.00317075	Adam8/Trem1/Pawr/Prtn3	4
BP	GO:0031669	cellular response to nutrient levels	13/260	274/19943	6.7501E-05	0.004197681	0.00354417	Col1a1/Postn/Hmox1/Folr2/Cd68/Ptgs2/	13
BP	GO:0050673	epithelial cell proliferation	17/260	441/19943	7.2917E-05	0.004474793	0.00377814	Fap/Plau/Hmox1/Htr2b/Iqgap3/Dab2/C5	17
BP	GO:0098542	defense response to other organism	18/260	487/19943	7.6659E-05	0.004614664	0.00389624	Ifitm1/Lyz2/Cd8a/Lbp/Ifitm2/Apobec1/St	18
BP	GO:0001763	morphogenesis of a branching structure	12/260	240/19943	7.8133E-05	0.004614664	0.00389624	Col13a1/Col4a1/Cxcr4/Ahr/Mmp14/Ednr	12
BP	GO:0060485	mesenchyme development	13/260	278/19943	7.8164E-05	0.004614664	0.00389624	Col1a1/Htr2b/Dab2/Efnb1/Snai1/Twist1/	13
BP	GO:0070663	regulation of leukocyte proliferation	12/260	242/19943	8.4559E-05	0.004929768	0.00416229	Lst1/Bst1/Enpp3/Il20rb/Vsig4/Ahr/Pawr/	12
BP	GO:1902624	positive regulation of neutrophil migrati	5/260	36/19943	9.8174E-05	0.005506899	0.00464957	Lbp/Adam8/C5ar1/Pawr/Ednra	5
BP	GO:0050866	negative regulation of cell activation	11/260	209/19943	9.8464E-05	0.005506899	0.00464957	Lst1/Emilin1/Enpp3/Hmox1/Il20rb/Vsig4/	11
BP	GO:0062012	regulation of small molecule metabolic p	15/260	366/19943	9.904E-05	0.005506899	0.00464957	Cdk1/Slc7a7/Apoc1/Dab2/Gcgr/Igfbp3/Ie	15
BP	GO:0002526	acute inflammatory response	9/260	141/19943	9.9258E-05	0.005506899	0.00464957	Lbp/Adam8/Trem1/Il20rb/Vsig4/Ptgs2/Pl	9
BP	GO:0007162	negative regulation of cell adhesion	13/260	285/19943	0.00010036	0.005506899	0.00464957	Trpv4/Col1a1/Postn/Il20rb/Vsig4/Pawr/N	13
BP	GO:0002685	regulation of leukocyte migration	11/260	210/19943	0.00010273	0.005571268	0.00470392	Trpv4/Bst1/Emilin1/Lbp/Adam8/C5ar1/Pa	11
BP	GO:0042573	retinoic acid metabolic process	4/260	20/19943	0.00011603	0.006124583	0.00517109	Adh1/Adh6/Rbp1/Dhrs9	4
BP	GO:0061448	connective tissue development	13/260	295/19943	0.00014143	0.007270985	0.00613902	Trpv4/Col1a1/Col20a1/Col7a1/Wnt5b/Sn	13
BP	GO:0090050	positive regulation of cell migration invo	4/260	21/19943	0.00014187	0.007270985	0.00613902	Hmox1/Ptgs2/Anxa1/Rhoj	4
BP	GO:1901342	regulation of vasculature development	14/260	338/19943	0.00015104	0.007656829	0.00646479	Emilin1/Hmox1/Stab1/Col4a2/C5ar1/Ptgs	14
BP	GO:0061138	morphogenesis of a branching epitheliu	11/260	221/19943	0.00016107	0.008077531	0.00682	Col4a1/Cxcr4/Ahr/Mmp14/Ednra/Tgm2/	11
BP	GO:1905521	regulation of macrophage migration	5/260	40/19943	0.00016423	0.008108897	0.00684648	Trpv4/Emilin1/C5ar1/Mmp14/Rarres2	5
BP	GO:0034330	cell junction organization	12/260	260/19943	0.00016597	0.008108897	0.00684648	Trpv4/Gjb2/Snai1/Mmp14/Ajuba/Cdh5/F	12
BP	GO:0060326	cell chemotaxis	13/260	300/19943	0.00016691	0.008108897	0.00684648	Trpv4/Bst1/Lbp/Adam8/Trem1/Ccl6/Spp	13
BP	GO:0070664	negative regulation of leukocyte prolifer	7/260	90/19943	0.00017565	0.008445787	0.00713092	Lst1/Enpp3/Il20rb/Vsig4/Pawr/Lrrc32/Ce	7
BP	GO:1903672	positive regulation of sprouting angioge	5/260	41/19943	0.00018506	0.008795632	0.0074263	Hmox1/Ptgs2/Anxa1/Rhoj/Itga5	5
BP	GO:0002695	negative regulation of leukocyte activati	10/260	188/19943	0.0001867	0.008795632	0.0074263	Lst1/Enpp3/Hmox1/Il20rb/Vsig4/Pawr/Pg	10
BP	GO:0002062	chondrocyte differentiation	8/260	123/19943	0.00021221	0.009850687	0.0083171	Col20a1/Col7a1/Wnt5b/Wnt2b/Ccn2/Mu	8
BP	GO:0051249	regulation of lymphocyte activation	16/260	437/19943	0.00021332	0.009850687	0.0083171	Lst1/Bst1/Lat/Adam8/Il20rb/Vsig4/LOC10	16
BP	GO:0050670	regulation of lymphocyte proliferation	11/260	230/19943	0.0002278	0.010409443	0.00878887	Lst1/Bst1/Il20rb/Vsig4/Ahr/Pawr/Efnb1/L	11
BP	GO:0031668	cellular response to extracellular stimulu	13/260	310/19943	0.00022988	0.010409443	0.00878887	Col1a1/Postn/Hmox1/Folr2/Cd68/Ptgs2/	13
BP	GO:0032944	regulation of mononuclear cell prolifera	11/260	232/19943	0.00024544	0.010939344	0.00923627	Lst1/Bst1/Il20rb/Vsig4/Ahr/Pawr/Efnb1/L	11
BP	GO:0050715	positive regulation of cytokine secretion	9/260	159/19943	0.00024628	0.010939344	0.00923627	Trpv4/Postn/Htr2b/Tlr8/Twist1/Osm/Lilra	9
BP	GO:0070661	leukocyte proliferation	13/260	317/19943	0.00028522	0.012261418	0.01035252	Lst1/Bst1/Enpp3/Il20rb/Vsig4/Cxcr4/Ahr/	13
BP	GO:0060536	cartilage morphogenesis	4/260	25/19943	0.00028781	0.012261418	0.01035252	Col20a1/Col7a1/Snai1/Snai2	4
BP	GO:1905523	positive regulation of macrophage migra	4/260	25/19943	0.00028781	0.012261418	0.01035252	Trpv4/C5ar1/Mmp14/Rarres2	4
BP	GO:0001503	ossification	15/260	404/19943	0.00028905	0.012261418	0.01035252	Col13a1/Col1a1/Spp1/Igfbp3/Srgn/Ptgs2/	15
BP	GO:0035987	endodermal cell differentiation	5/260	45/19943	0.00028918	0.012261418	0.01035252	Col7a1/Col4a2/Mmp14/Fn1/Itga5	5
BP	GO:0036293	response to decreased oxygen levels	16/260	453/19943	0.00031838	0.01336758	0.01128648	Angptl4/Plau/Postn/Hmox1/Adam8/Igfbp	16
BP	GO:0010565	regulation of cellular ketone metabolic p	8/260	131/19943	0.00032603	0.01336758	0.01128648	Slc7a7/Apoc1/Dab2/Ptgs2/C1qtnf2/Twist	8
BP	GO:0070206	protein trimerization	6/260	71/19943	0.00032674	0.01336758	0.01128648	Col1a1/Emilin1/C1qtnf2/Lcn2/Ceacam1/	6
BP	GO:1903670	regulation of sprouting angiogenesis	6/260	71/19943	0.00032674	0.01336758	0.01128648	Hmox1/Ptgs2/Anxa1/Ceacam1/Rhoj/Itga	6
BP	GO:1904018	positive regulation of vasculature develo	10/260	203/19943	0.00034571	0.014020864	0.01183805	Hmox1/C5ar1/Ptgs2/Cxcr4/Cdh5/Anxa1/	10
BP	GO:0042180	cellular ketone metabolic process	10/260	204/19943	0.00035944	0.014328543	0.01209783	Slc7a7/Apoc1/Dab2/Ptgs2/C1qtnf2/Akr1	10
BP	GO:0051224	negative regulation of protein transport	10/260	204/19943	0.00035944	0.014328543	0.01209783	Tlr8/Srgn/Fbln1/Fn1/Lrrc32/Serpinb1a/Ar	10
BP	GO:0048525	negative regulation of viral process	7/260	102/19943	0.0003804	0.015035413	0.01269466	Ifitm1/Hmox1/Ifitm2/Plscr1/Fbln1/Ceaca	7
BP	GO:0007179	transforming growth factor beta recept	9/260	169/19943	0.00038641	0.015144579	0.01278683	Htra3/Emilin1/Ltbp2/Dab2/Cdh5/Lrrc32/	9

BP	GO:0050873	brown fat cell differentiation	5/260	49/19943	0.00043263	0.016659081	0.01406555	Trpv4/Ptgs2/Arl4a/Rarres2/Lrg1	5
BP	GO:1904950	negative regulation of establishment of	10/260	209/19943	0.00043511	0.016659081	0.01406555	Tlr8/Srgn/Fbln1/Fn1/Lrrc32/Serpinb1a/Ar	10
BP	GO:0061299	retina vasculature morphogenesis in car	3/260	12/19943	0.00044171	0.016659081	0.01406555	Col4a1/Lama1/Rhoj	3
BP	GO:0035455	response to interferon-alpha	4/260	28/19943	0.00045175	0.016659081	0.01406555	Ifitm1/Ifitm2/Plscr1/Ifitm6	4
BP	GO:1903557	positive regulation of tumor necrosis fac	7/260	105/19943	0.00045362	0.016659081	0.01406555	Lbp/Adam8/Twist1/Lilra5/Pf4/Cyp2j4/Spo	7
BP	GO:0006953	acute-phase response	5/260	50/19943	0.00047564	0.017331138	0.01463298	Lbp/Ptgs2/Plscr1/Fn1/Hp	5
BP	GO:0001666	response to hypoxia	15/260	424/19943	0.00047984	0.017348774	0.01464787	Angptl4/Plau/Postn/Hmox1/Adam8/Igfbp	15
BP	GO:0071496	cellular response to external stimulus	15/260	425/19943	0.0004917	0.017640769	0.0148944	Col1a1/Postn/Hmox1/Folr2/Cd68/Tlr8/Pt	15
BP	GO:0007219	Notch signaling pathway	9/260	175/19943	0.00049823	0.017695218	0.01494037	Postn/Cfd/Snai1/Mmp14/Pear1/Hp/Snai2	9
BP	GO:0060350	endochondral bone morphogenesis	6/260	77/19943	0.00050688	0.017695218	0.01494037	Col13a1/Trpv4/Col1a1/Col20a1/Col7a1/M	6
BP	GO:0032755	positive regulation of interleukin-6 prod	7/260	107/19943	0.00050839	0.017695218	0.01494037	Mapk13/Trpv4/Lbp/Tlr8/Twist1/Lilra5/Sp	7
BP	GO:0006929	substrate-dependent cell migration	4/260	29/19943	0.0005187	0.017695218	0.01494037	Adam8/Fbln1/Fn1/Snai2	4
BP	GO:0001706	endoderm formation	5/260	51/19943	0.00052179	0.017695218	0.01494037	Col7a1/Col4a2/Mmp14/Fn1/Itga5	5
BP	GO:0000302	response to reactive oxygen species	12/260	295/19943	0.00052236	0.017695218	0.01494037	Mapk13/Cdk1/Col1a1/Hmox1/Hk3/Sdc1/	12
BP	GO:0034329	cell junction assembly	10/260	214/19943	0.00052357	0.017695218	0.01494037	Trpv4/Gjb2/Snai1/Mmp14/Ajuba/Cdh5/F	10
BP	GO:0046651	lymphocyte proliferation	12/260	296/19943	0.00053825	0.018020656	0.01521515	Lst1/Bst1/Il20rb/Vsig4/Cxcr4/Ahr/Pawr/E	12
BP	GO:0046890	regulation of lipid biosynthetic process	9/260	177/19943	0.00054093	0.018020656	0.01521515	Htr2b/Apoc1/Dab2/Igfbp7/Ptgs2/Snai1/A	9
BP	GO:0032963	collagen metabolic process	7/260	109/19943	0.0005683	0.018502485	0.01562196	Col1a1/Fap/Emilin1/Mmp14/Ccn2/Cyp2j4	7
BP	GO:0042362	fat-soluble vitamin biosynthetic process	3/260	13/19943	0.0005687	0.018502485	0.01562196	Pltp/Snai1/Snai2	3
BP	GO:0032943	mononuclear cell proliferation	12/260	298/19943	0.00057126	0.018502485	0.01562196	Lst1/Bst1/Il20rb/Vsig4/Cxcr4/Ahr/Pawr/E	12
BP	GO:0007492	endoderm development	6/260	79/19943	0.00058153	0.018638887	0.01573713	Col7a1/Col4a2/Dab2/Mmp14/Fn1/Itga5	6
BP	GO:0048762	mesenchymal cell differentiation	10/260	217/19943	0.00058346	0.018638887	0.01573713	Col1a1/Htr2b/Dab2/Efnb1/Snai1/Twist1/	10
BP	GO:0060706	cell differentiation involved in embryoni	4/260	30/19943	0.00059242	0.01866921	0.01576273	Snai1/Krt8/Mdfi/Krt19	4
BP	GO:0050729	positive regulation of inflammatory resp	8/260	144/19943	0.00061357	0.019205937	0.0162159	Trpv4/Lbp/Adam8/Ptgs2/Ednra/Tgm2/Os	8
BP	GO:1905517	macrophage migration	5/260	53/19943	0.0006241	0.019405259	0.01638419	Trpv4/Emilin1/C5ar1/Mmp14/Rarres2	5
BP	GO:0019835	cytolysis	4/260	31/19943	0.00067327	0.020658819	0.01744259	Lyz2/Lbp/Pglyrp1/Pf4	4
BP	GO:0010712	regulation of collagen metabolic process	5/260	54/19943	0.00068055	0.020745639	0.0175159	Fap/Emilin1/Ccn2/Cyp2j4/Ltbp1	5
BP	GO:0002687	positive regulation of leukocyte migratic	8/260	147/19943	0.00070276	0.021229162	0.01792414	Trpv4/Lbp/Adam8/C5ar1/Pawr/Mmp14/E	8
BP	GO:0048705	skeletal system morphogenesis	11/260	263/19943	0.00070551	0.021229162	0.01792414	Col13a1/Trpv4/Col1a1/Col20a1/Col7a1/M	11
BP	GO:0045766	positive regulation of angiogenesis	9/260	184/19943	0.00071468	0.021295439	0.0179801	Hmox1/C5ar1/Ptgs2/Cxcr4/Cdh5/Anxa1/I	9
BP	GO:0042268	regulation of cytolysis	3/260	14/19943	0.00071685	0.021295439	0.0179801	Lbp/Pglyrp1/Pf4	3
BP	GO:0032964	collagen biosynthetic process	5/260	55/19943	0.00074074	0.021731885	0.0183486	Col1a1/Emilin1/Ccn2/Cyp2j4/Ltbp1	5
BP	GO:0034754	cellular hormone metabolic process	7/260	114/19943	0.00074284	0.021731885	0.0183486	Dab2/Spp1/Akr1b10/Adh1/Adh6/Rbp1/D	7
BP	GO:0002274	myeloid leukocyte activation	10/260	224/19943	0.00074552	0.021731885	0.0183486	Batf3/Enpp3/Hmox1/Lbp/Lat/Vsig4/Tlr8/	10
BP	GO:0090022	regulation of neutrophil chemotaxis	4/260	32/19943	0.00076164	0.022063811	0.01862885	Bst1/Lbp/C5ar1/Ednra	4
BP	GO:0045216	cell-cell junction organization	8/260	149/19943	0.00076781	0.022105375	0.01866394	Trpv4/Gjb2/Snai1/Cdh5/Ceacam1/Lsr/Prt	8
BP	GO:0008015	blood circulation	16/260	493/19943	0.00079429	0.022612296	0.01909195	Trpv4/Slc4a5/Postn/Hmox1/Htr2b/Ier3/P	16
BP	GO:0043903	regulation of symbiosis, encompassing r	10/260	226/19943	0.00079809	0.022612296	0.01909195	Ifitm1/Hmox1/Lbp/Ifitm2/Cxcr4/Plscr1/Cl	10
BP	GO:0032526	response to retinoic acid	8/260	150/19943	0.00080211	0.022612296	0.01909195	Col1a1/Igfbp7/Stra6/Gjb2/Adh1/Igfbp2/F	8
BP	GO:0033280	response to vitamin D	5/260	56/19943	0.00080481	0.022612296	0.01909195	Spp1/Ptgs2/Kank2/Ltbp1/Snai2	5
BP	GO:0014032	neural crest cell development	6/260	85/19943	0.00085754	0.023536435	0.01987221	Htr2b/Efnb1/Twist1/Ednra/Fn1/Snai2	6
BP	GO:1903901	negative regulation of viral life cycle	6/260	85/19943	0.00085754	0.023536435	0.01987221	Ifitm1/Hmox1/Ifitm2/Plscr1/Ceacam1/Ifit	6
BP	GO:0006775	fat-soluble vitamin metabolic process	4/260	33/19943	0.00085789	0.023536435	0.01987221	Pltp/Snai1/Rbp1/Snai2	4
BP	GO:2000778	positive regulation of interleukin-6 secre	4/260	33/19943	0.00085789	0.023536435	0.01987221	Trpv4/Tlr8/Twist1/Lilra5	4
BP	GO:0015850	organic hydroxy compound transport	11/260	270/19943	0.00087478	0.023621422	0.01994397	RGD1305807/Msr1/Pltp/Apoc1/Dab2/Slc	11
BP	GO:0031099	regeneration	12/260	313/19943	0.00087739	0.023621422	0.01994397	Cdk1/Plau/Postn/Hmox1/Ninj1/Spp1/C5a	12
BP	GO:0042953	lipoprotein transport	3/260	15/19943	0.00088746	0.023621422	0.01994397	Msr1/Apobec1/Cubn	3
BP	GO:0044872	lipoprotein localization	3/260	15/19943	0.00088746	0.023621422	0.01994397	Msr1/Apobec1/Cubn	3
BP	GO:0047484	regulation of response to osmotic stress	3/260	15/19943	0.00088746	0.023621422	0.01994397	Trpv4/Ptgs2/Ptger2	3
BP	GO:0006959	humoral immune response	9/260	190/19943	0.00089762	0.023621422	0.01994397	Col20a1/C1qc/Cfd/Vsig4/Pglyrp1/Rarres2	9
BP	GO:0050792	regulation of viral process	9/260	190/19943	0.00089762	0.023621422	0.01994397	Ifitm1/Hmox1/Ifitm2/Cxcr4/Plscr1/Chmp	9
BP	GO:0032945	negative regulation of mononuclear cell	6/260	86/19943	0.00091196	0.023629864	0.0199511	Lst1/Il20rb/Vsig4/Pawr/Lrrc32/Ceacam1	6
BP	GO:0050672	negative regulation of lymphocyte prolif	6/260	86/19943	0.00091196	0.023629864	0.0199511	Lst1/Il20rb/Vsig4/Pawr/Lrrc32/Ceacam1	6
BP	GO:0045071	negative regulation of viral genome repl	5/260	58/19943	0.00094525	0.024357205	0.0205652	Ifitm1/Ifitm2/Plscr1/Ceacam1/Ifitm6	5
BP	GO:0042572	retinol metabolic process	4/260	34/19943	0.00096241	0.024662951	0.02082335	Akr1b10/Adh1/Adh6/Rbp1	4
BP	GO:0043901	negative regulation of multi-organism pr	9/260	193/19943	0.00100241	0.025487452	0.02151949	Ifitm1/Hmox1/Lbp/Ifitm2/Plscr1/Fbln1/C	9
BP	GO:0001704	formation of primary germ layer	7/260	120/19943	0.00100551	0.025487452	0.02151949	Col7a1/Col4a2/Snai1/Mmp14/Fn1/Fzd7/I	7
BP	GO:0032103	positive regulation of response to exterr	12/260	320/19943	0.00106135	0.026695272	0.02253927	Trpv4/Lbp/Adam8/C5ar1/Ptgs2/Cxcr4/Ed	12
BP	GO:0009266	response to temperature stimulus	10/260	235/19943	0.00107386	0.026695272	0.02253927	Trpv4/Hmox1/Htr2b/Igfbp7/Ptgs2/Cxcr4/	10
BP	GO:0051180	vitamin transport	4/260	35/19943	0.00107556	0.026695272	0.02253927	RGD1305807/Folr2/Stra6/Cubn	4
BP	GO:0043518	negative regulation of DNA damage resp	3/260	16/19943	0.00108178	0.026695272	0.02253927	Snai1/Twist1/Snai2	3
BP	GO:0060192	negative regulation of lipase activity	3/260	16/19943	0.00108178	0.026695272	0.02253927	Angptl4/Apoc1/Anxa1	3
BP	GO:0006956	complement activation	5/260	60/19943	0.00110314	0.027047568	0.02283672	Col20a1/C1qc/Cfd/Vsig4/C7	5
BP	GO:0060349	bone morphogenesis	7/260	122/19943	0.00110765	0.027047568	0.02283672	Col13a1/Trpv4/Col1a1/Col20a1/Col7a1/M	7
BP	GO:0051250	negative regulation of lymphocyte activa	8/260	158/19943	0.00112313	0.027282808	0.02303534	Lst1/Il20rb/Vsig4/Pawr/Pglyrp1/Lrrc32/A	8
BP	GO:0014031	mesenchymal cell development	6/260	90/19943	0.00115666	0.02795154	0.02359996	Htr2b/Efnb1/Twist1/Ednra/Fn1/Snai2	6
BP	GO:0010812	negative regulation of cell-substrate adh	5/260	61/19943	0.00118903	0.028439058	0.02401158	Col1a1/Postn/Mmp14/Fbln1/Fzd7	5
BP	GO:0009615	response to virus	11/260	281/19943	0.0012079	0.02871098	0.02424117	Batf3/Ifitm1/Cd8a/Ifitm2/Apobec1/Tlr8/F	11
BP	GO:0032368	regulation of lipid transport	7/260	124/19943	0.00121777	0.02871098	0.02424117	Trpv4/Pltp/Apoc1/Dab2/Spp1/Igfbp3/Crh	7
BP	GO:0019217	regulation of fatty acid metabolic proces	6/260	91/19943	0.00122502	0.02871098	0.02424117	Apoc1/Ptgs2/C1qtnf2/Twist1/Anxa1/Cea	6
BP	GO:0048864	stem cell development	6/260	91/19943	0.00122502	0.02871098	0.02424117	Htr2b/Efnb1/Twist1/Ednra/Fn1/Snai2	6
BP	GO:0050678	regulation of epithelial cell proliferation	13/260	371/19943	0.0012321	0.028732473	0.02425932	Plau/Hmox1/Htr2b/Iqgap3/Dab2/C5ar1/T	13
BP	GO:0014033	neural crest cell differentiation	6/260	92/19943	0.00129643	0.029313139	0.02474958	Htr2b/Efnb1/Twist1/Ednra/Fn1/Snai2	6
BP	GO:0045833	negative regulation of lipid metabolic pr	6/260	92/19943	0.00129643	0.029313139	0.02474958	Apobec1/Apoc1/Snai1/Cidea/Ceacam1/Sı	6
BP	GO:0016114	terpenoid biosynthetic process	3/260	17/19943	0.00130099	0.029313139	0.02474958	Rbp1/Hmgcs2/Dhrs9	3
BP	GO:0035461	vitamin transmembrane transport	3/260	17/19943	0.00130099	0.029313139	0.02474958	RGD1305807/Folr2/Stra6	3
BP	GO:0070234	positive regulation of T cell apoptotic pr	3/260	17/19943	0.00130099	0.029313139	0.02474958	Adam8/Siglec1/Ceacam1	3
BP	GO:2000108	positive regulation of leukocyte apoptot	4/260	37/19943	0.0013293	0.02966441	0.02504617	Adam8/Siglec1/Anxa1/Ceacam1	4
BP	GO:0015718	monocarboxylic acid transport	8/260	163/19943	0.00137098	0.030404698	0.02567121	Trpv4/Slco2a1/RGD1305807/Slc16a3/Stra	8
BP	GO:0045600	positive regulation of fat cell differentiat	5/260	63/19943	0.00137551	0.030404698	0.02567121	Ptgs2/Wnt5b/Rarres2/Snai2/Zbtb7c	5
BP	GO:0042246	tissue regeneration	6/260	94/19943	0.00144875	0.031872559	0.02691055	Plau/Postn/Ninj1/Fzd7/Mustn1/Anxa1	6
BP	GO:0035988	chondrocyte proliferation	3/260	18/19943	0.00154623	0.033801113	0.02853886	Mmp14/Ccn2/Mustn1	3
BP	GO:0043900	regulation of multi-organism process	14/260	428/19943	0.00155091	0.033801113	0.02853886	Cdk1/Ifitm1/Plau/Hmox1/Lbp/Ifitm2/Cxcı	14
BP	GO:0016032	viral process	11/260	291/19943	0.00159535	0.034505856	0.02913388	Ifitm1/Hmox1/Ifitm2/Siglec1/Lrrc15/Cxcr	11
BP	GO:0045995	regulation of embryonic development	7/260	130/19943	0.00160025	0.034505856	0.02913388	Snai1/Lama2/Wnt2b/Fzd7/Lama1/Six1/Pt	7
BP	GO:0090049	regulation of cell migration involved in s	4/260	39/19943	0.00162211	0.034505856	0.02913388	Hmox1/Ptgs2/Anxa1/Rhoj	4
BP	GO:0031349	positive regulation of defense response	13/260	383/19943	0.00163579	0.034505856	0.02913388	Trpv4/Lbp/Adam8/Tlr8/LOC102547056/P	13
BP	GO:0032102	negative regulation of response to exter	12/260	337/19943	0.001645	0.034505856	0.02913388	Plau/Enpp3/Il20rb/Spp1/Ier3/Tspan8/Kar	12
BP	GO:0010957	negative regulation of vitamin D biosynt	2/260	5/19943	0.00164983	0.034505856	0.02913388	Snai1/Snai2	2
BP	GO:0038044	transforming growth factor-beta secreti	2/260	5/19943	0.00164983	0.034505856	0.02913388	Fbln1/Fn1	2
BP	GO:0042098	T cell proliferation	9/260	208/19943	0.00168545	0.035093381	0.02962994	Il20rb/Vsig4/Cxcr4/Pawr/Efnb1/Lrrc32/Ig	9
BP	GO:0046718	viral entry into host cell	5/260	66/19943	0.00169452	0.035125424	0.02965699	Ifitm1/Ifitm2/Siglec1/Ceacam1/Ifitm6	5
BP	GO:2000027	regulation of animal organ morphogene	9/260	209/19943	0.00174171	0.035785631	0.03021442	Plau/Dab2/Twist1/Ednra/Wnt2b/Ceacam	9
BP	GO:0001569	branching involved in blood vessel morp	4/260	40/19943	0.0017841	0.036495728	0.03081397	Col4a1/Cxcr4/Ahr/Ednra	4
BP	GO:1903555	regulation of tumor necrosis factor supe	8/260	171/19943	0.0018561	0.037550363	0.03170441	Lbp/Adam8/Twist1/Cidea/Lilra5/Pf4/Cyp2	8
BP	GO:0032355	response to estradiol	10/260	253/19943	0.00185981	0.037550363	0.03170441	Col1a1/Postn/Igfbp3/Ptgs2/Ahr/Pawr/Gjl	10
BP	GO:0071222	cellular response to lipopolysaccharide	11/260	298/19943	0.00192291	0.038657048	0.03263881	Plau/Lbp/Mrc1/Cd68/Plscr1/Plscr2/Lcn2/	11
BP	GO:0070371	ERK1 and ERK2 cascade	12/260	344/19943	0.00195205	0.039005489	0.032933	Trpv4/Emilin1/Htr2b/Iqgap3/Dab2/Ccl6/C	12
BP	GO:0045923	positive regulation of fatty acid metabol	4/260	41/19943	0.00195696	0.039005489	0.032933	Ptgs2/C1qtnf2/Twist1/Anxa1	4
BP	GO:0042129	regulation of T cell proliferation	8/260	173/19943	0.00199633	0.03962079	0.03345251	Il20rb/Vsig4/Pawr/Efnb1/Lrrc32/Igfbp2/A	8
BP	GO:0030260	entry into host cell	5/260	69/19943	0.0020647	0.040067088	0.03382933	Ifitm1/Ifitm2/Siglec1/Ceacam1/Ifitm6	5

BP	GO:0032623	interleukin-2 production	5/260	69/19943	0.0020647	0.040067088	0.03382933	Il20rb/Vsig4/Pawr/Anxa1/Ceacam1	5
BP	GO:0044409	entry into host	5/260	69/19943	0.0020647	0.040067088	0.03382933	Ifitm1/Ifitm2/Siglec1/Ceacam1/Ifitm6	5
BP	GO:0051806	entry into cell of other organism involve	5/260	69/19943	0.0020647	0.040067088	0.03382933	Ifitm1/Ifitm2/Siglec1/Ceacam1/Ifitm6	5
BP	GO:0051828	entry into other organism involved in sy	5/260	69/19943	0.0020647	0.040067088	0.03382933	Ifitm1/Ifitm2/Siglec1/Ceacam1/Ifitm6	5
BP	GO:0051701	interaction with host	7/260	136/19943	0.00207036	0.040067088	0.03382933	Ifitm1/Ifitm2/Siglec1/Chmp4c/Fbln1/Cea	7
BP	GO:0010759	positive regulation of macrophage chem	3/260	20/19943	0.00211901	0.040339034	0.03405893	Trpv4/C5ar1/Rarres2	3
BP	GO:0030852	regulation of granulocyte differentiation	3/260	20/19943	0.00211901	0.040339034	0.03405893	C1qc/Rbp1/Ceacam1	3
BP	GO:0071706	tumor necrosis factor superfamily cytok	8/260	175/19943	0.00214478	0.040663572	0.03433295	Lbp/Adam8/Twist1/Cidea/Lilra5/Pf4/Cyp	8
BP	GO:0042130	negative regulation of T cell proliferatior	5/260	70/19943	0.00220026	0.041546568	0.03507848	Il20rb/Vsig4/Pawr/Lrrc32/Ceacam1	5
BP	GO:0002698	negative regulation of immune effector	7/260	138/19943	0.00224872	0.042290427	0.03570653	Enpp3/Hmox1/Il20rb/Vsig4/Pglyrp1/Anxa	7
BP	GO:0019058	viral life cycle	9/260	218/19943	0.00231886	0.04334457	0.03659656	Ifitm1/Hmox1/Ifitm2/Siglec1/Lrrc15/Plscr	9
BP	GO:0042304	regulation of fatty acid biosynthetic proc	4/260	43/19943	0.00233681	0.04334457	0.03659656	Apoc1/Ptgs2/Anxa1/Ceacam1	4
BP	GO:0071219	cellular response to molecule of bacteri	11/260	307/19943	0.00242255	0.04334457	0.03659656	Plau/Lbp/Mrc1/Cd68/Plscr1/Plscr2/Lcn2/	11
BP	GO:0032760	positive regulation of tumor necrosis fac	6/260	104/19943	0.00242438	0.04334457	0.03659656	Lbp/Twist1/Lilra5/Pf4/Cyp2j4/Spon2	6
BP	GO:0010884	positive regulation of lipid storage	3/260	21/19943	0.00244853	0.04334457	0.03659656	Msr1/Apoc4/Cidea	3
BP	GO:0006069	ethanol oxidation	2/260	6/19943	0.00245347	0.04334457	0.03659656	Adh1/Adh6	2
BP	GO:0033015	tetrapyrrole catabolic process	2/260	6/19943	0.00245347	0.04334457	0.03659656	Hmox1/Cubn	2
BP	GO:0046137	negative regulation of vitamin metabolic	2/260	6/19943	0.00245347	0.04334457	0.03659656	Snai1/Snai2	2
BP	GO:0051005	negative regulation of lipoprotein lipase	2/260	6/19943	0.00245347	0.04334457	0.03659656	Angptl4/Apoc1	2
BP	GO:0061304	retinal blood vessel morphogenesis	2/260	6/19943	0.00245347	0.04334457	0.03659656	Col4a1/Lama1	2
BP	GO:0070562	regulation of vitamin D receptor signalin	2/260	6/19943	0.00245347	0.04334457	0.03659656	Kank2/Snai2	2
BP	GO:1902219	negative regulation of intrinsic apoptoti	2/260	6/19943	0.00245347	0.04334457	0.03659656	Ptgs2/Ptger2	2
BP	GO:0042742	defense response to bacterium	10/260	264/19943	0.00253429	0.044435811	0.03751791	Lyz2/Lbp/Stab1/Trem1/C5ar1/Pglyrp1/Lc	10
BP	GO:0035456	response to interferon-beta	4/260	44/19943	0.00254451	0.044447966	0.03752818	Ifitm1/Ifitm2/Plscr1/Ifitm6	4
BP	GO:0042445	hormone metabolic process	9/260	222/19943	0.00261986	0.045593389	0.03849528	Pon3/Dab2/Spp1/Akr1b10/Adh1/Adh6/C	9
BP	GO:0032370	positive regulation of lipid transport	5/260	73/19943	0.00264586	0.045874714	0.0387328	Trpv4/Pltp/Dab2/Spp1/Crh	5
BP	GO:0017015	regulation of transforming growth facto	6/260	106/19943	0.00266763	0.046080812	0.03890682	Htra3/Emilin1/Dab2/Cidea/Ltbp1/Lrg1	6
BP	GO:0060416	response to growth hormone	4/260	45/19943	0.00276454	0.04748597	0.04009321	Igfbp3/Hp/Hmgcs2/Gdf15	4
BP	GO:0051222	positive regulation of protein transport	14/260	456/19943	0.00276934	0.04748597	0.04009321	Trpv4/Postn/Htr2b/Tlr8/Ptgs2/Twist1/Ce	14
BP	GO:0009110	vitamin biosynthetic process	3/260	22/19943	0.00280805	0.04797337	0.04050473	Pltp/Snai1/Snai2	3
BP	GO:0015711	organic anion transport	14/260	458/19943	0.00288025	0.049027258	0.04139455	Trpv4/Slco2a1/Slc7a7/Slc4a5/RGD13058	14
BP	GO:0001906	cell killing	8/260	184/19943	0.00292345	0.049135343	0.04148581	Lyz2/Trem1/RT1-M2/LOC102547056/Cdh	8
BP	GO:0048754	branching morphogenesis of an epitheli	8/260	184/19943	0.00292345	0.049135343	0.04148581	Col4a1/Cxcr4/Ahr/Mmp14/Ednra/Wnt2b,	8
BP	GO:0008630	intrinsic apoptotic signaling pathway in r	6/260	108/19943	0.00292874	0.049135343	0.04148581	Hmox1/Ier3/Plscr1/Snai1/Uaca/Snai2	6
BP	GO:1903844	regulation of cellular response to transf	6/260	108/19943	0.00292874	0.049135343	0.04148581	Htra3/Emilin1/Dab2/Cidea/Ltbp1/Lrg1	6
BP	GO:0032965	regulation of collagen biosynthetic proc	4/260	46/19943	0.00299726	0.049925791	0.0421532	Emilin1/Ccn2/Cyp2j4/Ltbp1	4
BP	GO:0070207	protein homotrimerization	4/260	46/19943	0.00299726	0.049925791	0.0421532	Emilin1/Lcn2/Ceacam1/Cubn	4
BP	GO:0022408	negative regulation of cell-cell adhesion	8/260	185/19943	0.00302201	0.04999065	0.04220796	Trpv4/Il20rb/Vsig4/Pawr/Lrrc32/Anxa1/C	8
BP	GO:0050714	positive regulation of protein secretion	11/260	316/19943	0.00302259	0.04999065	0.04220796	Trpv4/Postn/Htr2b/Tlr8/Twist1/Crh/Ceac	11
CC	GO:0062023	collagen-containing extracellular matrix	28/260	217/19943	6.209E-20	2.89587E-16	2.445E-16	Col1a1/Emilin1/Col20a1/Postn/Col4a1/C	28
CC	GO:0031012	extracellular matrix	34/260	362/19943	1.5476E-19	3.60906E-16	3.0472E-16	Col13a1/Col1a1/Emilin1/Col20a1/Postn/	34
CC	GO:0044420	extracellular matrix component	12/260	51/19943	1.8918E-12	2.20582E-09	1.8624E-09	Col1a1/Emilin1/Col4a1/Col4a2/Col18a1/	12
CC	GO:0005581	collagen trimer	12/260	68/19943	7.132E-11	5.54397E-08	4.6809E-08	Col13a1/Col1a1/Emilin1/C1qc/Col4a1/Co	12
CC	GO:0005604	basement membrane	14/260	105/19943	9.2179E-11	6.14175E-08	5.1856E-08	Col4a1/Col7a1/Col4a2/Col15a1/LOC1083	14
CC	GO:0098651	basement membrane collagen trimer	5/260	7/19943	7.4515E-09	3.15944E-06	2.6676E-06	Col4a1/Col4a2/Col18a1/Col4a5/Col4a6	5
CC	GO:0005587	collagen type IV trimer	4/260	6/19943	4.1488E-07	6.91068E-05	5.8348E-05	Col4a1/Col4a2/Col4a5/Col4a6	4
CC	GO:0098642	network-forming collagen trimer	4/260	6/19943	4.1488E-07	6.91068E-05	5.8348E-05	Col4a1/Col4a2/Col4a5/Col4a6	4
CC	GO:0098645	collagen network	4/260	6/19943	4.1488E-07	6.91068E-05	5.8348E-05	Col4a1/Col4a2/Col4a5/Col4a6	4
CC	GO:0098644	complex of collagen trimers	5/260	16/19943	1.4076E-06	0.000164131	0.00013858	Col1a1/Col4a1/Col4a2/Col4a5/Col4a6	5
CC	GO:0034358	plasma lipoprotein particle	4/260	28/19943	0.00045175	0.016659081	0.01406555	Pltp/Apoc4/Apoc1/Lsr	4
CC	GO:1990777	lipoprotein particle	4/260	28/19943	0.00045175	0.016659081	0.01406555	Pltp/Apoc4/Apoc1/Lsr	4
CC	GO:0032994	protein-lipid complex	4/260	30/19943	0.00059242	0.01866921	0.01576273	Pltp/Apoc4/Apoc1/Lsr	4
CC	GO:0034361	very-low-density lipoprotein particle	3/260	17/19943	0.00130099	0.029313139	0.02474958	Apoc4/Apoc1/Lsr	3
CC	GO:0034385	triglyceride-rich plasma lipoprotein parti	3/260	17/19943	0.00130099	0.029313139	0.02474958	Apoc4/Apoc1/Lsr	3
CC	GO:0016942	insulin-like growth factor binding protei	2/260	5/19943	0.00164983	0.034505856	0.02913388	Igfbp3/Igfbp6	2
CC	GO:0036454	growth factor complex	2/260	5/19943	0.00164983	0.034505856	0.02913388	Igfbp3/Igfbp6	2
CC	GO:0031091	platelet alpha granule	3/260	19/19943	0.00181856	0.037038326	0.03127209	Igfbp3/Pf4/F5	3
CC	GO:0042629	mast cell granule	3/260	20/19943	0.00211901	0.040339034	0.03405893	Lat/Srgn/Anxa1	3
CC	GO:0009897	external side of plasma membrane	13/260	399/19943	0.00233918	0.04334457	0.03659656	Cd8a/Enpp3/Scart1/Folr2/Tlr8/RT1-M2/S	13
CC	GO:0034364	high-density lipoprotein particle	3/260	21/19943	0.00244853	0.04334457	0.03659656	Pltp/Apoc4/Apoc1	3
MF	GO:0005201	extracellular matrix structural constitue	14/260	62/19943	4.8072E-14	7.47359E-11	6.3101E-11	Col13a1/Col1a1/Emilin1/Col4a1/Col4a2/	14
MF	GO:0005178	integrin binding	11/260	129/19943	1.0261E-06	0.00012934	0.0001092	Fap/Emilin1/Dab2/Spp1/Tspan8/Mmp14/	11
MF	GO:0005520	insulin-like growth factor binding	6/260	28/19943	1.3726E-06	0.000164131	0.00013858	Htra3/Igfbp3/Igfbp7/Ccn2/Igfbp2/Igfbp6	6
MF	GO:0050839	cell adhesion molecule binding	14/260	232/19943	2.3268E-06	0.00025838	0.00021815	Fap/Emilin1/Postn/Adam8/Dab2/Spp1/Ts	14
MF	GO:0038024	cargo receptor activity	9/260	89/19943	2.4269E-06	0.000263237	0.00022226	Msr1/Enpp3/Mrc1/Stab1/Scart1/Folr2/D	9
MF	GO:0019838	growth factor binding	11/260	146/19943	3.4733E-06	0.000352162	0.00029734	Col1a1/Htra3/Ltbp2/Col4a1/Igfbp3/Igfbp	11
MF	GO:0005539	glycosaminoglycan binding	13/260	209/19943	3.85E-06	0.000382053	0.00032257	Col13a1/Ltbp2/Postn/Stab1/Abi3bp/Prel	13
MF	GO:0008201	heparin binding	10/260	157/19943	4.1684E-05	0.002901711	0.00244996	Col13a1/Ltbp2/Postn/Abi3bp/Prelp/Fn1/	10
MF	GO:0001968	fibronectin binding	5/260	33/19943	6.3808E-05	0.004021624	0.00339553	Lrrc15/Igfbp3/Fbln1/Ccn2/Igfbp6	5
MF	GO:0004022	alcohol dehydrogenase (NAD) activity	3/260	8/19943	0.00011687	0.006124583	0.00517109	Adh1/Adh6/Dhrs9	3
MF	GO:0031995	insulin-like growth factor II binding	3/260	8/19943	0.00011687	0.006124583	0.00517109	Igfbp3/Igfbp2/Igfbp6	3
MF	GO:0031994	insulin-like growth factor I binding	3/260	12/19943	0.00044171	0.016659081	0.01406555	Igfbp3/Igfbp2/Igfbp6	3
MF	GO:0005044	scavenger receptor activity	5/260	51/19943	0.00052179	0.017695218	0.01494037	Msr1/Enpp3/Stab1/Scart1/Scarf2	5
MF	GO:0016918	retinal binding	3/260	13/19943	0.0005687	0.018502485	0.01562196	Stra6/Akr1b10/Rbp1	3
MF	GO:0005501	retinoid binding	4/260	31/19943	0.00067327	0.020658819	0.01744259	Stra6/Akr1b10/Rbp1/Ptgds	4
MF	GO:0030246	carbohydrate binding	11/260	271/19943	0.0009015	0.023621422	0.01994397	Col20a1/Enpp3/Mrc1/Siglec1/Hk3/Clec4a	11
MF	GO:0050840	extracellular matrix binding	5/260	61/19943	0.00118903	0.028439058	0.02401158	Lrrc15/Spp1/Sdc1/Nid1/Eln	5
MF	GO:0019840	isoprenoid binding	4/260	37/19943	0.0013293	0.02966441	0.02504617	Stra6/Akr1b10/Rbp1/Ptgds	4
MF	GO:1901681	sulfur compound binding	10/260	250/19943	0.00170365	0.03515851	0.02968493	Col13a1/Ltbp2/Postn/Abi3bp/Prelp/Fn1/	10
MF	GO:0004745	retinol dehydrogenase activity	3/260	20/19943	0.00211901	0.040339034	0.03405893	Adh1/Adh6/Dhrs9	3
MF	GO:0004024	alcohol dehydrogenase activity, zinc-dep	2/260	6/19943	0.00245347	0.04334457	0.03659656	Adh1/Adh6	2
MF	GO:0032027	myosin light chain binding	2/260	6/19943	0.00245347	0.04334457	0.03659656	Iqgap3/Cxcr4	2
MF	GO:0005518	collagen binding	5/260	72/19943	0.00249067	0.043835759	0.03701128	Lrrc15/Srgn/Abi3bp/Nid1/Ecm2	5

Repressed genes_KEGG

ID	Description	GeneRatio	BgRatio	pvalue	p.adjust	qvalue	geneID	Count
rno04512	ECM-receptor interaction	9/92	74/6610	6.93784E-07	7.3541E-05	6.7187E-05	Col1a1/Col4a1/Col4a2/Spp1/Sdc1/Lama2/Fn1/Col	9
rno04974	Protein digestion and absorption	8/92	78/6610	1.09418E-05	0.00057992	0.00052981	Col1a1/Slc7a7/Col4a1/Col4a2/Col15a1/Col18a1/C	8
rno04610	Complement and coagulation cascades	6/92	69/6610	0.000365571	0.01121097	0.0102424	Plau/C1qc/Cfd/C5ar1/F5/C7	6
rno05146	Amoebiasis	7/92	99/6610	0.000423055	0.01121097	0.0102424	Col1a1/Col4a1/Col4a2/Lama2/Fn1/Serpinb1a/Col	7
rno05222	Small cell lung cancer	6/92	84/6610	0.001049059	0.02224005	0.02031862	Col4a1/Col4a2/Ptgs2/Lama2/Fn1/Col4a5	6

Activated genes_GO

ONTOLOGY	ID	Description	GeneRatio	BgRatio	pvalue	p.adjust	qvalue	geneID	Count
BP	GO:0006836	neurotransmitter transport	51/373	322/19943	2.17E-32	9.47E-30	7.3752E-30	Cplx1/Grm4/Rph3a/Nsf/Napb/Syt2/Cadps2/Drd2/Cadps/Ncs1/Syr	51
BP	GO:0099504	synaptic vesicle cycle	44/373	243/19943	1.46E-30	5.78E-28	4.5005E-28	Cplx1/Grm4/Rapgef4/Napb/Syt2/Cadps2/Drd2/Cadps/Ncs1/Syn1/	44
BP	GO:0007269	neurotransmitter secretion	41/373	204/19943	2.02E-30	6.77E-28	5.2716E-28	Cplx1/Grm4/Rph3a/Nsf/Napb/Syt2/Cadps2/Drd2/Cadps/Ncs1/Syr	41
BP	GO:0099643	signal release from synapse	41/373	205/19943	2.48E-30	7.73E-28	6.0185E-28	Cplx1/Grm4/Rph3a/Nsf/Napb/Syt2/Cadps2/Drd2/Cadps/Ncs1/Syr	41
BP	GO:0001505	regulation of neurotransmitter levels	53/373	402/19943	1.45E-29	3.32E-27	2.5819E-27	Cplx1/Grm4/Rph3a/Tph2/Nsf/Napb/Syt2/Cadps2/Drd2/Cadps/Nc	53
BP	GO:0042391	regulation of membrane potential	56/373	464/19943	3.31E-29	7.21E-27	5.6151E-27	Rapgef4/Fgf12/Gabrg2/Glra2/Gabra1/Scn8a/Cacng2/Gjd2/Oprd1/	56
BP	GO:0097479	synaptic vesicle localization	39/373	201/19943	2.31E-28	4.46E-26	3.4711E-26	Cplx1/Grm4/Napb/Syt2/Kif5a/Cadps2/Drd2/Cadps/Ncs1/Syn1/Rir	39
BP	GO:0099003	vesicle-mediated transport in synapse	43/373	262/19943	4.77E-28	8.66E-26	6.7395E-26	Cplx1/Grm4/Napb/Syt2/Kif5a/Hpca/Cadps2/Drd2/Cadps/Ncs1/Rir	43
BP	GO:0050808	synapse organization	54/373	470/19943	4.2E-27	6.53E-25	5.0866E-25	Cabp1/Rph3a/Rapgef4/Gabrg2/Glra2/Gabra1/Arhgap44/Nptx1/Lr	54
BP	GO:0048489	synaptic vesicle transport	37/373	190/19943	5.18E-27	7.52E-25	5.8526E-25	Cplx1/Grm4/Napb/Syt2/Kif5a/Cadps2/Drd2/Cadps/Ncs1/Rims1/Cl	37
BP	GO:0097480	establishment of synaptic vesicle localiza	37/373	190/19943	5.18E-27	7.52E-25	5.8526E-25	Cplx1/Grm4/Napb/Syt2/Kif5a/Cadps2/Drd2/Cadps/Ncs1/Rims1/Cl	37
BP	GO:0016079	synaptic vesicle exocytosis	34/373	153/19943	6.4E-27	8.99E-25	7.0017E-25	Cplx1/Grm4/Napb/Syt2/Cadps2/Drd2/Cadps/Ncs1/Rims1/Chrna4/	34
BP	GO:0046928	regulation of neurotransmitter secretion	33/373	148/19943	3.29E-26	4.1E-24	3.1936E-24	Cplx1/Grm4/Napb/Syt2/Drd2/Ncs1/Rims1/Chrna4/Stx1b/Vamp1/	33
BP	GO:0017156	calcium ion regulated exocytosis	36/373	190/19943	7.18E-26	8.24E-24	6.4137E-24	Cplx1/Grm4/Rapgef4/Napb/Syt2/Cadps2/Drd2/Cadps/Ncs1/Rims1	36
BP	GO:0034765	regulation of ion transmembrane transp	53/373	483/19943	1.22E-25	1.37E-23	1.0639E-23	Cabp1/Rph3a/Fgf12/Scn8a/Kcnj3/Cacng2/Thy1/Hpca/Drd2/Kcnab	53
BP	GO:0045055	regulated exocytosis	41/373	276/19943	4.85E-25	5.29E-23	4.1173E-23	Cplx1/Grm4/Rapgef4/Napb/Syt2/Cadps2/Drd2/Cadps/Ncs1/Rims1	41
BP	GO:0051588	regulation of neurotransmitter transport	35/373	190/19943	9.61E-25	9.97E-23	7.7646E-23	Cplx1/Grm4/Napb/Syt2/Drd2/Ncs1/Rims1/Chrna4/Stx1b/Vamp1/	35
BP	GO:0017158	regulation of calcium ion-dependent exo	32/373	151/19943	1.05E-24	1.06E-22	8.2622E-23	Cplx1/Grm4/Napb/Syt2/Drd2/Cadps/Ncs1/Rims1/Chrna4/Stx1b/V	32
BP	GO:1902803	regulation of synaptic vesicle transport	29/373	118/19943	1.84E-24	1.67E-22	1.2979E-22	Cplx1/Grm4/Napb/Syt2/Drd2/Ncs1/Rims1/Chrna4/Stx1b/Vamp1/	29
BP	GO:0051648	vesicle localization	41/373	288/19943	2.61E-24	2.32E-22	1.8091E-22	Cplx1/Grm4/Napb/Syt2/Kif5a/Cadps2/Drd2/Cadps/Ncs1/Dync1i1/	41
BP	GO:0098693	regulation of synaptic vesicle cycle	32/373	157/19943	3.81E-24	3.32E-22	2.586E-22	Cplx1/Grm4/Rapgef4/Napb/Syt2/Drd2/Ncs1/Syn1/Rims1/Chrna4/	32
BP	GO:2000300	regulation of synaptic vesicle exocytosis	28/373	114/19943	1.19E-23	1.02E-21	7.94E-22	Cplx1/Grm4/Napb/Syt2/Drd2/Ncs1/Rims1/Chrna4/Stx1b/Vamp1/	28
BP	GO:0006887	exocytosis	46/373	391/19943	1.35E-23	1.13E-21	8.7801E-22	Cplx1/Grm4/Rapgef4/Arhgap44/Nsf/Napb/Syt2/Vsnl1/Cadps2/Dr	46
BP	GO:0051650	establishment of vesicle localization	39/373	269/19943	1.84E-23	1.51E-21	1.1776E-21	Cplx1/Grm4/Napb/Syt2/Kif5a/Cadps2/Drd2/Cadps/Ncs1/Dync1i1/	39
BP	GO:0017157	regulation of exocytosis	37/373	263/19943	7.68E-22	5.77E-20	4.4914E-20	Cplx1/Grm4/Rapgef4/Nsf/Napb/Syt2/Vsnl1/Cadps2/Drd2/Cadps/	37
BP	GO:1903305	regulation of regulated secretory pathw	32/373	204/19943	1.72E-20	1.25E-18	9.744E-19	Cplx1/Grm4/Napb/Syt2/Drd2/Cadps/Ncs1/Rims1/Chrna4/Stx1b/V	32
BP	GO:0006813	potassium ion transport	33/373	244/19943	4.73E-19	3.27E-17	2.5484E-17	Nsf/Kcnj3/Slc24a2/Drd2/Kcnab3/Kcns2/Kcnab2/Slc12a5/Kcna2/Kc	33
BP	GO:0051656	establishment of organelle localization	45/373	489/19943	8.45E-19	5.75E-17	4.4795E-17	Cplx1/Grm4/Napb/Syt2/Kif5a/Cadps2/Drd2/Cadps/Nefh/Ncs1/Dy	45
BP	GO:0060078	regulation of postsynaptic membrane pc	26/373	155/19943	1.31E-17	8.54E-16	6.6471E-16	Gabrg2/Glra2/Gabra1/Gabra5/Rims1/Chrna4/Grin1/Rgs7bp/Glra1	26
BP	GO:0071804	cellular potassium ion transport	29/373	212/19943	5.16E-17	3.21E-15	2.5024E-15	Kcnj3/Kcnab3/Kcns2/Kcnab2/Slc12a5/Kcna2/Kcnc3/Atp1a3/Rgs7/	29
BP	GO:0071805	potassium ion transmembrane transport	29/373	212/19943	5.16E-17	3.21E-15	2.5024E-15	Kcnj3/Kcnab3/Kcns2/Kcnab2/Slc12a5/Kcna2/Kcnc3/Atp1a3/Rgs7/	29
BP	GO:0007416	synapse assembly	26/373	184/19943	1E-15	5.9E-14	4.5944E-14	Gabrg2/Glra2/Gabra1/Nptx1/Lrfn5/Drd2/Slitrk3/Clstn3/Grin1/Syn	26
BP	GO:0035418	protein localization to synapse	19/373	86/19943	1.62E-15	9.41E-14	7.329E-14	Cplx1/Rapgef4/Arhgap44/Nptx1/Kif5a/Cacng2/Clstn3/Grin1/Cacn	19
BP	GO:1904062	regulation of cation transmembrane trar	34/373	355/19943	5.95E-15	3.32E-13	2.5881E-13	Cabp1/Rph3a/Fgf12/Cacng2/Thy1/Hpca/Drd2/Chrna4/Kcns2/Kcna	34
BP	GO:0032386	regulation of intracellular transport	38/373	464/19943	2.24E-14	1.21E-12	9.3852E-13	Cplx1/Grm4/Cabp1/Arhgap44/Napb/Syt2/Hpca/Drd2/Nefh/Ncs1/	38
BP	GO:2001257	regulation of cation channel activity	24/373	175/19943	2.58E-14	1.37E-12	1.0686E-12	Cabp1/Rph3a/Fgf12/Cacng2/Hpca/Drd2/Chrna4/Kcns2/Cacng3/Ra	24
BP	GO:0050905	neuromuscular process	21/373	134/19943	7.47E-14	3.72E-12	2.894E-12	Fgf12/Rbfox1/Scn8a/Drd2/Kcnab2/Grin1/Glra1/Nefl/Atp2b2/Cam	21
BP	GO:0030534	adult behavior	24/373	189/19943	1.47E-13	7.1E-12	5.5298E-12	Fgf12/Gabrg2/Scn8a/Drd2/Oprd1/Chrna4/Grin1/Glra1/Crhbp/Atp	24
BP	GO:0001508	action potential	21/373	139/19943	1.58E-13	7.55E-12	5.8749E-12	Rapgef4/Fgf12/Scn8a/Gjd2/Chrna4/Glra1/Ryr2/Kcna2/Scn4b/Dpp	21
BP	GO:0032412	regulation of ion transmembrane transp	27/373	260/19943	5.97E-13	2.63E-11	2.0473E-11	Cabp1/Rph3a/Fgf12/Cacng2/Hpca/Drd2/Chrna4/Kcns2/Cacng3/Ra	27
BP	GO:0050890	cognition	31/373	351/19943	8.64E-13	3.73E-11	2.9014E-11	Grm4/Tafa2/Slc24a2/Drd2/Gabra5/Chrna4/Grin1/Ndrg4/Slc12a5/	31
BP	GO:0050806	positive regulation of synaptic transmiss	29/373	308/19943	9.8E-13	4.15E-11	3.2291E-11	Syt2/Cacng2/Slc24a2/Drd2/Rims1/Clstn3/Grin1/Cacng3/Car7/Calt	29
BP	GO:0140029	exocytic process	17/373	92/19943	1.15E-12	4.81E-11	3.7437E-11	Cplx1/Napb/Syt2/Cadps2/Cadps/Rims1/Rab3c/Dmtn/Stx1b/Vamp	17
BP	GO:0022898	regulation of transmembrane transporte	27/373	268/19943	1.23E-12	5.12E-11	3.9877E-11	Cabp1/Rph3a/Fgf12/Cacng2/Hpca/Drd2/Chrna4/Kcns2/Cacng3/Ra	27
BP	GO:1903539	protein localization to postsynaptic mem	14/373	56/19943	1.43E-12	5.89E-11	4.5869E-11	Cplx1/Rapgef4/Arhgap44/Nptx1/Cacng2/Grin1/Cacng3/Stx1b/Grip	14
BP	GO:0099072	regulation of postsynaptic membrane ne	17/373	97/19943	2.83E-12	1.13E-10	8.7951E-11	Cplx1/Rapgef4/Arhgap44/Nptx1/Cacng2/Hpca/Cacng3/Stx1b/Pac	17
BP	GO:0032409	regulation of transporter activity	27/373	278/19943	2.94E-12	1.17E-10	9.0822E-11	Cabp1/Rph3a/Fgf12/Cacng2/Hpca/Drd2/Chrna4/Kcns2/Cacng3/Ra	27
BP	GO:0097120	receptor localization to synapse	15/373	71/19943	3.2E-12	1.25E-10	9.7045E-11	Cplx1/Rapgef4/Arhgap44/Nptx1/Kif5a/Cacng2/Cacng3/Stx1b/Grip	15
BP	GO:0007611	learning or memory	28/373	314/19943	9.1E-12	3.48E-10	2.7068E-10	Grm4/Tafa2/Slc24a2/Drd2/Gabra5/Grin1/Ndrg4/Slc12a5/Atp1a3/	28
BP	GO:0016082	synaptic vesicle priming	10/373	25/19943	1.18E-11	4.48E-10	3.4903E-10	Napb/Cadps2/Cadps/Rims1/Stx1b/Vamp1/Rab3a/Rims2/Erc2/Unc	10
BP	GO:0019226	transmission of nerve impulse	16/373	93/19943	1.67E-11	6.29E-10	4.8985E-10	Rapgef4/Fgf12/Scn8a/Cacng2/Cacng3/Glra1/Kcna2/Dpp6/Scn1a/	16
BP	GO:0048167	regulation of synaptic plasticity	28/373	327/19943	2.39E-11	8.9E-10	6.9284E-10	Cabp1/Slc24a2/Drd2/Rims1/Grin1/Rasgrf1/Calb2/Syt12/Rab3a/At	28
BP	GO:0051932	synaptic transmission, GABAergic	13/373	56/19943	2.54E-11	9.4E-10	7.3162E-10	Phf24/Grm4/Gabrg2/Gabra1/Drd2/Gabra5/Clstn3/Cntnap4/Car7/	13
BP	GO:1990778	protein localization to cell periphery	29/373	356/19943	3.5E-11	1.27E-09	9.8841E-10	Cplx1/Rapgef4/Arhgap44/Nptx1/Nsf/Cacng2/Rab3c/Grin1/Cacng3	29
BP	GO:0035637	multicellular organismal signaling	19/373	147/19943	3.87E-11	1.39E-09	1.0843E-09	Rapgef4/Fgf12/Scn8a/Cacng2/Cacng3/Glra1/Ryr2/Kcna2/Scn4b/D	19
BP	GO:0010959	regulation of metal ion transport	32/373	433/19943	4.17E-11	1.49E-09	1.1606E-09	Cabp1/Fgf12/Thy1/Hpca/Drd2/Oprd1/Kcns2/Kcnab2/Grin1/Hecw	32
BP	GO:0019228	neuronal action potential	12/373	48/19943	5.84E-11	2.04E-09	1.5854E-09	Rapgef4/Fgf12/Scn8a/Kcna2/Dpp6/Scn1a/Kcna1/Scn2a/Kcnma1/	12
BP	GO:0007626	locomotory behavior	24/373	250/19943	6.28E-11	2.16E-09	1.678E-09	Fgf12/Scn8a/Drd2/Oprd1/Chrna4/Grin1/Glra1/Grm1/Atp1a3/Sez	24
BP	GO:0051668	localization within membrane	21/373	189/19943	6.61E-11	2.25E-09	1.7515E-09	Cplx1/Rapgef4/Arhgap44/Nptx1/Kif5a/Cacng2/Thy1/Hpca/Cacng3	21
BP	GO:0007215	glutamate receptor signaling pathway	16/373	103/19943	8.33E-11	2.79E-09	2.1718E-09	Grm4/Rph3a/Cacng2/Grin1/Cacng3/Rasgrf1/Grm1/Crhbp/Atp1a3	16
BP	GO:0006816	calcium ion transport	32/373	446/19943	8.94E-11	2.95E-09	2.2968E-09	Cabp1/Cacng2/Thy1/Hpca/Slc24a2/Drd2/Ncs1/Oprd1/Chrna4/Gri	32
BP	GO:0099565	chemical synaptic transmission, postsyn	17/373	120/19943	9.61E-11	3.15E-09	2.4502E-09	Glra2/Rims1/Chrna4/Grin1/Glra1/Stx1b/Mapk8ip2/Cbln1/Grip2/R	17
BP	GO:0007613	memory	19/373	156/19943	1.1E-10	3.59E-09	2.795E-09	Tafa2/Slc24a2/Drd2/Grin1/Atp1a3/Jph3/Adcy1/Hrh3/Scn2a/Grin2	19
BP	GO:0031644	regulation of neurological system proces	20/373	177/19943	1.4E-10	4.51E-09	3.5129E-09	Rapgef4/Fgf12/Nptx1/Oprd1/Rims1/Chrna4/Grin1/Glra1/Grm1/St	20
BP	GO:0007612	learning	20/373	179/19943	1.71E-10	5.45E-09	4.2433E-09	Grm4/Tafa2/Slc24a2/Drd2/Gabra5/Grin1/Ndrg4/Slc12a5/Atp1a3/	20
BP	GO:0015837	amine transport	17/373	127/19943	2.39E-10	7.56E-09	5.8872E-09	Syt2/Drd2/Chrna4/Grm1/Kcna2/Syt12/Htr2c/Nat8l/Chga/Pcp4/Dc	17
BP	GO:0070588	calcium ion transmembrane transport	26/373	314/19943	2.62E-10	8.23E-09	6.4042E-09	Cabp1/Cacng2/Thy1/Hpca/Slc24a2/Drd2/Ncs1/Grin1/Cacng3/Ryr	26
BP	GO:0060079	excitatory postsynaptic potential	16/373	111/19943	2.64E-10	8.23E-09	6.4042E-09	Glra2/Rims1/Chrna4/Grin1/Glra1/Stx1b/Mapk8ip2/Cbln1/Grip2/R	16
BP	GO:0072511	divalent inorganic cation transport	33/373	497/19943	3.37E-10	1.03E-08	8.054E-09	Cabp1/Cacng2/Thy1/Hpca/Slc24a2/Drd2/Ncs1/Oprd1/Chrna4/Gri	33
BP	GO:0099601	regulation of neurotransmitter receptor	13/373	68/19943	3.45E-10	1.05E-08	8.1833E-09	Rph3a/Nptx1/Lypd6b/Lynx1/Cacng2/Chrna4/Cacng3/Rasgrf1/Crhl	13
BP	GO:0019932	second-messenger-mediated signaling	31/373	455/19943	6.35E-10	1.86E-08	1.4467E-08	Rapgef4/Lgr5/Vsnl1/Camk1g/Hpca/Drd2/Gpr3/Pex5l/Grin1/Dmtn	31
BP	GO:0051480	regulation of cytosolic calcium ion conce	29/373	406/19943	7.84E-10	2.28E-08	1.7723E-08	Trhr/Thy1/Drd2/Ncs1/Gpr3/Hcrtr2/Grin1/Hcrtr1/Grm1/Calb2/Ryr	29
BP	GO:0070838	divalent metal ion transport	32/373	494/19943	1.17E-09	3.37E-08	2.6232E-08	Cabp1/Cacng2/Thy1/Hpca/Slc24a2/Drd2/Ncs1/Oprd1/Chrna4/Gri	32
BP	GO:0051952	regulation of amine transport	16/373	123/19943	1.25E-09	3.59E-08	2.792E-08	Syt2/Drd2/Chrna4/Grm1/Kcna2/Syt12/Htr2c/Nat8l/Chga/Pcp4/Hr	16
BP	GO:0050432	catecholamine secretion	13/373	78/19943	2.05E-09	5.8E-08	4.5122E-08	Syt2/Drd2/Cadps/Chrna4/Kcna2/Syt12/Chga/Pcp4/Hrh3/Htr2a/Ch	13
BP	GO:0031503	protein-containing complex localization	22/373	250/19943	2.08E-09	5.85E-08	4.5549E-08	Cplx1/Rapgef4/Arhgap44/Nptx1/Kif5a/Cacng2/Hpca/Cacng3/Stx1	22
BP	GO:0051937	catecholamine transport	14/373	94/19943	2.26E-09	6.32E-08	4.9194E-08	Syt2/Drd2/Cadps/Chrna4/Kcna2/Syt12/Nat8l/Chga/Pcp4/Hrh3/Ht	14
BP	GO:0043266	regulation of potassium ion transport	15/373	113/19943	3.11E-09	8.63E-08	6.7213E-08	Drd2/Kcns2/Kcnab2/Rgs7/Dpp6/Kcnip4/Kcnc1/Kcnab1/Kcna1/Dpp	15
BP	GO:0098815	modulation of excitatory postsynaptic pc	11/373	53/19943	3.19E-09	8.8E-08	6.8529E-08	Rims1/Chrna4/Grin1/Stx1b/Cbln1/Grip2/Rims2/Chrna7/Neto1/Ce	11
BP	GO:0050803	regulation of synapse structure or activit	23/373	280/19943	3.38E-09	9.26E-08	7.2107E-08	Rapgef4/Arhgap44/Nptx1/Lrfn5/Drd2/Slitrk3/Clstn3/Cntnap4/Grir	23
BP	GO:0043270	positive regulation of ion transport	25/373	333/19943	4.4E-09	1.18E-07	9.2167E-08	Fgf12/Cacng2/Thy1/Drd2/Chrna4/Grin1/Cacng3/Ryr2/Rgs7/Scn4b	25
BP	GO:0051899	membrane depolarization	13/373	83/19943	4.52E-09	1.2E-07	9.3428E-08	Fgf12/Scn8a/Cacng2/Chrna4/Grm1/Scn4b/Scn1a/Scn2a/Scn9a/Ch	13
BP	GO:0050433	regulation of catecholamine secretion	12/373	74/19943	1.19E-08	3.03E-07	2.3578E-07	Syt2/Drd2/Chrna4/Kcna2/Syt12/Chga/Pcp4/Hrh3/Htr2a/Chrna7/C	12
BP	GO:0016358	dendrite development	23/373	301/19943	1.33E-08	3.38E-07	2.6288E-07	Rapgef4/Mcf2/Arhgap44/Grin1/Hecw1/Slc12a5/RGD1307443/Ma	23
BP	GO:1901379	regulation of potassium ion transmembr	13/373	91/19943	1.43E-08	3.59E-07	2.7913E-07	Kcns2/Kcnab2/Rgs7/Dpp6/Kcnip4/Kcnc1/Kcnab1/Kcna1/Dpp10/Al	13
BP	GO:0008344	adult locomotory behavior	14/373	108/19943	1.43E-08	3.59E-07	2.7913E-07	Fgf12/Scn8a/Drd2/Oprd1/Grin1/Glra1/Atp1a3/Sez6l2/Scn1a/Kcnn	14
BP	GO:0015844	monoamine transport	14/373	110/19943	1.82E-08	4.53E-07	3.5275E-07	Syt2/Drd2/Cadps/Chrna4/Kcna2/Syt12/Nat8l/Chga/Pcp4/Hrh3/Ht	14
BP	GO:0048168	regulation of neuronal synaptic plasticity	12/373	78/19943	2.2E-08	5.45E-07	4.2394E-07	Drd2/Rims1/Grin1/Rasgrf1/Rab3a/Camk2b/Syp/Camk2a/Jph3/Gri	12
BP	GO:0007214	gamma-aminobutyric acid signaling path	8/373	27/19943	2.26E-08	5.57E-07	4.3338E-07	Phf24/Gabrg2/Gabra1/Gabra5/Gabra3/Gabbr2/Htr4/Gabrb3	8
BP	GO:0019233	sensory perception of pain	16/373	154/19943	3.33E-08	8.16E-07	6.3554E-07	Phf24/Oprd1/Chrna4/Grin1/Grm1/Kcna2/Htr2c/Scn1a/Kcna1/Cac	16
BP	GO:0031630	regulation of synaptic vesicle fusion to p	6/373	12/19943	3.46E-08	8.41E-07	6.5498E-07	Cplx1/Syt2/Rims1/Vamp1/Rab3a/Syt7	6
BP	GO:0050807	regulation of synapse organization	21/373	270/19943	4.25E-08	1.03E-06	7.9801E-07	Rapgef4/Arhgap44/Nptx1/Lrfn5/Drd2/Slitrk3/Clstn3/Cntnap4/Grir	21
BP	GO:0099632	protein transport within plasma membra	8/373	30/19943	5.68E-08	1.34E-06	1.0408E-06	Cplx1/Arhgap44/Cacng2/Cacng3/Stx1b/Grip2/Mapk10/Lrrc7	8
BP	GO:0099637	neurotransmitter receptor transport	8/373	30/19943	5.68E-08	1.34E-06	1.0408E-06	Cplx1/Arhgap44/Cacng2/Cacng3/Stx1b/Grip2/Mapk10/Lrrc7	8
BP	GO:0006814	sodium ion transport	18/373	208/19943	8E-08	1.86E-06	1.4509E-06	Fgf12/Scn8a/Slc24a2/Drd2/Slc5a7/Hecw1/Slc17a6/Atp1a3/Scn4b/	18
BP	GO:1900449	regulation of glutamate receptor signalir	10/373	57/19943	9.18E-08	2.12E-06	1.6472E-06	Rph3a/Cacng2/Cacng3/Rasgrf1/Crhbp/Mapk8ip2/Necab2/Neto1/	10
BP	GO:0035249	synaptic transmission, glutamatergic	13/373	107/19943	1.03E-07	2.37E-06	1.8432E-06	Grm4/Napb/Cacng2/Drd2/Clstn3/Grin1/Cacng3/Grm1/Slc17a6/M	13
BP	GO:0007631	feeding behavior	14/373	128/19943	1.27E-07	2.9E-06	2.2575E-06	Drd2/Oprd1/Hcrtr2/Grin1/Hcrtr1/Htr2c/Atp8a2/Mrap2/Hrh3/Sgip	14
BP	GO:0035725	sodium ion transmembrane transport	14/373	129/19943	1.4E-07	3.18E-06	2.4769E-06	Fgf12/Scn8a/Slc24a2/Hecw1/Atp1a3/Scn4b/Scn1a/Scn2a/Scn9a/	14

BP	GO:0031629	synaptic vesicle fusion to presynaptic act	7/373	23/19943	1.43E-07	3.24E-06	2.5198E-06	Cplx1/Syt2/Rims1/Stx1b/Vamp1/Rab3a/Syt7	7
BP	GO:0014046	dopamine secretion	9/373	46/19943	1.52E-07	3.4E-06	2.6469E-06	Syt2/Drd2/Chrna4/Kcna2/Syt12/Pcp4/Htr2a/Chrnb2/Syt7	9
BP	GO:0014059	regulation of dopamine secretion	9/373	46/19943	1.52E-07	3.4E-06	2.6469E-06	Syt2/Drd2/Chrna4/Kcna2/Syt12/Pcp4/Htr2a/Chrnb2/Syt7	9
BP	GO:0015696	ammonium transport	14/373	131/19943	1.7E-07	3.78E-06	2.9436E-06	Syt2/Drd2/Slc5a7/Chrna4/Slc12a5/Kcna2/Syt12/Htr2c/Nat8l/Pcp4	14
BP	GO:0015872	dopamine transport	10/373	61/19943	1.79E-07	3.97E-06	3.0879E-06	Syt2/Drd2/Chrna4/Kcna2/Syt12/Nat8l/Pcp4/Htr2a/Chrnb2/Syt7	10
BP	GO:0099500	vesicle fusion to plasma membrane	7/373	24/19943	1.99E-07	4.38E-06	3.4121E-06	Cplx1/Syt2/Rims1/Stx1b/Vamp1/Rab3a/Syt7	7
BP	GO:0048278	vesicle docking	10/373	63/19943	2.46E-07	5.36E-06	4.17E-06	Nsf/Rims1/Rab3c/Ndrg4/Stx1b/Rab3a/Camk2a/Rims2/Unc13c/Sp	10
BP	GO:0051592	response to calcium ion	16/373	178/19943	2.52E-07	5.46E-06	4.2539E-06	Tph2/Syt2/Cacng2/Hpca/Grin1/Dmtn/Ryr2/Gpld1/Crhbp/Syt12/C	16
BP	GO:0016081	synaptic vesicle docking	6/373	16/19943	2.81E-07	6.06E-06	4.7208E-06	Rims1/Stx1b/Camk2a/Rims2/Unc13c/Sptbn2	6
BP	GO:0031646	positive regulation of neurological syster	11/373	81/19943	3.18E-07	6.83E-06	5.3134E-06	Rapgef4/Rims1/Grin1/Stx1b/Grip2/Rims2/Nrg1/Chrna7/Neto1/Op	11
BP	GO:2000463	positive regulation of excitatory postsyn	8/373	37/19943	3.34E-07	7.1E-06	5.5261E-06	Rims1/Grin1/Stx1b/Grip2/Rims2/Chrna7/Neto1/Shank1	8
BP	GO:0099173	postsynapse organization	17/373	205/19943	3.39E-07	7.16E-06	5.5752E-06	Rph3a/Rapgef4/Arhgap44/Nptx1/Grin1/Nefl/Ina/Zfp804a/Camk2b	17
BP	GO:0032594	protein transport within lipid bilayer	8/373	39/19943	5.15E-07	1.05E-05	8.2031E-06	Cplx1/Arhgap44/Cacng2/Cacng3/Stx1b/Grip2/Mapk10/Lrrc7	8
BP	GO:0060052	neurofilament cytoskeleton organizatior	5/373	10/19943	5.2E-07	1.06E-05	8.2416E-06	Nefh/Nefl/Nefm/Ina/Atp8a2	5
BP	GO:0060359	response to ammonium ion	16/373	189/19943	5.69E-07	1.15E-05	8.9817E-06	Gabrg2/Gabra1/Drd2/Nefh/Chrna4/Grin1/Crhbp/Chrm2/Tac3/Hrh	16
BP	GO:0140056	organelle localization by membrane teth	10/373	72/19943	8.88E-07	1.78E-05	1.3824E-05	Nsf/Rims1/Rab3c/Ndrg4/Stx1b/Rab3a/Camk2a/Rims2/Unc13c/Sp	10
BP	GO:0099505	regulation of presynaptic membrane pot	5/373	11/19943	9.38E-07	1.86E-05	1.4472E-05	Grin1/Kcnc1/Kcna1/Scn2a/Kcnma1	5
BP	GO:0008306	associative learning	12/373	109/19943	9.43E-07	1.86E-05	1.4483E-05	Tafa2/Drd2/Gabra5/Grin1/Ndrg4/Atp1a3/Grin2b/Chrna7/Neto1/C	12
BP	GO:0099633	protein localization to postsynaptic spec	7/373	30/19943	1.06E-06	2.07E-05	1.6105E-05	Rapgef4/Nptx1/Cacng2/Cacng3/Camk2a/Dlg2/Adam22	7
BP	GO:0099645	neurotransmitter receptor localization tc	7/373	30/19943	1.06E-06	2.07E-05	1.6105E-05	Rapgef4/Nptx1/Cacng2/Cacng3/Camk2a/Dlg2/Adam22	7
BP	GO:0086010	membrane depolarization during action	7/373	31/19943	1.35E-06	2.59E-05	2.0199E-05	Scn8a/Scn4b/Scn1a/Scn2a/Scn9a/Cacna2d1/Cacna1i	7
BP	GO:0019722	calcium-mediated signaling	17/373	227/19943	1.41E-06	2.7E-05	2.0999E-05	Vsnl1/Camk1g/Hpca/Grin1/Dmtn/Grm1/Ryr2/Rit2/Tenm2/Necab	17
BP	GO:0007628	adult walking behavior	8/373	45/19943	1.64E-06	3.1E-05	2.4134E-05	Scn8a/Drd2/Glra1/Scn1a/Kcnma1/Chat/Klhl1/Cend1	8
BP	GO:0001964	startle response	7/373	32/19943	1.7E-06	3.21E-05	2.5001E-05	Drd2/Grin1/Glra1/Kcna1/Kcnh1/Grin2b/Nrg1	7
BP	GO:0006904	vesicle docking involved in exocytosis	8/373	46/19943	1.95E-06	3.63E-05	2.8251E-05	Rims1/Rab3c/Stx1b/Rab3a/Camk2a/Rims2/Unc13c/Sptbn2	8
BP	GO:0090659	walking behavior	8/373	47/19943	2.31E-06	4.25E-05	3.3072E-05	Scn8a/Drd2/Glra1/Scn1a/Kcnma1/Chat/Klhl1/Cend1	8
BP	GO:0098969	neurotransmitter receptor transport to p	6/373	22/19943	2.38E-06	4.34E-05	3.3804E-05	Cplx1/Arhgap44/Stx1b/Grip2/Mapk10/Lrrc7	6
BP	GO:0015893	drug transport	17/373	236/19943	2.4E-06	4.36E-05	3.3975E-05	Syt2/Drd2/Chrna4/Slc32a1/Slc17a6/Kcna2/Syt12/Htr2c/Slc6a5/Na	17
BP	GO:0007271	synaptic transmission, cholinergic	7/373	34/19943	2.63E-06	4.72E-05	3.6782E-05	Lynx1/Slc5a7/Chrna4/Htr2c/Camk2b/Chrna7/Chrnb2	7
BP	GO:0071248	cellular response to metal ion	17/373	238/19943	2.7E-06	4.81E-05	3.7477E-05	Glra2/Nptx1/Tph2/Syt2/Hpca/Grin1/Dmtn/Glra1/Gpld1/Crhbp/Sy	17
BP	GO:0022406	membrane docking	10/373	82/19943	3E-06	5.3E-05	4.1242E-05	Nsf/Rims1/Rab3c/Ndrg4/Stx1b/Rab3a/Camk2a/Rims2/Unc13c/Sp	10
BP	GO:0098877	neurotransmitter receptor transport to p	6/373	23/19943	3.17E-06	5.55E-05	4.3209E-05	Cplx1/Arhgap44/Stx1b/Grip2/Mapk10/Lrrc7	6
BP	GO:1903540	establishment of protein localization to p	6/373	23/19943	3.17E-06	5.55E-05	4.3209E-05	Cplx1/Arhgap44/Stx1b/Grip2/Mapk10/Lrrc7	6
BP	GO:0015850	organic hydroxy compound transport	18/373	270/19943	3.65E-06	6.34E-05	4.9339E-05	Syt2/Drd2/Cadps/Chrna4/Kcna2/Syt12/Nat8l/Chga/Pcp4/Hrh3/Ab	18
BP	GO:0097164	ammonium ion metabolic process	14/373	172/19943	4.6E-06	7.89E-05	6.1419E-05	Tph2/Drd2/Slc5a7/Asah2/Gpld1/Sncb/Atp2b2/Htr2c/Sgpp2/Ddc/C	14
BP	GO:0050885	neuromuscular process controlling balan	9/373	69/19943	5.36E-06	9.12E-05	7.102E-05	Rbfox1/Nefl/Atp2b2/Camk2b/Jph3/Kcnma1/Camta1/Shank1/Pou4	9
BP	GO:0060402	calcium ion transport into cytosol	14/373	175/19943	5.62E-06	9.5E-05	7.3938E-05	Thy1/Drd2/Grin1/Ryr2/Htr2c/Plch2/Jph3/Akap6/Grin2b/Htr2a/Prl	14
BP	GO:0048169	regulation of long-term neuronal synapti	7/373	38/19943	5.8E-06	9.75E-05	7.5911E-05	Drd2/Rims1/Grin1/Camk2b/Syp/Grin2b/Neto1	7
BP	GO:0051590	positive regulation of neurotransmitter t	8/373	53/19943	5.91E-06	9.9E-05	7.7058E-05	Syt2/Drd2/Rims1/Stx1b/Htr2c/Nat8l/Cacna1b/Syt7	8
BP	GO:0007409	axonogenesis	24/373	465/19943	8.1E-06	0.000134	0.00010454	Nptx1/Kif5a/Cntn4/Thy1/Drd2/Nefh/Slitrk3/Grin1/Nefl/Nefm/Rab	24
BP	GO:0007187	G protein-coupled receptor signaling pat	16/373	237/19943	1.08E-05	0.000178	0.00013894	Grm4/Lgr5/Drd2/Gpr3/Oprd1/Rit2/Htr2c/Chrm2/Chga/Mrap2/Gn	16
BP	GO:0032225	regulation of synaptic transmission, dopa	5/373	17/19943	1.15E-05	0.000188	0.00014613	Drd2/Cntnap4/Nat8l/Chrna7/Chrnb2	5
BP	GO:1902805	positive regulation of synaptic vesicle tra	5/373	17/19943	1.15E-05	0.000188	0.00014613	Syt2/Rims1/Cacna1b/Map2/Syt7	5
BP	GO:0007204	positive regulation of cytosolic calcium ic	20/373	353/19943	1.22E-05	0.000199	0.00015469	Trhr/Thy1/Drd2/Grin1/Grm1/Ryr2/Htr2c/Plch2/Jph3/Hrh3/Cacna1	20
BP	GO:0060401	cytosolic calcium ion transport	14/373	188/19943	1.28E-05	0.000207	0.00016138	Thy1/Drd2/Grin1/Ryr2/Htr2c/Plch2/Jph3/Akap6/Grin2b/Htr2a/Prl	14
BP	GO:0071242	cellular response to ammonium ion	9/373	77/19943	1.34E-05	0.000216	0.00016808	Gabrg2/Gabra1/Chrna4/Crhbp/Chrm2/Hrh3/Chrna7/Chrnb2/Gabr	9
BP	GO:0019933	cAMP-mediated signaling	14/373	189/19943	1.36E-05	0.000219	0.00017011	Rapgef4/Lgr5/Drd2/Gpr3/Pex5l/Rit2/Gpr61/Chga/Mrap2/Gnas/Ac	14
BP	GO:0035095	behavioral response to nicotine	4/373	9/19943	1.41E-05	0.000225	0.00017506	Chrna4/Htr2c/Chrna7/Chrnb2	4
BP	GO:0048791	calcium ion-regulated exocytosis of neur	5/373	18/19943	1.56E-05	0.000248	0.00019271	Syt2/Rims1/Stx1b/Rims2/Syt7	5
BP	GO:0051963	regulation of synapse assembly	11/373	120/19943	1.59E-05	0.00025	0.00019467	Nptx1/Lrfn5/Slitrk3/Clstn3/Grin1/Syndig1/Cbln1/Lingo4/Slit1/Chr	11
BP	GO:2001023	regulation of response to drug	11/373	120/19943	1.59E-05	0.00025	0.00019467	Syt2/Drd2/Chrna4/Kcna2/Syt12/Htr2c/Nat8l/Pcp4/Htr2a/Chrnb2/	11
BP	GO:1902476	chloride transmembrane transport	9/373	79/19943	1.65E-05	0.000259	0.00020154	Gabrg2/Glra2/Gabra1/Gabra5/Glra1/Slc12a5/Gabra3/Slc12a3/Gal	9
BP	GO:0019935	cyclic-nucleotide-mediated signaling	15/373	221/19943	1.91E-05	0.000298	0.00023211	Rapgef4/Lgr5/Drd2/Gpr3/Pex5l/Rit2/Gpr61/Htr2c/Chga/Mrap2/G	15
BP	GO:0106027	neuron projection organization	10/373	101/19943	1.97E-05	0.000307	0.00023861	Rph3a/Arhgap44/Grin1/Atp1a3/Zfp804a/Camk2b/Grin2b/Chrna7/	10
BP	GO:0001956	positive regulation of neurotransmitter s	6/373	31/19943	2.04E-05	0.000316	0.0002461	Syt2/Rims1/Stx1b/Htr2c/Cacna1b/Syt7	6
BP	GO:0060081	membrane hyperpolarization	5/373	19/19943	2.09E-05	0.00032	0.00024894	Cacng2/Kcnma1/Kcnq2/Trpc5/Hcn1	5
BP	GO:0050773	regulation of dendrite development	14/373	198/19943	2.29E-05	0.000349	0.00027192	Rapgef4/Arhgap44/Grin1/Hecw1/RGD1307443/Camk2b/Pacsin1/	14
BP	GO:0010807	regulation of synaptic vesicle priming	4/373	10/19943	2.31E-05	0.00035	0.00027249	Napb/Rims1/Stx1b/Rab3a	4
BP	GO:0061669	spontaneous neurotransmitter secretion	4/373	10/19943	2.31E-05	0.00035	0.00027249	Rph3a/Rims1/Stx1b/Rims2	4
BP	GO:0006821	chloride transport	10/373	103/19943	2.34E-05	0.000353	0.00027464	Gabrg2/Glra2/Gabra1/Gabra5/Car7/Glra1/Slc12a5/Gabra3/Slc12a	10
BP	GO:1901016	regulation of potassium ion transmembr	8/373	64/19943	2.47E-05	0.00037	0.0002879	Kcns2/Kcnc1/Kcnab1/Kcna1/Akap6/Neto1/Neto2/Kcng1	8
BP	GO:0045956	positive regulation of calcium ion-depen	6/373	32/19943	2.47E-05	0.00037	0.0002879	Syt2/Cadps/Rims1/Cacna1b/Syt7/Cacna1i	6
BP	GO:0010976	positive regulation of neuron projection	20/373	371/19943	2.5E-05	0.000371	0.00028852	Syt2/Serpini1/Rims1/Ndrg4/Nefl/Rit2/Zfp804a/Camk2b/Pacsin1/P	20
BP	GO:0097553	calcium ion transmembrane import into	12/373	151/19943	2.79E-05	0.000411	0.00032025	Thy1/Drd2/Grin1/Ryr2/Htr2c/Plch2/Jph3/Akap6/Grin2b/Htr2a/Prl	12
BP	GO:0045921	positive regulation of exocytosis	10/373	106/19943	3.01E-05	0.00044	0.00034224	Syt2/Vsnl1/Cadps2/Cadps/Ncs1/Rims1/Rab3a/Cacna1b/Syt7/Cacr	10
BP	GO:0072659	protein localization to plasma membrane	17/373	288/19943	3.28E-05	0.000478	0.0003721	Arhgap44/Nsf/Rab3c/Grm1/Epb41l3/Ank1/Rab3a/Dpp6/Kcnip4/C	17
BP	GO:0048813	dendrite morphogenesis	13/373	180/19943	3.59E-05	0.000513	0.00039917	Arhgap44/Grin1/Hecw1/Mapk8ip2/Camk2b/Camk2a/Cit/Chrna7/	13
BP	GO:0050954	sensory perception of mechanical stimul	16/373	262/19943	3.71E-05	0.000528	0.00041118	Phf24/Scn8a/Gabra5/Rab3a/Atp2b2/Scn1a/Kcna1/Srrm4/Kcnma1	16
BP	GO:0031346	positive regulation of cell projection orga	23/373	478/19943	3.77E-05	0.000535	0.00041649	Syt2/Serpini1/Rims1/Grin1/Ndrg4/Nefl/Rit2/Tenm2/Zfp804a/Cam	23
BP	GO:0010970	transport along microtubule	12/373	156/19943	3.86E-05	0.000544	0.00042342	Kif5a/Nefh/Dync1i1/Nefl/Nefm/Kif5c/Rasgrp1/Map2/Map1b/Dlg2	12
BP	GO:0007188	adenylate cyclase-modulating G protein-	14/373	208/19943	3.95E-05	0.000554	0.00043151	Grm4/Lgr5/Drd2/Gpr3/Oprd1/Rit2/Chrm2/Chga/Mrap2/Gnas/Ad	14
BP	GO:0071277	cellular response to calcium ion	9/373	88/19943	3.96E-05	0.000554	0.00043151	Syt2/Hpca/Dmtn/Gpld1/Crhbp/Syt12/Cpne4/Kcnh1/Syt7	9
BP	GO:0099111	microtubule-based transport	12/373	157/19943	4.11E-05	0.000574	0.00044654	Kif5a/Nefh/Dync1i1/Nefl/Nefm/Kif5c/Rasgrp1/Map2/Map1b/Dlg2	12
BP	GO:0031338	regulation of vesicle fusion	6/373	35/19943	4.22E-05	0.000586	0.00045582	Cplx1/Syt2/Rims1/Vamp1/Rab3a/Syt7	6
BP	GO:0015812	gamma-aminobutyric acid transport	5/373	22/19943	4.51E-05	0.000617	0.00048009	Grm4/Slc32a1/Htr2c/Slc6a11/Hrh3	5
BP	GO:0050966	detection of mechanical stimulus involve	5/373	22/19943	4.51E-05	0.000617	0.00048009	Phf24/Scn1a/Kcna1/Grin2b/Htr2a	5
BP	GO:0060080	inhibitory postsynaptic potential	5/373	22/19943	4.51E-05	0.000617	0.00048009	Rims1/Chrna4/Glra1/Rims2/Gabrb3	5
BP	GO:0007622	rhythmic behavior	8/373	70/19943	4.77E-05	0.00065	0.00050587	Drd2/Hcrtr2/Kcna2/Mapk10/Kcnma1/Oprl1/Chrnb2/Chat	8
BP	GO:0051954	positive regulation of amine transport	7/373	52/19943	4.91E-05	0.000666	0.00051859	Drd2/Chrna4/Htr2c/Nat8l/Pcp4/Grin2b/Chrnb2	7
BP	GO:0001963	synaptic transmission, dopaminergic	6/373	36/19943	4.98E-05	0.000674	0.00052506	Drd2/Cntnap4/Crhbp/Nat8l/Chrna7/Chrnb2	6
BP	GO:0097061	dendritic spine organization	9/373	93/19943	6.14E-05	0.000823	0.00064048	Rph3a/Arhgap44/Grin1/Zfp804a/Camk2b/Grin2b/Chrna7/Shank1/	9
BP	GO:1903532	positive regulation of secretion by cell	23/373	495/19943	6.42E-05	0.000859	0.0006685	Rapgef4/Syt2/Vsnl1/Cadps2/Drd2/Cadps/Ncs1/Rims1/Chrna4/Pex	23
BP	GO:0045471	response to ethanol	15/373	246/19943	6.57E-05	0.000876	0.00068214	Glra2/Kif5a/Drd2/Grin1/Glra1/Crhbp/Rgs7/Cacna1b/Grin2b/Chrna	15
BP	GO:0006936	muscle contraction	17/373	306/19943	6.98E-05	0.000927	0.00072176	Drd2/Glra1/Ryr2/Atp1a3/Scn4b/Chrm2/Chga/Scn1a/Atp8a2/Grip	17
BP	GO:0099509	regulation of presynaptic cytosolic calciu	5/373	24/19943	7.07E-05	0.000933	0.00072635	Ncs1/Calb2/Atp2b3/Kcnh1/Cacna1b	5
BP	GO:0007200	phospholipase C-activating G protein-co	9/373	95/19943	7.25E-05	0.000954	0.00074242	Drd2/Oprd1/Hcrtr2/Grm1/Htr2c/Chga/Htr2a/Mlnr/Oprl1	9
BP	GO:0098742	cell-cell adhesion via plasma-membrane	14/373	220/19943	7.27E-05	0.000954	0.00074242	Lrfn5/Cntn4/Slitrk3/Clstn3/Cdh12/Tenm2/Cdh7/Cbln1/Ptprt/Nrg1	14
BP	GO:0034776	response to histamine	4/373	13/19943	7.53E-05	0.000977	0.00076057	Gabrg2/Gabra1/Drd2/Gabrb3	4
BP	GO:0098814	spontaneous synaptic transmission	4/373	13/19943	7.53E-05	0.000977	0.00076057	Rph3a/Rims1/Stx1b/Rims2	4
BP	GO:0099590	neurotransmitter receptor internalizatio	6/373	39/19943	7.96E-05	0.001027	0.00079919	Cacng2/Hpca/Cacng3/Pacsin1/Nrg1/Dnm3	6
BP	GO:0030705	cytoskeleton-dependent intracellular tra	12/373	169/19943	8.4E-05	0.001079	0.00084023	Kif5a/Nefh/Dync1i1/Nefl/Nefm/Kif5c/Rasgrp1/Map2/Map1b/Dlg2	12
BP	GO:0045666	positive regulation of neuron differentia	22/373	471/19943	8.57E-05	0.001095	0.00085276	Syt2/Serpini1/Rims1/Ndrg4/Nefl/Rit2/Zfp804a/Camk2b/Pacsin1/P	22
BP	GO:0071361	cellular response to ethanol	6/373	40/19943	9.22E-05	0.001172	0.00091204	Glra2/Kif5a/Glra1/Prkce/Sptbn2/L1cam	6
BP	GO:2001025	positive regulation of response to drug	6/373	40/19943	9.22E-05	0.001172	0.00091204	Drd2/Chrna4/Htr2c/Nat8l/Pcp4/Chrnb2	6
BP	GO:0071241	cellular response to inorganic substance	17/373	314/19943	9.56E-05	0.001212	0.00094326	Glra2/Nptx1/Tph2/Syt2/Hpca/Grin1/Dmtn/Glra1/Gpld1/Crhbp/Sy	17
BP	GO:1905475	regulation of protein localization to mem	13/373	199/19943	0.000101	0.001266	0.00098548	Rapgef4/Arhgap44/Cacng2/Hpca/Dmtn/Grm1/Dpp6/Camk2b/Grip	13
BP	GO:0099566	regulation of postsynaptic cytosolic calci	4/373	14/19943	0.000104	0.0013	0.0010119	Grm1/Atp2b2/Grin2b/Slc8a3	4
BP	GO:0051966	regulation of synaptic transmission, glut	8/373	78/19943	0.000104	0.0013	0.0010119	Grm4/Cacng2/Drd2/Cacng3/Grm1/Mapk8ip2/Htr2a/Shank1	8
BP	GO:0098661	inorganic anion transmembrane transpo	9/373	101/19943	0.000117	0.001451	0.00112964	Gabrg2/Glra2/Gabra1/Gabra5/Glra1/Slc12a5/Gabra3/Slc12a3/Gal	9
BP	GO:0007616	long-term memory	6/373	42/19943	0.000122	0.001506	0.0011727	Drd2/Grin1/Adcy1/Grin2b/Shank1/Camk4	6
BP	GO:0042417	dopamine metabolic process	6/373	42/19943	0.000122	0.001506	0.0011727	Drd2/Sncb/Htr2c/Ddc/Chrna7/Chrnb2	6

BP	GO:0099175	regulation of postsynapse organization	10/373	125/19943	0.000122	0.001506	0.0011727	Rapgef4/Arhgap44/Nptx1/Grin1/Zfp804a/Camk2b/Cbln1/Grip2/G	10
BP	GO:0021631	optic nerve morphogenesis	3/373	6/19943	0.000124	0.001512	0.00117741	Kcna2/Pax2/Chrnb2	3
BP	GO:0031632	positive regulation of synaptic vesicle fu:	3/373	6/19943	0.000124	0.001512	0.00117741	Syt2/Rims1/Syt7	3
BP	GO:1902630	regulation of membrane hyperpolarizati	3/373	6/19943	0.000124	0.001512	0.00117741	Kcnma1/Trpc5/Hcn1	3
BP	GO:0050805	negative regulation of synaptic transmis:	8/373	80/19943	0.000125	0.001512	0.00117741	Grm4/Rapgef4/Slc24a2/Drd2/Cbln1/Htr2a/Celf4/Grid2ip	8
BP	GO:0036465	synaptic vesicle recycling	8/373	81/19943	0.000136	0.001642	0.00127834	Syt2/Sncb/Stx1b/Rab3a/Pacsin1/Syp/Syt7/Dnm3	8
BP	GO:0051705	multi-organism behavior	8/373	82/19943	0.000148	0.001781	0.00138612	Grin1/Crhbp/Mapk8ip2/Grin2b/Shank1/Chrnb2/Nrxn3/Pou4f1	8
BP	GO:0095500	acetylcholine receptor signaling pathwa	5/373	28/19943	0.000154	0.001825	0.00142082	Chrna4/Chrm2/Hrh3/Chrna7/Chrnb2	5
BP	GO:1903831	signal transduction involved in cellular re	5/373	28/19943	0.000154	0.001825	0.00142082	Chrna4/Chrm2/Hrh3/Chrna7/Chrnb2	5
BP	GO:0050982	detection of mechanical stimulus	7/373	62/19943	0.000154	0.001825	0.00142082	Phf24/Ano3/Atp2b2/Scn1a/Kcna1/Grin2b/Htr2a	7
BP	GO:0031623	receptor internalization	10/373	129/19943	0.000159	0.001881	0.00146435	Nsf/Napb/Cacng2/Hpca/Drd2/Cacng3/Pacsin1/Necab2/Nrg1/Dnm	10
BP	GO:2000302	positive regulation of synaptic vesicle ex	4/373	16/19943	0.000183	0.002154	0.00167707	Syt2/Rims1/Cacna1b/Syt7	4
BP	GO:1901019	regulation of calcium ion transmembran	8/373	85/19943	0.000191	0.002235	0.00173978	Cabp1/Hpca/Drd2/Ryr2/Jph3/Akap6/Oprl1/Cacna2d1	8
BP	GO:0008277	regulation of G protein-coupled recepto	10/373	132/19943	0.000192	0.002242	0.00174504	Phf24/Drd2/Rgs7/Camk2b/Chga/Syp/Mrap2/Necab2/Oprl1/Tub	10
BP	GO:0007189	adenylate cyclase-activating G protein-c	10/373	133/19943	0.000204	0.002372	0.00184623	Lgr5/Drd2/Gpr3/Rit2/Chga/Mrap2/Gnas/Adcy1/Oprl1/Htr4	10
BP	GO:0043949	regulation of cAMP-mediated signaling	7/373	65/19943	0.000207	0.002397	0.00186633	Gpr3/Pex5l/Gpr61/Chga/Mrap2/Gnas/Oprl1	7
BP	GO:0032226	positive regulation of synaptic transmiss	3/373	7/19943	0.000215	0.002437	0.00189682	Drd2/Nat8l/Chrnb2	3
BP	GO:0045110	intermediate filament bundle assembly	3/373	7/19943	0.000215	0.002437	0.00189682	Nefh/Nefl/Nefm	3
BP	GO:0060012	synaptic transmission, glycinergic	3/373	7/19943	0.000215	0.002437	0.00189682	Glra2/Glra1/Slc6a5	3
BP	GO:0060024	rhythmic synaptic transmission	3/373	7/19943	0.000215	0.002437	0.00189682	Scn9a/Cacna2d2/Chat	3
BP	GO:0055075	potassium ion homeostasis	5/373	30/19943	0.000216	0.002437	0.00189682	Drd2/Slc12a5/Atp1a3/Kcnma1/Slc12a3	5
BP	GO:1905144	response to acetylcholine	5/373	30/19943	0.000216	0.002437	0.00189682	Chrna4/Chrm2/Hrh3/Chrna7/Chrnb2	5
BP	GO:1905145	cellular response to acetylcholine	5/373	30/19943	0.000216	0.002437	0.00189682	Chrna4/Chrm2/Hrh3/Chrna7/Chrnb2	5
BP	GO:0031345	negative regulation of cell projection org	13/373	215/19943	0.000217	0.002437	0.00189682	Thy1/Rit2/RGD1307443/Stx1b/Slit1/Cit/Grin2b/Map2/Pfn2/LOC1(	13
BP	GO:0072347	response to anesthetic	10/373	134/19943	0.000217	0.002437	0.00189682	Hpca/Drd2/Nefh/Grin1/Crhbp/Tac3/Grin2b/Htr2a/Prkce/Chrnb2	10
BP	GO:0001504	neurotransmitter uptake	6/373	47/19943	0.000231	0.002589	0.00201524	Drd2/Slc17a6/Slc6a5/Nat8l/Slc6a11/Sv2b	6
BP	GO:0031280	negative regulation of cyclase activity	4/373	17/19943	0.000236	0.00264	0.00205558	Hpca/Drd2/Hrh3/Akap6	4
BP	GO:0048259	regulation of receptor-mediated endocy	9/373	111/19943	0.00024	0.002677	0.00208397	Nsf/Napb/Hpca/Drd2/Pacsin1/Necab2/Sgip1/Nrg1/Aak1	9
BP	GO:0050974	detection of mechanical stimulus involve	6/373	48/19943	0.00026	0.00289	0.00224991	Phf24/Atp2b2/Scn1a/Kcna1/Grin2b/Htr2a	6
BP	GO:0048512	circadian behavior	7/373	68/19943	0.000275	0.003053	0.00237647	Drd2/Hcrtr2/Kcna2/Mapk10/Kcnma1/Oprl1/Chrnb2	7
BP	GO:0032535	regulation of cellular component size	19/373	410/19943	0.000285	0.003152	0.00245412	Nefl/Nefm/Spire2/Slc12a5/Atp2b2/Slit1/Cit/Kcnma1/Nrg1/Map2/	19
BP	GO:0099054	presynapse assembly	6/373	49/19943	0.000292	0.003217	0.00250476	Lrfn5/Slitrk3/Clstn3/Cbln1/Cntn5/Bsn	6
BP	GO:0007274	neuromuscular synaptic transmission	5/373	32/19943	0.000296	0.003258	0.0025363	Slc5a7/Chrna4/Rab3a/Chrnb2/Chat	5
BP	GO:0050884	neuromuscular process controlling postu	4/373	18/19943	0.000299	0.003278	0.00255172	Glra1/Scn1a/Atp8a2/Pou4f1	4
BP	GO:0008542	visual learning	7/373	69/19943	0.000302	0.003285	0.00255737	Tafa2/Drd2/Grin1/Ndrg4/Atp1a3/Neto1/Chrnb2	7
BP	GO:1903307	positive regulation of regulated secretor	7/373	69/19943	0.000302	0.003285	0.00255737	Syt2/Cadps/Rims1/Rab3a/Cacna1b/Syt7/Cacna1i	7
BP	GO:0006906	vesicle fusion	8/373	91/19943	0.000306	0.003317	0.00258218	Cplx1/Nsf/Syt2/Rims1/Stx1b/Vamp1/Rab3a/Syt7	8
BP	GO:0051924	regulation of calcium ion transport	15/373	283/19943	0.000306	0.003317	0.00258218	Cabp1/Thy1/Hpca/Drd2/Oprd1/Grin1/Ryr2/Atp2b2/Camk2a/Jph3,	15
BP	GO:0046958	nonassociative learning	3/373	8/19943	0.000339	0.003638	0.00283232	Mapk8ip2/Grin2b/Shank1	3
BP	GO:0021680	cerebellar Purkinje cell layer developme	5/373	33/19943	0.000344	0.003679	0.0028642	Sez6l2/Atp2b2/Klhl1/Cend1/Sptbn2	5
BP	GO:0010769	regulation of cell morphogenesis involve	17/373	350/19943	0.000345	0.003687	0.00287066	Arhgap44/Thy1/Grin1/Dmtn/Nefl/Nefm/Hecw1/Camk2b/Slit1/Cit,	17
BP	GO:0002090	regulation of receptor internalization	7/373	71/19943	0.00036	0.003836	0.00298616	Nsf/Napb/Hpca/Drd2/Pacsin1/Necab2/Nrg1	7
BP	GO:0060291	long-term synaptic potentiation	12/373	198/19943	0.000368	0.003911	0.00304488	Slc24a2/Drd2/Rims1/Calb2/Syt12/Camk2b/Adcy1/Grin2b/Chrna7/	12
BP	GO:1904064	positive regulation of cation transmemb	11/373	170/19943	0.000369	0.003911	0.00304488	Cacng2/Thy1/Cacng3/Ryr2/Rgs7/Dpp6/Kcnc1/Kcna1/Akap6/Cacn	11
BP	GO:0051350	negative regulation of lyase activity	4/373	19/19943	0.000374	0.003933	0.00306165	Hpca/Drd2/Hrh3/Akap6	4
BP	GO:1902259	regulation of delayed rectifier potassium	4/373	19/19943	0.000374	0.003933	0.00306165	Kcns2/Kcnab1/Akap6/Kcng1	4
BP	GO:0007632	visual behavior	7/373	72/19943	0.000392	0.004121	0.00320836	Tafa2/Drd2/Grin1/Ndrg4/Atp1a3/Neto1/Chrnb2	7
BP	GO:0017085	response to insecticide	5/373	34/19943	0.000397	0.004156	0.00323544	Kif5a/Nefh/Nefl/Ddc/Map1b	5
BP	GO:0090174	organelle membrane fusion	8/373	96/19943	0.00044	0.004588	0.00357166	Cplx1/Nsf/Syt2/Rims1/Stx1b/Vamp1/Rab3a/Syt7	8
BP	GO:0048284	organelle fusion	9/373	121/19943	0.000456	0.004733	0.00368483	Cplx1/Nsf/Syt2/Gdap1/Rims1/Stx1b/Vamp1/Rab3a/Syt7	9
BP	GO:0001975	response to amphetamine	6/373	54/19943	0.000498	0.005141	0.00400232	Drd2/Grin1/Rasgrf1/Rgs7/Grin2b/Rgs17	6
BP	GO:0099641	anterograde axonal protein transport	3/373	9/19943	0.000501	0.005141	0.00400232	Kif5a/Kif5c/Dlg2	3
BP	GO:1905383	protein localization to presynapse	3/373	9/19943	0.000501	0.005141	0.00400232	Kif5a/Kif5c/Dlg2	3
BP	GO:0032228	regulation of synaptic transmission, GAB	5/373	36/19943	0.000521	0.005296	0.00412253	Phf24/Drd2/Cntnap4/Car7/Prkce	5
BP	GO:0043950	positive regulation of cAMP-mediated si	5/373	36/19943	0.000521	0.005296	0.00412253	Gpr3/Gpr61/Chga/Mrap2/Gnas	5
BP	GO:0099563	modification of synaptic structure	5/373	36/19943	0.000521	0.005296	0.00412253	Cabp1/Arhgap44/Dlgap3/Pfn2/LOC100909840	5
BP	GO:0007218	neuropeptide signaling pathway	8/373	99/19943	0.000541	0.005486	0.00427093	Glra2/Scg5/Oprd1/Hcrtr2/Glra1/Hcrtr1/Tac3/Oprl1	8
BP	GO:0008090	retrograde axonal transport	4/373	21/19943	0.00056	0.005611	0.0043683	Kif5a/Nefl/Dlg2/Bsn	4
BP	GO:0030100	regulation of endocytosis	15/373	300/19943	0.000564	0.005637	0.00438844	Nsf/Napb/Hpca/Drd2/Rit2/Stx1b/Pacsin1/Necab2/Sgip1/Nrg1/Mc	15
BP	GO:0014075	response to amine	7/373	77/19943	0.000592	0.0059	0.00459309	Drd2/Grin1/Rasgrf1/Rgs7/Kcnc1/Grin2b/Rgs17	7
BP	GO:0008088	axo-dendritic transport	7/373	78/19943	0.00064	0.006365	0.00495492	Kif5a/Nefl/Nefm/Kif5c/Map2/Dlg2/Bsn	7
BP	GO:0006584	catecholamine metabolic process	6/373	57/19943	0.000668	0.006597	0.00513601	Drd2/Sncb/Htr2c/Ddc/Chrna7/Chrnb2	6
BP	GO:0009712	catechol-containing compound metaboli	6/373	57/19943	0.000668	0.006597	0.00513601	Drd2/Sncb/Htr2c/Ddc/Chrna7/Chrnb2	6
BP	GO:0099172	presynapse organization	6/373	57/19943	0.000668	0.006597	0.00513601	Lrfn5/Slitrk3/Clstn3/Cbln1/Cntn5/Bsn	6
BP	GO:0007156	homophilic cell adhesion via plasma mer	9/373	128/19943	0.000687	0.006757	0.00526022	Cntn4/Clstn3/Cdh12/Cdh7/Ptprt/Clstn2/Pcdha4/Celsr3/L1cam	9
BP	GO:0006865	amino acid transport	10/373	155/19943	0.000691	0.006783	0.00528044	Grm4/Grm1/Slc32a1/Slc17a6/Htr2c/Slc6a5/Slc6a11/Slc7a10/Hrh3	10
BP	GO:0071420	cellular response to histamine	3/373	10/19943	0.000706	0.00684	0.00532521	Gabrg2/Gabra1/Gabrb3	3
BP	GO:0098700	neurotransmitter loading into synaptic v	3/373	10/19943	0.000706	0.00684	0.00532521	Slc32a1/Slc17a6/Ddc	3
BP	GO:0098953	receptor diffusion trapping	3/373	10/19943	0.000706	0.00684	0.00532521	Cacng2/Cacng3/Camk2a	3
BP	GO:0098970	postsynaptic neurotransmitter receptor	3/373	10/19943	0.000706	0.00684	0.00532521	Cacng2/Cacng3/Camk2a	3
BP	GO:0099628	neurotransmitter receptor diffusion trap	3/373	10/19943	0.000706	0.00684	0.00532521	Cacng2/Cacng3/Camk2a	3
BP	GO:0043279	response to alkaloid	11/373	184/19943	0.000717	0.006931	0.00539577	Drd2/Nefh/Grin1/Ryr2/Crhbp/Tac3/Ddc/Grin2b/Htr2a/Prkce/Chrr	11
BP	GO:0007528	neuromuscular junction development	6/373	58/19943	0.000733	0.007068	0.00550245	Cacng2/Ky/Cacna2d2/Shank1/Chat/Unc13c	6
BP	GO:0072384	organelle transport along microtubule	7/373	80/19943	0.000745	0.007168	0.00558028	Kif5a/Nefh/Dync1i1/Nefl/Rasgrp1/Map2/Map1b	7
BP	GO:0060047	heart contraction	12/373	215/19943	0.000767	0.007326	0.00570293	Drd2/Ryr2/Atp1a3/Scn4b/Chrm2/Chga/Scn1a/Cacna1b/Akap6/Ch	12
BP	GO:0034767	positive regulation of ion transmembran	11/373	186/19943	0.000784	0.007479	0.00582221	Cacng2/Thy1/Cacng3/Ryr2/Rgs7/Dpp6/Kcnc1/Kcna1/Akap6/Cacn	11
BP	GO:0048639	positive regulation of developmental grc	12/373	216/19943	0.000798	0.007596	0.00591321	Syt2/Drd2/Rims1/Atp8a2/Rims2/Akap6/Sgip1/Nrg1/Cacna2d2/M	12
BP	GO:0098930	axonal transport	6/373	59/19943	0.000803	0.007628	0.00593836	Kif5a/Nefl/Kif5c/Map2/Dlg2/Bsn	6
BP	GO:0098664	G protein-coupled serotonin receptor sig	5/373	40/19943	0.000856	0.008045	0.00626309	Htr2c/Chrm2/Hrh3/Htr2a/Htr4	5
BP	GO:1901385	regulation of voltage-gated calcium char	5/373	40/19943	0.000856	0.008045	0.00626309	Cabp1/Hpca/Drd2/Oprl1/Cacna2d1	5
BP	GO:1990573	potassium ion import across plasma mer	5/373	40/19943	0.000856	0.008045	0.00626309	Kcnj3/Slc12a5/Atp1a3/Kcnj12/Slc12a3	5
BP	GO:0051209	release of sequestered calcium ion into	9/373	132/19943	0.000857	0.008045	0.00626309	Thy1/Drd2/Ryr2/Htr2c/Plch2/Jph3/Akap6/Htr2a/Prkce	9
BP	GO:2001259	positive regulation of cation channel acti	7/373	82/19943	0.000864	0.008096	0.00630226	Cacng2/Cacng3/Kcnc1/Kcna1/Akap6/Cacna2d1/Hcn1	7
BP	GO:0015698	inorganic anion transport	10/373	161/19943	0.000926	0.008661	0.00674237	Gabrg2/Glra2/Gabra1/Gabra5/Car7/Glra1/Slc12a5/Gabra3/Slc12a	10
BP	GO:0098810	neurotransmitter reuptake	4/373	24/19943	0.000951	0.008771	0.00682815	Drd2/Slc6a5/Nat8l/Slc6a11	4
BP	GO:0051283	negative regulation of sequestering of ca	9/373	134/19943	0.000953	0.008771	0.00682815	Thy1/Drd2/Ryr2/Htr2c/Plch2/Jph3/Akap6/Htr2a/Prkce	9
BP	GO:0033603	positive regulation of dopamine secretio	3/373	11/19943	0.000958	0.008771	0.00682815	Chrna4/Pcp4/Chrnb2	3
BP	GO:0038003	opioid receptor signaling pathway	3/373	11/19943	0.000958	0.008771	0.00682815	Oprd1/Syp/Oprl1	3
BP	GO:0048149	behavioral response to ethanol	3/373	11/19943	0.000958	0.008771	0.00682815	Drd2/Crhbp/Chrna7	3
BP	GO:1904862	inhibitory synapse assembly	3/373	11/19943	0.000958	0.008771	0.00682815	Gabrg2/Gabra1/Gabrb3	3
BP	GO:1990504	dense core granule exocytosis	3/373	11/19943	0.000958	0.008771	0.00682815	Cadps2/Cadps/Unc13c	3
BP	GO:0070050	neuron cellular homeostasis	5/373	41/19943	0.00096	0.008771	0.00682815	Ncs1/Calb2/Atp2b3/Kcnh1/Cacna1b	5
BP	GO:0098900	regulation of action potential	6/373	62/19943	0.001046	0.009536	0.00742368	Rapgef4/Fgf12/Ryr2/Chrnb2/Cacna2d1/Hcn1	6
BP	GO:0051282	regulation of sequestering of calcium ior	9/373	136/19943	0.001059	0.009594	0.00746894	Thy1/Drd2/Ryr2/Htr2c/Plch2/Jph3/Akap6/Htr2a/Prkce	9
BP	GO:0008015	blood circulation	20/373	493/19943	0.001062	0.009598	0.00747193	Drd2/Ryr2/Atp1a3/Scn4b/Htr2c/Chrm2/Chga/Scn1a/Tac3/Grip2/(	20
BP	GO:1903169	regulation of calcium ion transmembran	10/373	164/19943	0.001066	0.00962	0.00748878	Cabp1/Thy1/Hpca/Drd2/Ryr2/Jph3/Akap6/Prkce/Oprl1/Cacna2d1	10
BP	GO:0007210	serotonin receptor signaling pathway	5/373	42/19943	0.001073	0.009664	0.00752311	Htr2c/Chrm2/Hrh3/Htr2a/Htr4	5
BP	GO:0003015	heart process	12/373	224/19943	0.001094	0.009831	0.00765316	Drd2/Ryr2/Atp1a3/Scn4b/Chrm2/Chga/Scn1a/Cacna1b/Akap6/Ch	12
BP	GO:0051208	sequestering of calcium ion	9/373	137/19943	0.001115	0.009966	0.00775868	Thy1/Drd2/Ryr2/Htr2c/Plch2/Jph3/Akap6/Htr2a/Prkce	9
BP	GO:0035864	response to potassium ion	4/373	25/19943	0.001116	0.009966	0.00775868	Nptx1/Crhbp/Kcnc1/Dlg2	4
BP	GO:1902473	regulation of protein localization to syna	4/373	25/19943	0.001116	0.009966	0.00775868	Rapgef4/Arhgap44/Clstn3/Camk2a	4

BP	GO:0021695	cerebellar cortex development	6/373	63/19943	0.001138	0.010143	0.00789627	Sez6l2/Atp2b2/Cbln1/Klhl1/Cend1/Sptbn2	6
BP	GO:0018958	phenol-containing compound metabolic	8/373	111/19943	0.001151	0.010235	0.00796775	Tph2/Drd2/Sncb/Atp2b2/Htr2c/Ddc/Chrna7/Chrnb2	8
BP	GO:0009914	hormone transport	17/373	390/19943	0.001154	0.010244	0.00797494	Cplx1/Rapgef4/Vsnl1/Scg5/Drd2/Pex5l/Gpld1/Crhbp/Htr2c/Chga/	17
BP	GO:1904375	regulation of protein localization to cell	9/373	139/19943	0.001235	0.010937	0.00851444	Rapgef4/Arhgap44/Cacng2/Grm1/Dpp6/Camk2b/Mrap2/Camk2a/	9
BP	GO:0098887	neurotransmitter receptor transport, en	3/373	12/19943	0.001259	0.011088	0.0086317	Arhgap44/Grip2/Lrrc7	3
BP	GO:0006898	receptor-mediated endocytosis	12/373	228/19943	0.001273	0.011186	0.00870791	Nsf/Napb/Cacng2/Hpca/Drd2/Cacng3/Pacsin1/Necab2/Sgip1/Nrg	12
BP	GO:0098656	anion transmembrane transport	13/373	260/19943	0.001293	0.01126	0.00876588	Gabrg2/Glra2/Gabra1/Gabra5/Glra1/Slc32a1/Slc17a6/Slc12a5/Slc	13
BP	GO:0021510	spinal cord development	9/373	140/19943	0.001298	0.01126	0.00876588	Rapgef4/Glra2/Nefh/Nefl/Nefm/Acan/Slit1/Lhx4/Nova1	9
BP	GO:0060004	reflex	4/373	26/19943	0.0013	0.01126	0.00876588	Cacng2/Glra1/Kcnma1/Shank1	4
BP	GO:2000311	regulation of AMPA receptor activity	4/373	26/19943	0.0013	0.01126	0.00876588	Cacng2/Cacng3/Mapk8ip2/Shank1	4
BP	GO:0021675	nerve development	7/373	89/19943	0.001401	0.012065	0.00939238	Nptx1/Gabra5/Kcna2/Pax2/Chrnb2/Gabrb3/Pou4f1	7
BP	GO:0010107	potassium ion import	5/373	45/19943	0.001473	0.012583	0.00979572	Kcnj3/Slc12a5/Atp1a3/Kcnj12/Slc12a3	5
BP	GO:1901381	positive regulation of potassium ion tran	5/373	45/19943	0.001473	0.012583	0.00979572	Rgs7/Dpp6/Kcnc1/Kcna1/Akap6	5
BP	GO:2001258	negative regulation of cation channel act	5/373	45/19943	0.001473	0.012583	0.00979572	Cabp1/Fgf12/Drd2/Kcnab1/Oprl1	5
BP	GO:1904063	negative regulation of cation transmemb	7/373	90/19943	0.001495	0.012751	0.00992617	Cabp1/Fgf12/Drd2/Hecw1/Kcnab1/Prkce/Oprl1	7
BP	GO:0097305	response to alcohol	18/373	437/19943	0.001575	0.013377	0.01041345	Glra2/Kif5a/Drd2/Grin1/Glra1/Nefl/Gpld1/Crhbp/Rgs7/Gnas/Cacn	18
BP	GO:0002028	regulation of sodium ion transport	7/373	91/19943	0.001594	0.013517	0.01052241	Fgf12/Drd2/Hecw1/Scn4b/Gnas/Neto1/Neto2	7
BP	GO:0048814	regulation of dendrite morphogenesis	8/373	117/19943	0.001614	0.013584	0.01057497	Arhgap44/Grin1/Hecw1/Camk2b/Cit/Chrnb2/Dnm3/Trpc5	8
BP	GO:0099639	neurotransmitter receptor transport, en	3/373	13/19943	0.001615	0.013584	0.01057497	Arhgap44/Grip2/Lrrc7	3
BP	GO:1902950	regulation of dendritic spine maintenanc	3/373	13/19943	0.001615	0.013584	0.01057497	Grin1/Zfp804a/Grin2b	3
BP	GO:0043954	cellular component maintenance	6/373	68/19943	0.001696	0.01424	0.01108561	Grin1/Rab3a/Zfp804a/Grin2b/Dlg2/Bsn	6
BP	GO:0035235	ionotropic glutamate receptor signaling	4/373	28/19943	0.001728	0.014374	0.0111902	Grin1/Atp1a3/Camk2a/Grin2b	4
BP	GO:0045761	regulation of adenylate cyclase activity	4/373	28/19943	0.001728	0.014374	0.0111902	Hpca/Drd2/Hrh3/Akap6	4
BP	GO:0060384	innervation	4/373	28/19943	0.001728	0.014374	0.0111902	Nptx1/Gabra5/Gabrb3/Pou4f1	4
BP	GO:0099010	modification of postsynaptic structure	4/373	28/19943	0.001728	0.014374	0.0111902	Cabp1/Arhgap44/Pfn2/LOC100909840	4
BP	GO:0008089	anterograde axonal transport	5/373	47/19943	0.001793	0.014858	0.01156693	Kif5a/Nefl/Kif5c/Map2/Dlg2	5
BP	GO:0030431	sleep	5/373	47/19943	0.001793	0.014858	0.01156693	Drd2/Kcna2/Htr2a/Chrnb2/Cacna1i	5
BP	GO:0032414	positive regulation of ion transmembran	8/373	119/19943	0.001798	0.014865	0.01157197	Cacng2/Cacng3/Ryr2/Kcnc1/Kcna1/Akap6/Cacna2d1/Hcn1	8
BP	GO:0051235	maintenance of location	15/373	339/19943	0.001914	0.015796	0.01229718	Vstm2a/Thy1/Drd2/Pex5l/Epb41l3/Ryr2/Rit2/Htr2c/Mest/Plch2/J	15
BP	GO:0048488	synaptic vesicle endocytosis	6/373	70/19943	0.001969	0.016154	0.01257528	Syt2/Sncb/Pacsin1/Syp/Syt7/Dnm3	6
BP	GO:0140238	presynaptic endocytosis	6/373	70/19943	0.001969	0.016154	0.01257528	Syt2/Sncb/Pacsin1/Syp/Syt7/Dnm3	6
BP	GO:0045104	intermediate filament cytoskeleton orga	5/373	48/19943	0.001972	0.016154	0.01257528	Nefh/Nefl/Nefm/Ina/Atp8a2	5
BP	GO:1900273	positive regulation of long-term synaptic	4/373	29/19943	0.001976	0.016154	0.01257528	Drd2/Adcy1/Grin2b/Chrna7	4
BP	GO:2000310	regulation of NMDA receptor activity	4/373	29/19943	0.001976	0.016154	0.01257528	Rph3a/Rasgrf1/Crhbp/Mapk8ip2	4
BP	GO:0021554	optic nerve development	3/373	14/19943	0.002027	0.016326	0.01270915	Kcna2/Pax2/Chrnb2	3
BP	GO:0030007	cellular potassium ion homeostasis	3/373	14/19943	0.002027	0.016326	0.01270915	Drd2/Atp1a3/Kcnma1	3
BP	GO:0032252	secretory granule localization	3/373	14/19943	0.002027	0.016326	0.01270915	Kif5a/Rasgrp1/Map2	3
BP	GO:0042428	serotonin metabolic process	3/373	14/19943	0.002027	0.016326	0.01270915	Tph2/Atp2b2/Ddc	3
BP	GO:0046879	hormone secretion	16/373	376/19943	0.002036	0.016373	0.01274576	Cplx1/Rapgef4/Vsnl1/Scg5/Drd2/Pex5l/Gpld1/Crhbp/Htr2c/Chga/	16
BP	GO:0021766	hippocampus development	8/373	123/19943	0.002214	0.017703	0.01378132	Kif5a/Nefh/Nefl/Nefm/Slc32a1/Slc17a6/Kcna1/Grin2b	8
BP	GO:0050795	regulation of behavior	7/373	97/19943	0.002301	0.018331	0.0142702	Drd2/Cntnap4/Hcrtr2/Kcna2/Sgip1/Oprl1/Chrnb2	7
BP	GO:0042755	eating behavior	5/373	50/19943	0.002367	0.018752	0.01459826	Oprd1/Atp8a2/Hrh3/Sgip1/Oprl1	5
BP	GO:0003012	muscle system process	17/373	418/19943	0.0024	0.018983	0.01477758	Drd2/Glra1/Ryr2/Atp1a3/Scn4b/Chrm2/Chga/Scn1a/Atp8a2/Grip	17
BP	GO:0048511	rhythmic process	16/373	383/19943	0.002447	0.019321	0.01504091	Tph2/Drd2/Hcrtr2/Grin1/Kcna2/Chga/Ddc/Adcy1/Mapk10/Kcnma	16
BP	GO:0021700	developmental maturation	14/373	314/19943	0.002498	0.019478	0.01516308	Gpr3/Grin1/Nefl/Rab3a/Sez6l2/Camk2b/Grip2/Pax2/Kcnma1/Ma	14
BP	GO:0007194	negative regulation of adenylate cyclase	3/373	15/19943	0.002498	0.019478	0.01516308	Drd2/Hrh3/Akap6	3
BP	GO:0031340	positive regulation of vesicle fusion	3/373	15/19943	0.002498	0.019478	0.01516308	Syt2/Rims1/Syt7	3
BP	GO:1901160	primary amino compound metabolic pro	3/373	15/19943	0.002498	0.019478	0.01516308	Tph2/Atp2b2/Ddc	3
BP	GO:1901386	negative regulation of voltage-gated cal	3/373	15/19943	0.002498	0.019478	0.01516308	Cabp1/Drd2/Oprl1	3
BP	GO:1903818	positive regulation of voltage-gated pot	3/373	15/19943	0.002498	0.019478	0.01516308	Kcnc1/Kcna1/Akap6	3
BP	GO:0060074	synapse maturation	4/373	31/19943	0.002542	0.019744	0.01537005	Nefl/Sez6l2/Camk2b/Shank1	4
BP	GO:0009582	detection of abiotic stimulus	8/373	126/19943	0.002573	0.019917	0.01550516	Phf24/Ano3/Atp2b2/Scn1a/Atp8a2/Kcna1/Grin2b/Htr2a	8
BP	GO:0032411	positive regulation of transporter activit	8/373	126/19943	0.002573	0.019917	0.01550516	Cacng2/Cacng3/Ryr2/Kcnc1/Kcna1/Akap6/Cacna2d1/Hcn1	8
BP	GO:2000649	regulation of sodium ion transmembran	5/373	51/19943	0.002584	0.019969	0.01554528	Fgf12/Hecw1/Scn4b/Neto1/Neto2	5
BP	GO:0008016	regulation of heart contraction	10/373	185/19943	0.002607	0.020094	0.01564269	Drd2/Ryr2/Scn4b/Chrm2/Chga/Cacna1b/Akap6/Chrna7/Cacna2d	10
BP	GO:0021761	limbic system development	9/373	155/19943	0.002612	0.020094	0.01564269	Kif5a/Drd2/Nefh/Nefl/Nefm/Slc32a1/Slc17a6/Kcna1/Grin2b	9
BP	GO:0032413	negative regulation of ion transmembra	6/373	74/19943	0.002614	0.020094	0.01564269	Cabp1/Fgf12/Drd2/Hecw1/Kcnab1/Oprl1	6
BP	GO:0009581	detection of external stimulus	8/373	127/19943	0.002702	0.020698	0.01611287	Phf24/Ano3/Atp2b2/Scn1a/Atp8a2/Kcna1/Grin2b/Htr2a	8
BP	GO:0007193	adenylate cyclase-inhibiting G protein-c	6/373	75/19943	0.002798	0.021391	0.01665252	Grm4/Drd2/Oprd1/Chrm2/Hrh3/Oprl1	6
BP	GO:2001222	regulation of neuron migration	5/373	52/19943	0.002816	0.021493	0.01673216	Scrt1/Camk2b/Camk2a/Nrg1/Nexmif	5
BP	GO:1903522	regulation of blood circulation	12/373	251/19943	0.002835	0.021575	0.01679596	Drd2/Ryr2/Scn4b/Htr2c/Chrm2/Chga/Cacna1b/Akap6/Htr2a/Chrn	12
BP	GO:0098884	postsynaptic neurotransmitter receptor	4/373	32/19943	0.002862	0.021619	0.01683001	Hpca/Pacsin1/Nrg1/Dnm3	4
BP	GO:0140239	postsynaptic endocytosis	4/373	32/19943	0.002862	0.021619	0.01683001	Hpca/Pacsin1/Nrg1/Dnm3	4
BP	GO:1902074	response to salt	4/373	32/19943	0.002862	0.021619	0.01683001	Hpca/Nefh/Nefl/Pcp4	4
BP	GO:0098876	vesicle-mediated transport to the plasm	7/373	101/19943	0.002892	0.021803	0.01697295	Cplx1/Arhgap44/Nsf/Stx1b/Grip2/Mapk10/Lrrc7	7
BP	GO:0051965	positive regulation of synapse assembly	6/373	76/19943	0.002991	0.022438	0.01746794	Slitrk3/Clstn3/Syndig1/Cbln1/Lingo4/Clstn2	6
BP	GO:0001967	suckling behavior	3/373	16/19943	0.003033	0.022438	0.01746794	Grin1/Grin2b/Pou4f1	3
BP	GO:0035865	cellular response to potassium ion	3/373	16/19943	0.003033	0.022438	0.01746794	Nptx1/Crhbp/Dlg2	3
BP	GO:0098926	postsynaptic signal transduction	3/373	16/19943	0.003033	0.022438	0.01746794	Cabp1/Nrg1/Camk4	3
BP	GO:0099527	postsynapse to nucleus signaling pathw	3/373	16/19943	0.003033	0.022438	0.01746794	Cabp1/Nrg1/Camk4	3
BP	GO:0099640	axo-dendritic protein transport	3/373	16/19943	0.003033	0.022438	0.01746794	Kif5a/Kif5c/Dlg2	3
BP	GO:1900271	regulation of long-term synaptic potenti	5/373	53/19943	0.003063	0.022584	0.017581	Drd2/Calb2/Adcy1/Grin2b/Chrna7	5
BP	GO:1904645	response to amyloid-beta	5/373	53/19943	0.003063	0.022584	0.017581	Atp1a3/Syp/Cacna1b/Chrna7/Cacna2d1	5
BP	GO:0046717	acid secretion	8/373	130/19943	0.003121	0.022976	0.01788637	Grm4/Drd2/Grm1/Htr2c/Hrh3/Grin2b/Oprl1/Abcc2	8
BP	GO:0030072	peptide hormone secretion	13/373	288/19943	0.003165	0.023257	0.01810484	Cplx1/Rapgef4/Vsnl1/Drd2/Pex5l/Gpld1/Crhbp/Htr2c/Chga/Stxbp	13
BP	GO:0022037	metencephalon development	9/373	160/19943	0.00323	0.023656	0.01841552	Grin1/Ckmt1/Sez6l2/Atp2b2/Kcnc1/Cbln1/Klhl1/Cend1/Sptbn2	9
BP	GO:0045103	intermediate filament-based process	5/373	54/19943	0.003324	0.02413	0.01878472	Nefh/Nefl/Nefm/Ina/Atp8a2	5
BP	GO:0010513	positive regulation of phosphatidylinosit	2/373	5/19943	0.003361	0.02413	0.01878472	Htr2c/Htr2a	2
BP	GO:0099502	calcium-dependent activation of synapti	2/373	5/19943	0.003361	0.02413	0.01878472	Syt2/Syt7	2
BP	GO:1902952	positive regulation of dendritic spine ma	2/373	5/19943	0.003361	0.02413	0.01878472	Grin1/Zfp804a	2
BP	GO:0007009	plasma membrane organization	7/373	104/19943	0.003406	0.024336	0.01894504	Ano3/Epb41l3/Rab3a/Pacsin1/Sgip1/Xkr6/Syt7	7
BP	GO:0010771	negative regulation of cell morphogenes	7/373	104/19943	0.003406	0.024336	0.01894504	Thy1/Dmtn/Slit1/Cit/Map2/Dnm3/Trpc5	7
BP	GO:0003407	neural retina development	6/373	78/19943	0.003406	0.024336	0.01894504	Thy1/Slc17a6/Atp2b2/Atp2b3/Atp8a2/Hcn1	6
BP	GO:0009612	response to mechanical stimulus	14/373	326/19943	0.003497	0.024942	0.01941662	Phf24/Ano3/Drd2/Ryr2/Acan/Atp2b2/Scn1a/Kcnc1/Atp8a2/Pax2/	14
BP	GO:0042749	regulation of circadian sleep/wake cycle	4/373	34/19943	0.003585	0.025388	0.0197643	Drd2/Hcrtr2/Kcna2/Chrnb2	4
BP	GO:1905606	regulation of presynapse assembly	4/373	34/19943	0.003585	0.025388	0.0197643	Lrfn5/Slitrk3/Clstn3/Cbln1	4
BP	GO:0043268	positive regulation of potassium ion tran	5/373	55/19943	0.003602	0.025399	0.01977238	Rgs7/Dpp6/Kcnc1/Kcna1/Akap6	5
BP	GO:0055064	chloride ion homeostasis	3/373	17/19943	0.003632	0.025487	0.01984144	Slc12a5/Abcc2/Slc12a3	3
BP	GO:0099638	endosome to plasma membrane protein	3/373	17/19943	0.003632	0.025487	0.01984144	Arhgap44/Grip2/Lrrc7	3
BP	GO:0060041	retina development in camera-type eye	10/373	194/19943	0.003663	0.025663	0.01997851	Thy1/Hpca/Slc17a6/Atp2b2/Atp2b3/Atp8a2/Pax2/Celf4/Tub/Hcn	10
BP	GO:1990138	neuron projection extension	10/373	195/19943	0.003798	0.026571	0.02068513	Syt2/Rims1/Nrn1/Slit1/Rims2/Nrg1/Map2/Map1b/Trpc5/L1cam	10
BP	GO:0098659	inorganic cation import across plasma m	6/373	80/19943	0.003863	0.026939	0.02097142	Kcnj3/Slc12a5/Atp1a3/Kcnj12/Slc8a3/Slc12a3	6
BP	GO:0099587	inorganic ion import across plasma mem	6/373	80/19943	0.003863	0.026939	0.02097142	Kcnj3/Slc12a5/Atp1a3/Kcnj12/Slc8a3/Slc12a3	6
BP	GO:0061025	membrane fusion	8/373	135/19943	0.003929	0.027354	0.02129446	Cplx1/Nsf/Syt2/Rims1/Stx1b/Vamp1/Rab3a/Syt7	8
BP	GO:0034766	negative regulation of ion transmembra	7/373	107/19943	0.003988	0.027679	0.02154746	Cabp1/Fgf12/Drd2/Hecw1/Kcnab1/Prkce/Oprl1	7
BP	GO:0045745	positive regulation of G protein-coupled	4/373	35/19943	0.003989	0.027679	0.02154746	Drd2/Chga/Mrap2/Necab2	4
BP	GO:0031102	neuron projection regeneration	6/373	81/19943	0.004108	0.028457	0.02215356	Thy1/Nefh/Nefl/Nefm/Nrg1/Map1b	6
BP	GO:0009410	response to xenobiotic stimulus	16/373	404/19943	0.004114	0.028457	0.02215356	Hpca/Drd2/Nefh/Grin1/Rasgrf1/Ryr2/Crhbp/Rgs7/Tac3/Grin2b/H	16
BP	GO:0090150	establishment of protein localization to	11/373	230/19943	0.004171	0.028806	0.02242501	Cplx1/Arhgap44/Nsf/Cacng2/Hpca/Dmtn/Stx1b/Grip2/Mapk10/S	11
BP	GO:0014051	gamma-aminobutyric acid secretion	3/373	18/19943	0.004298	0.0295	0.02296492	Grm4/Htr2c/Hrh3	3
BP	GO:0021681	cerebellar granular layer development	3/373	18/19943	0.004298	0.0295	0.02296492	Atp2b2/Cbln1/Cend1	3

BP	GO:0097091	synaptic vesicle clustering	3/373	18/19943	0.004298	0.0295	0.02296492	Syn1/Rab3a/Syndig1	3
BP	GO:0098885	modification of postsynaptic actin cytosk	3/373	18/19943	0.004298	0.0295	0.02296492	Cabp1/Pfn2/LOC100909840	3
BP	GO:0021537	telencephalon development	14/373	334/19943	0.004328	0.029613	0.02305336	Kif5a/Drd2/Nefh/Grin1/Nefl/Nefm/Slc32a1/Slc17a6/Atp1a3/Kcna	14
BP	GO:0042220	response to cocaine	6/373	82/19943	0.004364	0.029812	0.02320822	Drd2/Nefh/Crhbp/Grin2b/Htr2a/Chrnb2	6
BP	GO:2000171	negative regulation of dendrite developr	4/373	36/19943	0.004422	0.030158	0.02347764	RGD1307443/Cit/Dnm3/Trpc5	4
BP	GO:0006941	striated muscle contraction	9/373	169/19943	0.004628	0.031493	0.02451664	Ryr2/Atp1a3/Scn4b/Chga/Scn1a/Atp8a2/Akap6/Slc8a3/Cacna2d1	9
BP	GO:0032410	negative regulation of transporter activit	6/373	83/19943	0.004632	0.031493	0.02451664	Cabp1/Fgf12/Drd2/Hecw1/Kcnab1/Oprl1	6
BP	GO:0007018	microtubule-based movement	12/373	267/19943	0.004647	0.031545	0.02455758	Kif5a/Nefh/Dync1i1/Nefl/Nefm/Kif5c/Rasgrp1/Map2/Map1b/Dlg2	12
BP	GO:0098739	import across plasma membrane	7/373	111/19943	0.004876	0.032778	0.0255171	Kcnj3/Slc12a5/Atp1a3/Slc6a5/Kcnj12/Slc8a3/Slc12a3	7
BP	GO:0045744	negative regulation of G protein-couplec	5/373	59/19943	0.004879	0.032778	0.0255171	Drd2/Camk2b/Mrap2/Necab2/Oprl1	5
BP	GO:0051482	positive regulation of cytosolic calcium ic	4/373	37/19943	0.004886	0.032778	0.0255171	Drd2/Grm1/Htr2c/Oprl1	4
BP	GO:0097366	response to bronchodilator	6/373	84/19943	0.004912	0.032778	0.0255171	Drd2/Grin1/Rasgrf1/Rgs7/Grin2b/Rgs17	6
BP	GO:0007016	cytoskeletal anchoring at plasma membr	2/373	6/19943	0.004979	0.032778	0.0255171	Epb41l3/Shank1	2
BP	GO:0008291	acetylcholine metabolic process	2/373	6/19943	0.004979	0.032778	0.0255171	Slc5a7/Chat	2
BP	GO:0031133	regulation of axon diameter	2/373	6/19943	0.004979	0.032778	0.0255171	Nefl/Nefm	2
BP	GO:0042435	indole-containing compound biosyntheti	2/373	6/19943	0.004979	0.032778	0.0255171	Tph2/Ddc	2
BP	GO:0051584	regulation of dopamine uptake involved	2/373	6/19943	0.004979	0.032778	0.0255171	Drd2/Nat8l	2
BP	GO:0051940	regulation of catecholamine uptake invo	2/373	6/19943	0.004979	0.032778	0.0255171	Drd2/Nat8l	2
BP	GO:0098937	anterograde dendritic transport	2/373	6/19943	0.004979	0.032778	0.0255171	Kif5a/Kif5c	2
BP	GO:1900619	acetate ester metabolic process	2/373	6/19943	0.004979	0.032778	0.0255171	Slc5a7/Chat	2
BP	GO:1904008	response to monosodium glutamate	2/373	6/19943	0.004979	0.032778	0.0255171	Hpca/Pcp4	2
BP	GO:0021692	cerebellar Purkinje cell layer morphogen	3/373	19/19943	0.005034	0.032843	0.02556785	Atp2b2/Cend1/Sptbn2	3
BP	GO:0070886	positive regulation of calcineurin-NFAT s	3/373	19/19943	0.005034	0.032843	0.02556785	Akap6/Nrg1/Camta1	3
BP	GO:0098840	protein transport along microtubule	3/373	19/19943	0.005034	0.032843	0.02556785	Kif5a/Kif5c/Dlg2	3
BP	GO:0099118	microtubule-based protein transport	3/373	19/19943	0.005034	0.032843	0.02556785	Kif5a/Kif5c/Dlg2	3
BP	GO:0106058	positive regulation of calcineurin-mediat	3/373	19/19943	0.005034	0.032843	0.02556785	Akap6/Nrg1/Camta1	3
BP	GO:0043624	cellular protein complex disassembly	8/373	141/19943	0.005102	0.033236	0.02587392	Nsf/Napb/Nrg1/Map2/Add2/Map1b/Ogfod1/Sptbn2	8
BP	GO:0097306	cellular response to alcohol	9/373	172/19943	0.005185	0.033729	0.02625728	Glra2/Kif5a/Glra1/Gpld1/Gnas/Grin2b/Prkce/Sptbn2/L1cam	9
BP	GO:0043112	receptor metabolic process	10/373	204/19943	0.005207	0.033816	0.02632552	Nsf/Napb/Cacng2/Hpca/Drd2/Cacng3/Pacsin1/Necab2/Nrg1/Dnm	10
BP	GO:0035176	social behavior	5/373	60/19943	0.005242	0.033846	0.02634842	Grin1/Mapk8ip2/Shank1/Chrnb2/Nrxn3	5
BP	GO:0051703	intraspecies interaction between organi:	5/373	60/19943	0.005242	0.033846	0.02634842	Grin1/Mapk8ip2/Shank1/Chrnb2/Nrxn3	5
BP	GO:0090102	cochlea development	5/373	60/19943	0.005242	0.033846	0.02634842	Pvalb/Gabra5/Atp2b2/Pax2/Gabrb3	5
BP	GO:1902305	regulation of sodium ion transmembran	5/373	60/19943	0.005242	0.033846	0.02634842	Fgf12/Hecw1/Scn4b/Neto1/Neto2	5
BP	GO:0022410	circadian sleep/wake cycle process	4/373	38/19943	0.005382	0.034592	0.02692929	Drd2/Hcrtr2/Kcna2/Chrnb2	4
BP	GO:0099174	regulation of presynapse organization	4/373	38/19943	0.005382	0.034592	0.02692929	Lrfn5/Slitrk3/Clstn3/Cbln1	4
BP	GO:1903076	regulation of protein localization to plasr	7/373	114/19943	0.005635	0.036168	0.0281564	Arhgap44/Grm1/Dpp6/Camk2b/Mrap2/Camk2a/Dpp10	7
BP	GO:0050770	regulation of axonogenesis	10/373	207/19943	0.005757	0.036844	0.0286828	Thy1/Grin1/Nefl/Nefm/Slit1/Nrg1/Map2/Map1b/Trpc5/L1cam	10
BP	GO:0030900	forebrain development	18/373	495/19943	0.005837	0.037001	0.02880497	Kif5a/Drd2/Nefh/Grin1/Nefl/Nefm/Slc32a1/Slc17a6/Atp1a3/Kcnc	18
BP	GO:0033605	positive regulation of catecholamine sec	3/373	20/19943	0.005841	0.037001	0.02880497	Chrna4/Pcp4/Chrnb2	3
BP	GO:0050774	negative regulation of dendrite morphog	3/373	20/19943	0.005841	0.037001	0.02880497	Cit/Dnm3/Trpc5	3
BP	GO:0097107	postsynaptic density assembly	3/373	20/19943	0.005841	0.037001	0.02880497	Nptx1/Cbln1/Shank1	3
BP	GO:1900451	positive regulation of glutamate recepto	3/373	20/19943	0.005841	0.037001	0.02880497	Cacng2/Cacng3/Necab2	3
BP	GO:0086003	cardiac muscle cell contraction	5/373	62/19943	0.006026	0.038062	0.02963057	Ryr2/Scn4b/Scn1a/Akap6/Cacna2d1	5
BP	GO:0030902	hindbrain development	10/373	209/19943	0.006149	0.038782	0.03019107	Grin1/Ckmt1/Sez6l2/Atp2b2/Kcnc1/Cbln1/Klhl1/Cend1/Sptbn2/Pc	10
BP	GO:0031279	regulation of cyclase activity	4/373	40/19943	0.006471	0.040755	0.03172681	Hpca/Drd2/Hrh3/Akap6	4
BP	GO:0043271	negative regulation of ion transport	9/373	179/19943	0.00669	0.041964	0.03266815	Cabp1/Fgf12/Drd2/Hecw1/Kcnab1/Hrh3/Htr2a/Prkce/Oprl1	9
BP	GO:0035584	calcium-mediated signaling using intrace	3/373	21/19943	0.006722	0.041964	0.03266815	Dmtn/Ryr2/Tenm2	3
BP	GO:0045475	locomotor rhythm	3/373	21/19943	0.006722	0.041964	0.03266815	Mapk10/Kcnma1/Oprl1	3
BP	GO:0099188	postsynaptic cytoskeleton organization	3/373	21/19943	0.006722	0.041964	0.03266815	Nefl/Ina/Camk2b	3
BP	GO:0021549	cerebellum development	8/373	148/19943	0.00679	0.041964	0.03266815	Ckmt1/Sez6l2/Atp2b2/Kcnc1/Cbln1/Klhl1/Cend1/Sptbn2	8
BP	GO:0046883	regulation of hormone secretion	13/373	316/19943	0.00682	0.041964	0.03266815	Vsnl1/Scg5/Drd2/Pex5l/Gpld1/Crhbp/Htr2c/Chga/Gnas/Stxbp5l/N	13
BP	GO:0034764	positive regulation of transmembrane tr	11/373	246/19943	0.006836	0.041964	0.03266815	Cacng2/Thy1/Cacng3/Ryr2/Rgs7/Dpp6/Kcnc1/Kcna1/Akap6/Cacna	11
BP	GO:0001661	conditioned taste aversion	2/373	7/19943	0.006885	0.041964	0.03266815	Grin1/Chrnb2	2
BP	GO:0010511	regulation of phosphatidylinositol biosyr	2/373	7/19943	0.006885	0.041964	0.03266815	Htr2c/Htr2a	2
BP	GO:0021578	hindbrain maturation	2/373	7/19943	0.006885	0.041964	0.03266815	Grin1/Cend1	2
BP	GO:0030644	cellular chloride ion homeostasis	2/373	7/19943	0.006885	0.041964	0.03266815	Slc12a5/Abcc2	2
BP	GO:0050975	sensory perception of touch	2/373	7/19943	0.006885	0.041964	0.03266815	Rab3a/Kcna1	2
BP	GO:0051582	positive regulation of neurotransmitter u	2/373	7/19943	0.006885	0.041964	0.03266815	Drd2/Nat8l	2
BP	GO:0098943	neurotransmitter receptor transport, po:	2/373	7/19943	0.006885	0.041964	0.03266815	Cacng2/Cacng3	2
BP	GO:1900028	negative regulation of ruffle assembly	2/373	7/19943	0.006885	0.041964	0.03266815	Pfn2/LOC100909840	2
BP	GO:1903935	response to sodium arsenite	2/373	7/19943	0.006885	0.041964	0.03266815	Nefh/Nefl	2
BP	GO:1990034	calcium ion export across plasma membr	2/373	7/19943	0.006885	0.041964	0.03266815	Atp2b3/Slc8a3	2
BP	GO:1990709	presynaptic active zone organization	2/373	7/19943	0.006885	0.041964	0.03266815	Rab3a/Bsn	2
BP	GO:0010977	negative regulation of neuron projection	9/373	180/19943	0.00693	0.042178	0.03283479	Thy1/Rit2/RGD1307443/Stx1b/Slit1/Cit/Map2/Dnm3/Trpc5	9
BP	GO:0021696	cerebellar cortex morphogenesis	4/373	41/19943	0.007067	0.042955	0.03343982	Atp2b2/Cbln1/Cend1/Sptbn2	4
BP	GO:0097062	dendritic spine maintenance	3/373	22/19943	0.007676	0.046466	0.03617298	Grin1/Zfp804a/Grin2b	3
BP	GO:0099558	maintenance of synapse structure	3/373	22/19943	0.007676	0.046466	0.03617298	Rab3a/Dlg2/Bsn	3
BP	GO:0042745	circadian sleep/wake cycle	4/373	42/19943	0.007698	0.046466	0.03617298	Drd2/Hcrtr2/Kcna2/Chrnb2	4
BP	GO:1903170	negative regulation of calcium ion transr	4/373	42/19943	0.007698	0.046466	0.03617298	Cabp1/Drd2/Prkce/Oprl1	4
BP	GO:0060996	dendritic spine development	7/373	122/19943	0.00809	0.048761	0.0379596	Arhgap44/Slc12a5/Camk2b/Camk2a/Nrg1/Shank1/Dnm3	7
CC	GO:0097060	synaptic membrane	80/373	493/19943	6.57E-52	2.86E-48	2.2283E-48	Grm4/Rph3a/Magee1/Gabrg2/Glra2/Gabra1/Scn8a/Kcnj3/Lrfn5/C	80
CC	GO:0099240	intrinsic component of synaptic membra	53/373	254/19943	4.45E-40	9.69E-37	7.5468E-37	Grm4/Gabrg2/Gabra1/Scn8a/Kcnj3/Lrfn5/Cacng2/Drd2/Slitrk3/Op	53
CC	GO:0045211	postsynaptic membrane	58/373	354/19943	1.17E-37	1.7E-34	1.3214E-34	Rph3a/Magee1/Gabrg2/Glra2/Gabra1/Scn8a/Lrfn5/Cacng2/Cadps	58
CC	GO:0099699	integral component of synaptic membra	49/373	235/19943	4.67E-37	5.09E-34	3.9647E-34	Grm4/Gabrg2/Gabra1/Scn8a/Kcnj3/Lrfn5/Cacng2/Drd2/Slitrk3/Op	49
CC	GO:0099572	postsynaptic specialization	62/373	452/19943	1.35E-35	1.18E-32	9.1577E-33	Cabp1/Rapgef4/Gabrg2/Gabra1/Arhgap44/Nsf/Scn8a/Lrfn5/Cacn	62
CC	GO:0042734	presynaptic membrane	45/373	212/19943	1.97E-34	1.43E-31	1.1115E-31	Grm4/Scn8a/Kcnj3/Cadps2/Drd2/Oprd1/Gabra5/Rims1/Chrna4/C	45
CC	GO:0034702	ion channel complex	50/373	294/19943	2.71E-33	1.69E-30	1.3134E-30	Gabrg2/Glra2/Gabra1/Scn8a/Kcnj3/Cacng2/Gabra5/Chrna4/Pex5l	50
CC	GO:1902495	transmembrane transporter complex	51/373	314/19943	6.13E-33	3.34E-30	2.5978E-30	Gabrg2/Glra2/Gabra1/Scn8a/Kcnj3/Cacng2/Gabra5/Chrna4/Pex5l	51
CC	GO:1990351	transporter complex	51/373	321/19943	1.86E-32	9E-30	7.0093E-30	Gabrg2/Glra2/Gabra1/Scn8a/Kcnj3/Cacng2/Gabra5/Chrna4/Pex5l	51
CC	GO:0032279	asymmetric synapse	55/373	421/19943	1.82E-30	6.62E-28	5.1515E-28	Cabp1/Rapgef4/Arhgap44/Nsf/Scn8a/Lrfn5/Cacng2/Drd2/Nefh/N	55
CC	GO:0098889	intrinsic component of presynaptic mem	35/373	135/19943	3.44E-30	9.99E-28	7.7773E-28	Grm4/Scn8a/Kcnj3/Drd2/Oprd1/Gabra5/Chrna4/Cntnap4/Grin1/R	35
CC	GO:0098984	neuron to neuron synapse	56/373	445/19943	3.7E-30	1.01E-27	7.8511E-28	Cabp1/Rapgef4/Arhgap44/Nsf/Scn8a/Lrfn5/Cacng2/Drd2/Nefh/N	56
CC	GO:0098936	intrinsic component of postsynaptic mer	39/373	185/19943	8.19E-30	2.1E-27	1.6353E-27	Gabrg2/Gabra1/Scn8a/Lrfn5/Cacng2/Drd2/Slitrk3/Oprd1/Gabra5/	39
CC	GO:0014069	postsynaptic density	54/373	417/19943	1.01E-29	2.45E-27	1.9101E-27	Cabp1/Rapgef4/Arhgap44/Nsf/Scn8a/Lrfn5/Cacng2/Drd2/Nefh/N	54
CC	GO:0099056	integral component of presynaptic mem	32/373	122/19943	7.32E-28	1.28E-25	9.9358E-26	Grm4/Scn8a/Kcnj3/Drd2/Oprd1/Gabra5/Chrna4/Cntnap4/Grin1/G	32
CC	GO:0150034	distal axon	52/373	423/19943	1.58E-27	2.65E-25	2.0618E-25	Cplx1/Grm4/Rapgef4/Syt2/Kif5a/Lamp5/Pvalb/Thy1/Drd2/Ncs1/S	52
CC	GO:0099055	integral component of postsynaptic mer	36/373	175/19943	3.44E-27	5.56E-25	4.328E-25	Gabrg2/Gabra1/Scn8a/Lrfn5/Cacng2/Drd2/Slitrk3/Oprd1/Gabra5/	36
CC	GO:0034703	cation channel complex	39/373	221/19943	9.8E-27	1.33E-24	1.0389E-24	Scn8a/Kcnj3/Cacng2/Pex5l/Kcns2/Kcnab2/Grin1/Cacng3/Dlgap3/F	39
CC	GO:0043679	axon terminus	39/373	222/19943	1.17E-26	1.54E-24	1.202E-24	Cplx1/Grm4/Rapgef4/Syt2/Pvalb/Drd2/Ncs1/Syn1/Oprd1/Kcnab2,	39
CC	GO:0044306	neuron projection terminus	40/373	245/19943	4.7E-26	5.69E-24	4.4284E-24	Cplx1/Grm4/Rapgef4/Syt2/Pvalb/Drd2/Ncs1/Syn1/Oprd1/Kcnab2,	40
CC	GO:0098948	intrinsic component of postsynaptic spe	29/373	114/19943	6.25E-25	6.64E-23	5.1724E-23	Gabrg2/Gabra1/Scn8a/Lrfn5/Cacng2/Slitrk3/Oprd1/Gabra5/Clstn	29
CC	GO:0099634	postsynaptic specialization membrane	31/373	139/19943	1.1E-24	1.09E-22	8.4608E-23	Gabrg2/Gabra1/Scn8a/Lrfn5/Cacng2/Slitrk3/Oprd1/Gabra5/Clstn	31
CC	GO:0099060	integral component of postsynaptic spec	27/373	108/19943	4.55E-23	3.54E-21	2.7545E-21	Gabrg2/Gabra1/Scn8a/Lrfn5/Cacng2/Slitrk3/Oprd1/Gabra5/Clstn	27
CC	GO:0098982	GABA-ergic synapse	25/373	106/19943	9.04E-21	6.68E-19	5.1985E-19	Gabrg2/Gabra1/Lrfn5/Drd2/Slitrk3/Gabra5/Rims1/Clstn3/Cntnap	25
CC	GO:0060076	excitatory synapse	21/373	76/19943	3.33E-19	2.34E-17	1.8233E-17	Grm4/Rapgef4/Scn8a/Kcnj3/Cadps2/Grin1/Cacng3/Slc17a6/Calb2	21
CC	GO:0044304	main axon	21/373	86/19943	5.56E-18	3.73E-16	2.9017E-16	Scn8a/Thy1/Kcnab2/Epb41l3/Crhbp/Ank1/Kcna2/Kcnc3/Scn1a/Kc	21
CC	GO:0099146	intrinsic component of postsynaptic den	20/373	81/19943	2.74E-17	1.76E-15	1.3677E-15	Scn8a/Lrfn5/Cacng2/Slitrk3/Oprd1/Clstn3/Grin1/Cacng3/Rgs7bp/	20
CC	GO:0032589	neuron projection membrane	19/373	74/19943	7.79E-17	4.78E-15	3.7247E-15	Gabrg2/Gabra1/Lamp5/Thy1/Hpca/Oprd1/Gabra5/Grin1/Epb41l3,	19
CC	GO:0098839	postsynaptic density membrane	21/373	99/19943	1.25E-16	7.57E-15	5.8905E-15	Scn8a/Lrfn5/Cacng2/Slitrk3/Oprd1/Clstn3/Grin1/Cacng3/Rgs7bp/	21
CC	GO:0043195	terminal bouton	23/373	126/19943	1.46E-16	8.74E-15	6.8041E-15	Cplx1/Grm4/Syt2/Pvalb/Syn1/Grin1/Calb2/Sncb/Vamp1/Rab3a/Pa	23
CC	GO:0099061	integral component of postsynaptic den:	18/373	75/19943	1.89E-15	1.08E-13	8.4282E-14	Scn8a/Lrfn5/Cacng2/Slitrk3/Oprd1/Clstn3/Grin1/Cacng3/Grm1/At	18

CC	GO:0008021	synaptic vesicle	28/373	237/19943	8.37E-15	4.62E-13	3.5965E-13	Rph3a/Syt2/Lamp5/Drd2/Syn1/Oprd1/Slc5a7/Rab3c/Grin1/Slc32a	28
CC	GO:0098688	parallel fiber to Purkinje cell synapse	13/373	33/19943	1.14E-14	6.2E-13	4.8267E-13	Grm4/Scn8a/Kcnj3/Cadps2/Grin1/Calb2/Atp2b2/Atp2b3/Cbln1/Kc	13
CC	GO:0043204	perikaryon	24/373	177/19943	3.34E-14	1.73E-12	1.3506E-12	Rbfox3/Kif5a/Pcsk2/Hpca/Drd2/Nefh/Serpini1/Slc5a7/Rgs7bp/Glr	24
CC	GO:0044309	neuron spine	26/373	217/19943	5.6E-14	2.84E-12	2.2075E-12	Rph3a/Rapgef4/Arhgap44/Hpca/Drd2/Oprd1/Grin1/Rgs7bp/Grm1	26
CC	GO:0031256	leading edge membrane	23/373	171/19943	1.38E-13	6.76E-12	5.2596E-12	Gabrg2/Gabra1/Arhgap44/Lamp5/Thy1/Hpca/Oprd1/Gabra5/Grin	23
CC	GO:0008076	voltage-gated potassium channel compl	17/373	83/19943	1.93E-13	9.16E-12	7.13E-12	Kcnj3/Kcns2/Kcnab2/Kcna2/Kcnc3/Dpp6/Kcnip4/Kcnc1/Kcnab1/Kc	17
CC	GO:0030672	synaptic vesicle membrane	20/373	127/19943	2.69E-13	1.24E-11	9.6199E-12	Rph3a/Syt2/Lamp5/Drd2/Syn1/Oprd1/Rab3c/Slc32a1/Slc17a6/Ga	20
CC	GO:0099501	exocytic vesicle membrane	20/373	127/19943	2.69E-13	1.24E-11	9.6199E-12	Rph3a/Syt2/Lamp5/Drd2/Syn1/Oprd1/Rab3c/Slc32a1/Slc17a6/Ga	20
CC	GO:0043197	dendritic spine	25/373	214/19943	3.09E-13	1.4E-11	1.0924E-11	Rph3a/Rapgef4/Arhgap44/Hpca/Drd2/Oprd1/Grin1/Rgs7bp/Grm1	25
CC	GO:0070382	exocytic vesicle	28/373	280/19943	5.6E-13	2.49E-11	1.9389E-11	Rph3a/Syt2/Lamp5/Drd2/Syn1/Oprd1/Slc5a7/Rab3c/Grin1/Slc32a	28
CC	GO:0048786	presynaptic active zone	17/373	91/19943	9.52E-13	4.07E-11	3.1651E-11	Grm4/Arhgap44/Scn8a/Syn1/Rims1/Slc32a1/Stx1b/Rab3a/Atp2b2	17
CC	GO:0034705	potassium channel complex	17/373	94/19943	1.66E-12	6.75E-11	5.2539E-11	Kcnj3/Kcns2/Kcnab2/Kcna2/Kcnc3/Dpp6/Kcnip4/Kcnc1/Kcnab1/Kc	17
CC	GO:0030133	transport vesicle	32/373	390/19943	2.66E-12	1.07E-10	8.3664E-11	Rph3a/Nptx1/Syt2/Pcsk2/Lamp5/Drd2/Syn1/Oprd1/Slc5a7/Rab3c	32
CC	GO:0032590	dendrite membrane	13/373	48/19943	2.97E-12	1.17E-10	9.088E-11	Gabrg2/Gabra1/Lamp5/Thy1/Hpca/Oprd1/Gabra5/Grin1/Slc12a5/	13
CC	GO:0031594	neuromuscular junction	16/373	99/19943	4.49E-11	1.59E-09	1.2386E-09	Rph3a/Syt2/Slc5a7/Nefl/Nefm/Dlgap3/Stx1b/Vamp1/Kcnc3/Syp/S	16
CC	GO:0030658	transport vesicle membrane	21/373	186/19943	4.87E-11	1.71E-09	1.3337E-09	Rph3a/Syt2/Lamp5/Drd2/Syn1/Oprd1/Rab3c/Slc32a1/Slc17a6/Ga	21
CC	GO:0098685	Schaffer collateral - CA1 synapse	17/373	119/19943	8.39E-11	2.79E-09	2.1718E-09	Cplx1/Cacng2/Nefh/Syn1/Cacng3/Nefl/Grm1/Ina/Syp/Camk2a/Ad	17
CC	GO:0031253	cell projection membrane	26/373	321/19943	4.23E-10	1.27E-08	9.9046E-09	Gabrg2/Gabra1/Lamp5/Thy1/Hpca/Drd2/Oprd1/Gabra5/Grin1/Dr	26
CC	GO:0030659	cytoplasmic vesicle membrane	29/373	397/19943	4.65E-10	1.39E-08	1.0815E-08	Rph3a/Gabrg2/Gabra1/Syt2/Lamp5/Drd2/Cadps/Syn1/Oprd1/Rab	29
CC	GO:0012506	vesicle membrane	29/373	423/19943	2.01E-09	5.73E-08	4.46E-08	Rph3a/Gabrg2/Gabra1/Syt2/Lamp5/Drd2/Cadps/Syn1/Oprd1/Rab	29
CC	GO:0030673	axolemma	8/373	22/19943	3.53E-09	9.63E-08	7.4942E-08	Thy1/Epb41l3/Ank1/Kcnc3/Kcnc1/Kcnh1/Nrg1/Chrna7	8
CC	GO:0043198	dendritic shaft	12/373	67/19943	3.65E-09	9.89E-08	7.6977E-08	Grm4/Nsf/Rgs7bp/Slc12a5/Syndig1/Zfp804a/Grip2/Cacna1b/Htr2	12
CC	GO:0008328	ionotropic glutamate receptor complex	11/373	56/19943	5.93E-09	1.57E-07	1.2192E-07	Cacng2/Grin1/Cacng3/Dlgap3/Olfm3/Nrn1/Grin2b/Lrrc7/Shank1/I	11
CC	GO:0098981	cholinergic synapse	7/373	16/19943	7.49E-09	1.95E-07	1.5216E-07	Rph3a/Chrna4/Nefl/Dlgap3/Chrm2/Chrna7/Chrnb2	7
CC	GO:0060077	inhibitory synapse	8/373	24/19943	7.87E-09	2.04E-07	1.5887E-07	Gabrg2/Rims1/Glra1/Slc32a1/Gad2/Rims2/Bsn/Gabrb3	8
CC	GO:0044224	juxtaparanode region of axon	6/373	10/19943	8.11E-09	2.09E-07	1.6276E-07	Kcnab2/Epb41l3/Kcna2/Kcnab1/Kcna1/Dlg2	6
CC	GO:0098878	neurotransmitter receptor complex	11/373	58/19943	8.77E-09	2.25E-07	1.7499E-07	Cacng2/Grin1/Cacng3/Dlgap3/Olfm3/Nrn1/Grin2b/Lrrc7/Shank1/I	11
CC	GO:0098563	intrinsic component of synaptic vesicle n	11/373	67/19943	4.26E-08	1.03E-06	7.9801E-07	Syt2/Syn1/Oprd1/Rab3c/Slc32a1/Slc17a6/Vamp1/Rab3a/Syp/Sv2l	11
CC	GO:0044305	calyx of Held	8/373	30/19943	5.68E-08	1.34E-06	1.0408E-06	Cplx1/Ncs1/Glra1/Kcna2/Atp1a3/Kcnc1/Kcna1/Unc13c	8
CC	GO:0043194	axon initial segment	7/373	21/19943	7.02E-08	1.65E-06	1.2808E-06	Scn8a/Ank1/Scn1a/Scn2a/Kcnq2/Map2/Lrrc7	7
CC	GO:0031252	cell leading edge	26/373	414/19943	8.23E-08	1.91E-06	1.4854E-06	Gabrg2/Gabra1/Arhgap44/Lamp5/Thy1/Hpca/Oprd1/Gabra5/Grin	26
CC	GO:0097440	apical dendrite	7/373	26/19943	3.66E-07	7.68E-06	5.9772E-06	Kif5a/Rasgrf1/Slc7a10/Grin2b/Map2/Map1b/Hcn1	7
CC	GO:0098802	plasma membrane receptor complex	16/373	185/19943	4.26E-07	8.83E-06	6.8747E-06	Cacng2/Chrna4/Grin1/Cacng3/Grm1/Dlgap3/Olfm3/Nrn1/Gabbr2,	16
CC	GO:0031045	dense core granule	7/373	27/19943	4.87E-07	1E-05	7.7903E-06	Syt2/Cadps/Ncs1/Oprd1/Crhbp/Stxbp5l/Syt7	7
CC	GO:0030426	growth cone	17/373	219/19943	8.58E-07	1.72E-05	1.3418E-05	Rapgef4/Kif5a/Lamp5/Thy1/Nefl/Rasgrf1/Nrsn1/Sncb/Tenm2/Zfp	17
CC	GO:0099569	presynaptic cytoskeleton	5/373	11/19943	9.38E-07	1.86E-05	1.4472E-05	Rims1/Nefl/Nefm/Rims2/Bsn	5
CC	GO:0030427	site of polarized growth	17/373	222/19943	1.04E-06	2.04E-05	1.5849E-05	Rapgef4/Kif5a/Lamp5/Thy1/Nefl/Rasgrf1/Nrsn1/Sncb/Tenm2/Zfp	17
CC	GO:0005883	neurofilament	5/373	12/19943	1.58E-06	3.01E-05	2.3468E-05	Nefh/Nefl/Nefm/Ina/Pcp4	5
CC	GO:1902710	GABA receptor complex	6/373	21/19943	1.76E-06	3.29E-05	2.5621E-05	Gabrg2/Gabra1/Gabra5/Gabra3/Gabbr2/Gabrb3	6
CC	GO:0043235	receptor complex	23/373	399/19943	2.04E-06	3.78E-05	2.9396E-05	Gabrg2/Gabra1/Cacng2/Gabra5/Chrna4/Pex5l/Grin1/Cacng3/Grm	23
CC	GO:0030285	integral component of synaptic vesicle m	8/373	47/19943	2.31E-06	4.25E-05	3.3072E-05	Syt2/Oprd1/Slc32a1/Slc17a6/Vamp1/Syp/Sv2b/Sv2c	8
CC	GO:0032809	neuronal cell body membrane	7/373	35/19943	3.24E-06	5.65E-05	4.3976E-05	Thy1/Hpca/Gabra5/Kcna2/Kcnc3/Atp2b2/Kcnc1	7
CC	GO:0001518	voltage-gated sodium channel complex	5/373	14/19943	3.88E-06	6.71E-05	5.2268E-05	Scn8a/Scn4b/Scn1a/Scn2a/Scn9a	5
CC	GO:0044298	cell body membrane	7/373	37/19943	4.8E-06	8.21E-05	6.3918E-05	Thy1/Hpca/Gabra5/Kcna2/Kcnc3/Atp2b2/Kcnc1	7
CC	GO:0120111	neuron projection cytoplasm	10/373	90/19943	7.03E-06	0.000117	9.1423E-05	Kif5a/Hpca/Dync1i1/Nefl/Kcnab1/Camk2a/Kif5c/Map2/Dlg2/Bsn	10
CC	GO:0031225	anchored component of membrane	12/373	132/19943	7.15E-06	0.000119	9.2591E-05	Cntn4/Cntn3/Thy1/Syn1/Rab3c/Rgs7bp/Gad2/Rab3a/Nrn1/Cntn5	12
CC	GO:0034707	chloride channel complex	7/373	42/19943	1.16E-05	0.00019	0.00014761	Gabrg2/Glra2/Gabra1/Gabra5/Glra1/Gabra3/Gabrb3	7
CC	GO:0034704	calcium channel complex	8/373	60/19943	1.52E-05	0.000242	0.00018836	Cacng2/Ryr2/Cacna1b/Akap6/Cacna2d2/Trpc5/Cacna2d1/Cacna1i	8
CC	GO:1902711	GABA-A receptor complex	5/373	19/19943	2.09E-05	0.00032	0.00024894	Gabrg2/Gabra1/Gabra5/Gabra3/Gabrb3	5
CC	GO:0042383	sarcolemma	13/373	171/19943	2.09E-05	0.00032	0.00024894	Kcnj3/Rtn2/Ryr2/Ank1/Atp1a3/Scn1a/Gnas/Kcnj12/Akap6/Scn2a/	13
CC	GO:0048787	presynaptic active zone membrane	7/373	47/19943	2.5E-05	0.000371	0.00028852	Grm4/Scn8a/Stx1b/Atp2b2/Kcnma1/Nrg1/Cacna2d1	7
CC	GO:0043083	synaptic cleft	5/373	20/19943	2.74E-05	0.000405	0.0003152	Nptx1/Grin1/Cbln1/Grin2b/Dnm3	5
CC	GO:0098945	intrinsic component of presynaptic activ	6/373	33/19943	2.97E-05	0.000436	0.00033937	Grm4/Scn8a/Atp2b2/Kcnma1/Nrg1/Cacna2d1	6
CC	GO:0098686	hippocampal mossy fiber to CA3 synapse	7/373	49/19943	3.31E-05	0.00048	0.00037384	Rapgef4/Cacng2/Grin1/Adcy1/Grin2b/Syt7/Bsn	7
CC	GO:0098831	presynaptic active zone cytoplasmic con	5/373	21/19943	3.54E-05	0.000511	0.00039761	Rims1/Rims2/Erc2/Bsn/Unc13c	5
CC	GO:0099571	postsynaptic cytoskeleton	5/373	21/19943	3.54E-05	0.000511	0.00039761	Nefh/Nefl/Nefm/Ina/Sptbn2	5
CC	GO:0032839	dendrite cytoplasm	6/373	34/19943	3.55E-05	0.000511	0.00039761	Kif5a/Hpca/Kcnab1/Camk2a/Kif5c/Map2	6
CC	GO:0030315	T-tubule	8/373	69/19943	4.3E-05	0.000594	0.00046262	Kcnj3/Rtn2/Scn1a/Kcnj12/Akap6/Scn2a/Prkce/Cacna2d1	8
CC	GO:0017146	NMDA selective glutamate receptor con	4/373	12/19943	5.29E-05	0.000714	0.00055593	Grin1/Dlgap3/Grin2b/Shank1	4
CC	GO:0034706	sodium channel complex	5/373	24/19943	7.07E-05	0.000933	0.00072635	Scn8a/Scn4b/Scn1a/Scn2a/Scn9a	5
CC	GO:0098690	glycinergic synapse	4/373	13/19943	7.53E-05	0.000977	0.00076057	Glra2/Glra1/Slc6a5/Chrna7	4
CC	GO:0016528	sarcoplasm	8/373	78/19943	0.000104	0.0013	0.0010119	Rtn2/Ryr2/Ank1/Camk2b/Jph3/Akap6/Slc8a3/Cacna2d1	8
CC	GO:0048788	cytoskeleton of presynaptic active zone	3/373	6/19943	0.000124	0.001512	0.00117741	Rims1/Rims2/Bsn	3
CC	GO:0099059	integral component of presynaptic active	5/373	28/19943	0.000154	0.001825	0.00142082	Grm4/Scn8a/Atp2b2/Kcnma1/Nrg1	5
CC	GO:0044327	dendritic spine head	4/373	16/19943	0.000183	0.002154	0.00167707	Hpca/Rgs7bp/Atp1a3/Dnm3	4
CC	GO:0046658	anchored component of plasma membra	7/373	65/19943	0.000207	0.002397	0.00186633	Cntn3/Thy1/Rgs7bp/Nrn1/Cntn5/Dlg2/Cacna2d1	7
CC	GO:0014731	spectrin-associated cytoskeleton	3/373	7/19943	0.000215	0.002437	0.00189682	Dmtn/Ank1/Sptbn2	3
CC	GO:0032281	AMPA glutamate receptor complex	5/373	30/19943	0.000216	0.002437	0.00189682	Cacng2/Cacng3/Olfm3/Nrn1/Vwc2l	5
CC	GO:0033268	node of Ranvier	4/373	18/19943	0.000299	0.003278	0.00255172	Scn8a/Scn1a/Scn2a/Kcnq2	4
CC	GO:0030141	secretory granule	18/373	382/19943	0.000336	0.003614	0.00281313	Rph3a/Syt2/Pcsk2/Scg5/Drd2/Cadps/Ncs1/Serpini1/Oprd1/Ica1l/C	18
CC	GO:0099144	anchored component of synaptic memb	4/373	19/19943	0.000374	0.003933	0.00306165	Rgs7bp/Cntn5/Dlg2/Cacna2d1	4
CC	GO:0044292	dendrite terminus	4/373	20/19943	0.00046	0.004763	0.00370821	Slc32a1/Rgs7/Map2/L1cam	4
CC	GO:1904115	axon cytoplasm	6/373	55/19943	0.00055	0.005551	0.00432169	Kif5a/Dync1i1/Nefl/Kif5c/Dlg2/Bsn	6
CC	GO:0030863	cortical cytoskeleton	9/373	128/19943	0.000687	0.006757	0.00526022	Pvalb/Rims1/Dmtn/Calb2/Ank1/Cap2/Rims2/Bsn/Sptbn2	9
CC	GO:0099025	anchored component of postsynaptic m	3/373	10/19943	0.000706	0.00684	0.00532521	Rgs7bp/Dlg2/Cacna2d1	3
CC	GO:0031300	intrinsic component of organelle membr	13/373	245/19943	0.000752	0.007218	0.00561898	Syt2/Gdap1/Syn1/Oprd1/Rab3c/Slc32a1/Slc17a6/Vamp1/Rab3a/S	13
CC	GO:0005891	voltage-gated calcium channel complex	5/373	39/19943	0.00076	0.007284	0.00567022	Cacng2/Cacna1b/Cacna2d2/Cacna2d1/Cacna1i	5
CC	GO:0098992	neuronal dense core vesicle	3/373	12/19943	0.001259	0.011088	0.0086317	Cadps/Oprd1/Stxbp5l	3
CC	GO:0099523	presynaptic cytosol	4/373	26/19943	0.0013	0.01126	0.00876588	Ncs1/Sncb/Camk2a/Prkce	4
CC	GO:0045121	membrane raft	17/373	396/19943	0.00136	0.011759	0.00915441	Thy1/Oprd1/Slc5a7/Asah2/Rit2/Rgs7/Atp2b2/Atp2b3/Gnas/Akap	17
CC	GO:0098857	membrane microdomain	17/373	397/19943	0.001397	0.012057	0.00938584	Thy1/Oprd1/Slc5a7/Asah2/Rit2/Rgs7/Atp2b2/Atp2b3/Gnas/Akap	17
CC	GO:0016529	sarcoplasmic reticulum	6/373	66/19943	0.001452	0.012484	0.00971848	Rtn2/Ryr2/Ank1/Camk2b/Akap6/Cacna2d1	6
CC	GO:0099026	anchored component of presynaptic me	3/373	13/19943	0.001615	0.013584	0.01057497	Rgs7bp/Cntn5/Cacna2d1	3
CC	GO:0098589	membrane region	17/373	411/19943	0.002014	0.016326	0.01270915	Thy1/Oprd1/Slc5a7/Asah2/Rit2/Rgs7/Atp2b2/Atp2b3/Gnas/Akap	17
CC	GO:0044448	cell cortex part	11/373	212/19943	0.002239	0.01787	0.01391128	Pvalb/Rims1/Dmtn/Calb2/Ank1/Cap2/Rims2/Erc2/Bsn/Unc13c/Sp	11
CC	GO:0032588	trans-Golgi network membrane	4/373	31/19943	0.002542	0.019744	0.01537005	Lgr5/Cabp7/Caln1/Gnas	4
CC	GO:0044853	plasma membrane raft	8/373	127/19943	0.002702	0.020698	0.01611287	Slc5a7/Asah2/Akap6/Kcnma1/Htr2a/Chrna7/Add2/Chrnb2	8
CC	GO:0005892	acetylcholine-gated channel complex	3/373	16/19943	0.003033	0.022438	0.01746794	Chrna4/Chrna7/Chrnb2	3
CC	GO:0098691	dopaminergic synapse	3/373	16/19943	0.003033	0.022438	0.01746794	Drd2/Chrna4/Chrnb2	3
CC	GO:0044307	dendritic branch	2/373	5/19943	0.003361	0.02413	0.01878472	Grin1/Map2	2
CC	GO:0044316	cone cell pedicle	2/373	5/19943	0.003361	0.02413	0.01878472	Rapgef4/Slc32a1	2
CC	GO:0097418	neurofibrillary tangle	2/373	5/19943	0.003361	0.02413	0.01878472	Nefh/Nefm	2
CC	GO:0099738	cell cortex region	5/373	55/19943	0.003602	0.025399	0.01977238	Rims1/Rims2/Erc2/Bsn/Unc13c	5
CC	GO:0033270	paranode region of axon	3/373	17/19943	0.003632	0.025487	0.01984144	Epb41l3/Kcna1/Scn2a	3
CC	GO:0097444	spine apparatus	2/373	6/19943	0.004979	0.032778	0.0255171	Oprd1/Chrna7	2
CC	GO:0099030	anchored component of postsynaptic sp	2/373	6/19943	0.004979	0.032778	0.0255171	Rgs7bp/Dlg2	2
CC	GO:0099031	anchored component of postsynaptic de	2/373	6/19943	0.004979	0.032778	0.0255171	Rgs7bp/Dlg2	2
CC	GO:0000139	Golgi membrane	17/373	453/19943	0.005373	0.034592	0.02692929	Cabp1/Lgr5/Camk1g/Clstn3/Asah2/Gad2/Tmem59l/Galnt9/Cabp7	17
CC	GO:0019898	extrinsic component of membrane	13/373	309/19943	0.005689	0.036458	0.02838196	Rph3a/Hpca/Cadps/Syn1/Kcnab2/Ryr2/Cdh12/Tenm2/Cdh7/Kcna	13
CC	GO:0098993	anchored component of synaptic vesicle	3/373	20/19943	0.005841	0.037001	0.02880497	Syn1/Rab3c/Rab3a	3
CC	GO:0032838	plasma membrane bounded cell projecti	10/373	208/19943	0.005951	0.03764	0.02930186	Kif5a/Hpca/Dync1i1/Nefl/Kcnab1/Camk2a/Kif5c/Map2/Dlg2/Bsn	10

CC	GO:0099522	region of cytosol	4/373	42/19943	0.007698	0.046466	0.03617298	Ncs1/Sncb/Camk2a/Prkce	4
CC	GO:0031301	integral component of organelle membr	10/373	218/19943	0.008175	0.04921	0.03830922	Syt2/Gdap1/Oprd1/Slc32a1/Slc17a6/Vamp1/Syp/Sv2b/Sv2c/Chrn	10
MF	GO:0022839	ion gated channel activity	48/373	326/19943	5.05E-29	1.05E-26	8.1634E-27	Gabrg2/Glra2/Gabra1/Scn8a/Kcnj3/Cacng2/Kcnab3/Ncs1/Gabra5/	48
MF	GO:0022836	gated channel activity	48/373	337/19943	2.35E-28	4.46E-26	3.4711E-26	Gabrg2/Glra2/Gabra1/Scn8a/Kcnj3/Cacng2/Kcnab3/Ncs1/Gabra5/	48
MF	GO:0005216	ion channel activity	50/373	406/19943	1.6E-26	2.05E-24	1.5989E-24	Gabrg2/Glra2/Gabra1/Scn8a/Kcnj3/Cacng2/Slc24a2/Kcnab3/Ncs1	50
MF	GO:0022838	substrate-specific channel activity	50/373	417/19943	5.56E-26	6.55E-24	5.0976E-24	Gabrg2/Glra2/Gabra1/Scn8a/Kcnj3/Cacng2/Slc24a2/Kcnab3/Ncs1	50
MF	GO:0005261	cation channel activity	43/373	317/19943	1.35E-24	1.26E-22	9.8249E-23	Scn8a/Kcnj3/Cacng2/Slc24a2/Kcnab3/Ncs1/Chrna4/Pex5l/Kcns2/	43
MF	GO:0015267	channel activity	50/373	447/19943	1.36E-24	1.26E-22	9.8249E-23	Gabrg2/Glra2/Gabra1/Scn8a/Kcnj3/Cacng2/Slc24a2/Kcnab3/Ncs1	50
MF	GO:0022803	passive transmembrane transporter acti	50/373	447/19943	1.36E-24	1.26E-22	9.8249E-23	Gabrg2/Glra2/Gabra1/Scn8a/Kcnj3/Cacng2/Slc24a2/Kcnab3/Ncs1	50
MF	GO:0005244	voltage-gated ion channel activity	34/373	195/19943	3E-23	2.38E-21	1.852E-21	Scn8a/Kcnj3/Cacng2/Kcnab3/Ncs1/Kcns2/Kcnab2/Grin1/Cacng3/	34
MF	GO:0022832	voltage-gated channel activity	34/373	195/19943	3E-23	2.38E-21	1.852E-21	Scn8a/Kcnj3/Cacng2/Kcnab3/Ncs1/Kcns2/Kcnab2/Grin1/Cacng3/	34
MF	GO:0046873	metal ion transmembrane transporter a	47/373	430/19943	1.01E-22	7.72E-21	6.01E-21	Scn8a/Kcnj3/Cacng2/Slc24a2/Kcnab3/Ncs1/Slc5a7/Kcns2/Kcnab2/	47
MF	GO:0022843	voltage-gated cation channel activity	27/373	146/19943	2.2E-19	1.57E-17	1.2255E-17	Kcnj3/Cacng2/Kcnab3/Ncs1/Kcns2/Kcnab2/Grin1/Cacng3/Kcna2/	27
MF	GO:0035254	glutamate receptor binding	19/373	67/19943	9.9E-18	6.54E-16	5.0902E-16	Cabp1/Nsf/Cacng2/Drd2/Grin1/Cacng3/Rasgrf1/Atp2b2/Syndig1/	19
MF	GO:0015079	potassium ion transmembrane transpor	24/373	161/19943	3.81E-15	2.15E-13	1.6775E-13	Kcnj3/Slc24a2/Kcnab3/Kcns2/Kcnab2/Slc12a5/Kcna2/Kcnc3/Atp1a	24
MF	GO:0015077	monovalent inorganic cation transmemb	34/373	375/19943	2.99E-14	1.57E-12	1.2242E-12	Scn8a/Kcnj3/Slc24a2/Kcnab3/Slc5a7/Kcns2/Kcnab2/Slc32a1/Slc12	34
MF	GO:0005249	voltage-gated potassium channel activit	18/373	88/19943	3.83E-14	1.96E-12	1.5278E-12	Kcnj3/Kcnab3/Kcns2/Kcnab2/Kcna2/Kcnc3/Kcnc1/Kcnab1/Kcna1/	18
MF	GO:0030594	neurotransmitter receptor activity	20/373	119/19943	7.51E-14	3.72E-12	2.894E-12	Gabrg2/Glra2/Gabra1/Drd2/Gabra5/Chrna4/Grin1/Glra1/Grm1/H	20
MF	GO:0005267	potassium channel activity	20/373	127/19943	2.69E-13	1.24E-11	9.6199E-12	Kcnj3/Kcnab3/Kcns2/Kcnab2/Kcna2/Kcnc3/Kcnip4/Kcnc1/Kcnab1/	20
MF	GO:0015276	ligand-gated ion channel activity	20/373	131/19943	4.92E-13	2.21E-11	1.7218E-11	Gabrg2/Glra2/Gabra1/Kcnj3/Gabra5/Chrna4/Pex5l/Grin1/Glra1/R	20
MF	GO:0022834	ligand-gated channel activity	20/373	134/19943	7.63E-13	3.32E-11	2.5876E-11	Gabrg2/Glra2/Gabra1/Kcnj3/Gabra5/Chrna4/Pex5l/Grin1/Glra1/R	20
MF	GO:0098918	structural constituent of synapse	11/373	30/19943	3.36E-12	1.3E-10	1.0094E-10	Nefh/Rims1/Nefl/Ina/Camk2b/Rims2/Erc2/Dlg2/Dnm3/Bsn/Sptbn	11
MF	GO:0098960	postsynaptic neurotransmitter receptor	13/373	57/19943	3.24E-11	1.19E-09	9.2409E-10	Gabrg2/Glra2/Gabra1/Gabra5/Chrna4/Grin1/Glra1/Grm1/Gabra3	13
MF	GO:0005516	calmodulin binding	20/373	169/19943	6.01E-11	2.08E-09	1.6187E-09	Grm4/Camk1g/Grin1/Ryr2/Rit2/Atp2b2/Camk2b/Atp2b3/Pcp4/Ca	20
MF	GO:0042165	neurotransmitter binding	13/373	61/19943	8.13E-11	2.75E-09	2.137E-09	Glra2/Slc5a7/Chrna4/Grin1/Glra1/Crhbp/Htr2c/Slc6a11/Grin2b/H	13
MF	GO:1904315	transmitter-gated ion channel activity in	12/373	52/19943	1.62E-10	5.18E-09	4.0356E-09	Gabrg2/Glra2/Gabra1/Gabra5/Chrna4/Grin1/Glra1/Gabra3/Grin2	12
MF	GO:0099529	neurotransmitter receptor activity involv	12/373	55/19943	3.27E-10	1.01E-08	7.8677E-09	Gabrg2/Glra2/Gabra1/Gabra5/Chrna4/Grin1/Glra1/Gabra3/Grin2	12
MF	GO:0035255	ionotropic glutamate receptor binding	11/373	44/19943	3.73E-10	1.13E-08	8.7957E-09	Nsf/Cacng2/Drd2/Cacng3/Gnas/Grin2b/Lrrc7/Neto1/Neto2/Shank	11
MF	GO:0022824	transmitter-gated ion channel activity	12/373	58/19943	6.32E-10	1.86E-08	1.4467E-08	Gabrg2/Glra2/Gabra1/Gabra5/Chrna4/Grin1/Glra1/Gabra3/Grin2	12
MF	GO:0022835	transmitter-gated channel activity	12/373	58/19943	6.32E-10	1.86E-08	1.4467E-08	Gabrg2/Glra2/Gabra1/Gabra5/Chrna4/Grin1/Glra1/Gabra3/Grin2	12
MF	GO:0015085	calcium ion transmembrane transporter	16/373	134/19943	4.46E-09	1.19E-07	9.2805E-08	Cacng2/Slc24a2/Ncs1/Grin1/Cacng3/Ryr2/Atp2b2/Atp2b3/Jph3/C	16
MF	GO:0005230	extracellular ligand-gated ion channel ac	12/373	70/19943	6.16E-09	1.62E-07	1.2594E-07	Gabrg2/Glra2/Gabra1/Gabra5/Chrna4/Grin1/Glra1/Gabra3/Grin2	12
MF	GO:0099095	ligand-gated anion channel activity	7/373	20/19943	4.76E-08	1.14E-06	8.8687E-07	Gabrg2/Glra2/Gabra1/Gabra5/Glra1/Gabra3/Gabrb3	7
MF	GO:0070405	ammonium ion binding	11/373	79/19943	2.45E-07	5.36E-06	4.17E-06	Drd2/Slc5a7/Chrna4/Htr2c/Gpr12/Rasgrp1/Htr2a/Chrna7/Pcyt1b/	11
MF	GO:0099094	ligand-gated cation channel activity	12/373	99/19943	3.29E-07	7.03E-06	5.4728E-06	Kcnj3/Chrna4/Pex5l/Grin1/Ryr2/Jph3/Kcnj12/Kcnh7/Grin2b/Chrn	12
MF	GO:0005262	calcium channel activity	13/373	119/19943	3.64E-07	7.66E-06	5.9603E-06	Cacng2/Slc24a2/Ncs1/Grin1/Cacng3/Ryr2/Jph3/Cacna1b/Grin2b/	13
MF	GO:0016247	channel regulator activity	14/373	141/19943	4.24E-07	8.83E-06	6.8747E-06	Fgf12/Lynx1/Cacng2/Kcnab2/Cacng3/Gpld1/Scn4b/Dpp6/Kcnip4/	14
MF	GO:0005237	inhibitory extracellular ligand-gated ion	6/373	17/19943	4.28E-07	8.83E-06	6.8747E-06	Gabrg2/Glra2/Gabra1/Gabra5/Glra1/Gabra3	6
MF	GO:0005251	delayed rectifier potassium channel acti	7/373	29/19943	8.28E-07	1.67E-05	1.3012E-05	Kcna2/Kcnc3/Kcnc1/Kcna1/Kcnh1/Kcnq2/Kcng1	7
MF	GO:0005248	voltage-gated sodium channel activity	6/373	20/19943	1.28E-06	2.46E-05	1.9168E-05	Scn8a/Scn4b/Scn1a/Scn2a/Scn9a/Hcn1	6
MF	GO:0099186	structural constituent of postsynapse	6/373	20/19943	1.28E-06	2.46E-05	1.9168E-05	Nefh/Nefl/Ina/Camk2b/Dlg2/Dnm3	6
MF	GO:0099508	voltage-gated ion channel activity involv	4/373	6/19943	1.75E-06	3.29E-05	2.5621E-05	Kcnc1/Kcna1/Kcnh1/Scn2a	4
MF	GO:0016917	GABA receptor activity	6/373	22/19943	2.38E-06	4.34E-05	3.3804E-05	Gabrg2/Gabra1/Gabra5/Gabra3/Gabbr2/Gabrb3	6
MF	GO:0022851	GABA-gated chloride ion channel activity	5/373	13/19943	2.53E-06	4.58E-05	3.565E-05	Gabrg2/Gabra1/Gabra5/Gabra3/Gabrb3	5
MF	GO:0015081	sodium ion transmembrane transporter	13/373	141/19943	2.54E-06	4.58E-05	3.565E-05	Scn8a/Slc24a2/Slc5a7/Atp1a3/Scn4b/Slc6a5/Scn1a/Slc6a11/Scn2a	13
MF	GO:0030165	PDZ domain binding	12/373	121/19943	2.88E-06	5.13E-05	3.9913E-05	Nsf/Cacng3/Dlgap3/Lin7b/Atp2b2/Cxxc4/Atp2b3/Cit/Kcnj12/Erc2/	12
MF	GO:0044325	ion channel binding	13/373	143/19943	2.98E-06	5.27E-05	4.1063E-05	Cabp1/Fgf12/Rims1/Kcnab2/Ank1/Scn4b/Kcnc1/Kcnab1/Dpp10/K	13
MF	GO:0098882	structural constituent of presynaptic act	4/373	7/19943	4.03E-06	6.94E-05	5.4058E-05	Rims1/Rims2/Erc2/Bsn	4
MF	GO:0005543	phospholipid binding	23/373	424/19943	5.59E-06	9.49E-05	7.3857E-05	Rph3a/Arhgap44/Syt2/Thy1/Cadps/Syt12/Pla2g4e/Pacsin1/Cpne4	23
MF	GO:0099181	structural constituent of presynapse	4/373	9/19943	1.41E-05	0.000225	0.00017506	Rims1/Rims2/Erc2/Bsn	4
MF	GO:0004890	GABA-A receptor activity	5/373	19/19943	2.09E-05	0.00032	0.00024894	Gabrg2/Gabra1/Gabra5/Gabra3/Gabrb3	5
MF	GO:0099528	G protein-coupled neurotransmitter rece	7/373	47/19943	2.5E-05	0.000371	0.00028852	Grm1/Htr2c/Chrm2/Hrh3/Gabbr2/Htr2a/Htr4	7
MF	GO:0008503	benzodiazepine receptor activity	4/373	11/19943	3.58E-05	0.000513	0.00039917	Gabrg2/Gabra1/Gabra5/Gabra3	4
MF	GO:0005245	voltage-gated calcium channel activity	7/373	50/19943	3.78E-05	0.000535	0.00041667	Cacng2/Ncs1/Cacng3/Cacna1b/Cacna2d2/Cacna2d1/Cacna1i	7
MF	GO:0005231	excitatory extracellular ligand-gated ion	6/373	35/19943	4.22E-05	0.000586	0.00045582	Glra2/Chrna4/Glra1/Grin2b/Chrna7/Chrnb2	6
MF	GO:0015464	acetylcholine receptor activity	5/373	22/19943	4.51E-05	0.000617	0.00048009	Chrna4/Chrm2/Hrh3/Chrna7/Chrnb2	5
MF	GO:0000149	SNARE binding	10/373	115/19943	6.06E-05	0.000816	0.00063494	Cplx1/Nsf/Napb/Syt2/Stx1b/Syt12/Syp/Stxbp5l/Syt7/Unc13c	10
MF	GO:0031402	sodium ion binding	4/373	13/19943	7.53E-05	0.000977	0.00076057	Scn8a/Scn1a/Scn2a/Scn9a	4
MF	GO:0005272	sodium channel activity	6/373	39/19943	7.96E-05	0.001027	0.00079919	Scn8a/Scn4b/Scn1a/Scn2a/Scn9a/Hcn1	6
MF	GO:0015108	chloride transmembrane transporter act	9/373	97/19943	8.54E-05	0.001094	0.00085203	Gabrg2/Glra2/Gabra1/Gabra5/Glra1/Slc12a5/Gabra3/Slc12a3/Ga	9
MF	GO:0005200	structural constituent of cytoskeleton	7/373	58/19943	0.0001	0.001265	0.00098509	Nefh/Nefl/Ina/Tuba8/Camk2b/Add2/Sptbn2	7
MF	GO:0019905	syntaxin binding	8/373	79/19943	0.000114	0.001419	0.00110466	Cplx1/Nsf/Napb/Syt2/Syp/Stxbp5l/Syt7/Unc13c	8
MF	GO:0004683	calmodulin-dependent protein kinase ac	5/373	27/19943	0.000128	0.001551	0.00120764	Camk1g/Camk2b/Camk2a/Camk1d/Camk4	5
MF	GO:0008022	protein C-terminus binding	14/373	234/19943	0.00014	0.001683	0.00131033	Rbfox1/Nsf/Nefl/Rasgrf1/Rab3a/Atp2b2/Grip2/Cacna1b/Sgtb/Lrrc	14
MF	GO:0016594	glycine binding	4/373	16/19943	0.000183	0.002154	0.00167707	Glra2/Grin1/Glra1/Grin2b	4
MF	GO:1902936	phosphatidylinositol bisphosphate bindi	9/373	108/19943	0.000195	0.002275	0.0017711	Rph3a/Cadps/Kcnh1/Clvs1/Pfn2/LOC100909840/Amer3/Syt7/Hcn	9
MF	GO:0099106	ion channel regulator activity	9/373	115/19943	0.000313	0.003386	0.00263607	Lynx1/Cacng2/Kcnab2/Dpp6/Kcnip4/Kcnab1/Dpp10/Cacna2d2/Nr	9
MF	GO:0005254	chloride channel activity	7/373	70/19943	0.00033	0.003557	0.00276887	Gabrg2/Glra2/Gabra1/Gabra5/Glra1/Gabra3/Gabrb3	7
MF	GO:0005544	calcium-dependent phospholipid binding	6/373	52/19943	0.000405	0.004233	0.00329545	Rph3a/Syt2/Syt12/Pla2g4e/Cpne4/Syt7	6
MF	GO:0005326	neurotransmitter transporter activity	6/373	53/19943	0.00045	0.004677	0.0036409	Cplx1/Slc5a7/Slc32a1/Slc17a6/Slc6a5/Slc6a11	6
MF	GO:1990763	arrestin family protein binding	3/373	9/19943	0.000501	0.005141	0.00400232	Gpr61/Chrm2/Gpr135	3
MF	GO:0097110	scaffold protein binding	7/373	75/19943	0.000504	0.005157	0.00401467	Kif5a/Dlgap3/Ryr2/Cit/Grin2b/Chrna7/Shank1	7
MF	GO:0005546	phosphatidylinositol-4,5-bisphosphate b	7/373	76/19943	0.000546	0.005526	0.00430161	Rph3a/Cadps/Pfn2/LOC100909840/Amer3/Syt7/Hcn1	7
MF	GO:0031420	alkali metal ion binding	4/373	21/19943	0.00056	0.005611	0.0043683	Scn8a/Scn1a/Scn2a/Scn9a	4
MF	GO:0032947	protein-containing complex scaffold acti	4/373	21/19943	0.00056	0.005611	0.0043683	Mapk8ip2/Grip2/Ksr2/Shank1	4
MF	GO:0008509	anion transmembrane transporter activi	15/373	311/19943	0.000815	0.007724	0.00601319	Gabrg2/Glra2/Gabra1/Gabra5/Glra1/Slc32a1/Slc17a6/Slc12a5/Slc	15
MF	GO:0005253	anion channel activity	7/373	83/19943	0.000928	0.008665	0.00674519	Gabrg2/Glra2/Gabra1/Gabra5/Glra1/Gabra3/Gabrb3	7
MF	GO:0004993	G protein-coupled serotonin receptor ac	5/373	41/19943	0.00096	0.008771	0.00682815	Htr2c/Chrm2/Hrh3/Htr2a/Htr4	5
MF	GO:0099589	serotonin receptor activity	5/373	41/19943	0.00096	0.008771	0.00682815	Htr2c/Chrm2/Hrh3/Htr2a/Htr4	5
MF	GO:0043177	organic acid binding	12/373	223/19943	0.001053	0.009578	0.00745634	Glra2/Grin1/Glra1/Ryr2/Gad2/Acan/Hapln1/Hapln4/Ddc/Grin2b/	12
MF	GO:0015103	inorganic anion transmembrane transpo	9/373	136/19943	0.001059	0.009594	0.00746894	Gabrg2/Glra2/Gabra1/Gabra5/Glra1/Slc12a5/Gabra3/Slc12a3/Ga	9
MF	GO:0035256	G protein-coupled glutamate receptor bi	3/373	12/19943	0.001259	0.011088	0.0086317	Cabp1/Necab2/Dnm3	3
MF	GO:0050780	dopamine receptor binding	4/373	26/19943	0.0013	0.01126	0.00876588	Nsf/Atp1a3/Gnas/Grin2b	4
MF	GO:0099604	ligand-gated calcium channel activity	4/373	26/19943	0.0013	0.01126	0.00876588	Grin1/Ryr2/Jph3/Grin2b	4
MF	GO:0017075	syntaxin-1 binding	4/373	27/19943	0.001503	0.012796	0.00996183	Cplx1/Nsf/Syp/Unc13c	4
MF	GO:0008066	glutamate receptor activity	4/373	28/19943	0.001728	0.014374	0.0111902	Grm4/Grin1/Grm1/Grin2b	4
MF	GO:0015643	toxic substance binding	3/373	14/19943	0.002027	0.016326	0.01270915	Nefh/Nefm/Chrna7	3
MF	GO:0016595	glutamate binding	3/373	14/19943	0.002027	0.016326	0.01270915	Grin1/Gad2/Grin2b	3
MF	GO:0031748	D1 dopamine receptor binding	3/373	14/19943	0.002027	0.016326	0.01270915	Nsf/Atp1a3/Gnas	3
MF	GO:0016597	amino acid binding	6/373	71/19943	0.002118	0.016996	0.01323139	Glra2/Grin1/Glra1/Gad2/Ddc/Grin2b	6
MF	GO:0031406	carboxylic acid binding	11/373	211/19943	0.002158	0.017286	0.0134572	Glra2/Grin1/Glra1/Gad2/Acan/Hapln1/Hapln4/Ddc/Grin2b/Ogfod	11
MF	GO:0022804	active transmembrane transporter activi	15/373	346/19943	0.002328	0.018517	0.01441481	Slc24a2/Slc5a7/Slc32a1/Slc17a6/Slc12a5/Atp1a3/Atp2b2/Slc6a5/	15
MF	GO:0015459	potassium channel regulator activity	5/373	50/19943	0.002367	0.018752	0.01459826	Kcnab2/Dpp6/Kcnip4/Kcnab1/Dpp10	5
MF	GO:0008179	adenylate cyclase binding	3/373	15/19943	0.002498	0.019478	0.01516308	Cap2/Akap6/Chrna7	3
MF	GO:0015293	symporter activity	8/373	128/19943	0.002837	0.021575	0.01679596	Slc24a2/Slc5a7/Slc32a1/Slc17a6/Slc12a5/Slc6a5/Slc6a11/Slc12a3	8
MF	GO:0005217	intracellular ligand-gated ion channel act	4/373	32/19943	0.002862	0.021619	0.01683001	Pex5l/Ryr2/Jph3/Hcn1	4
MF	GO:0005416	amino acid:cation symporter activity	3/373	16/19943	0.003033	0.022438	0.01746794	Slc32a1/Slc6a5/Slc6a11	3
MF	GO:0022848	acetylcholine-gated cation-selective cha	3/373	16/19943	0.003033	0.022438	0.01746794	Chrna4/Chrna7/Chrnb2	3
MF	GO:0042166	acetylcholine binding	3/373	16/19943	0.003033	0.022438	0.01746794	Chrna4/Chrna7/Chrnb2	3
MF	GO:0008227	G protein-coupled amine receptor activi	6/373	77/19943	0.003194	0.02343	0.01823983	Drd2/Htr2c/Chrm2/Hrh3/Htr2a/Htr4	6
MF	GO:0015185	gamma-aminobutyric acid transmembra	2/373	5/19943	0.003361	0.02413	0.01878472	Slc32a1/Slc6a11	2

MF	GO:0016933	extracellularly glycine-gated ion channel	2/373	5/19943	0.003361	0.02413	0.01878472	Glra2/Glra1	2
MF	GO:0016934	extracellularly glycine-gated chloride cha	2/373	5/19943	0.003361	0.02413	0.01878472	Glra2/Glra1	2
MF	GO:0022849	glutamate-gated calcium ion channel act	2/373	5/19943	0.003361	0.02413	0.01878472	Grin1/Grin2b	2
MF	GO:0098973	structural constituent of postsynaptic ac	2/373	5/19943	0.003361	0.02413	0.01878472	Ina/Camk2b	2
MF	GO:1901981	phosphatidylinositol phosphate binding	9/373	162/19943	0.003507	0.024973	0.01944111	Rph3a/Cadps/Kcnh1/Clvs1/Pfn2/LOC100909840/Amer3/Syt7/Hcn	9
MF	GO:0030507	spectrin binding	4/373	34/19943	0.003585	0.025388	0.0197643	Dync1i1/Dmtn/Ank1/Add2	4
MF	GO:0043492	ATPase activity, coupled to movement o	8/373	133/19943	0.003589	0.025388	0.0197643	Atp1a3/Atp8b3/Atp2b2/Atp2b3/Atp8a2/Abca5/Abcg4/Abcc2	8
MF	GO:0003779	actin binding	16/373	406/19943	0.004312	0.029548	0.02300242	Hpca/Syn1/Dmtn/Epb41l3/Spire2/Cap2/Kcnma1/Coro2a/Map2/Pı	16
MF	GO:0042277	peptide binding	13/373	302/19943	0.004713	0.031942	0.02486603	Cabp1/Oprd1/Pex5l/Hcrtr2/Grin1/Hcrtr1/Dlgap3/Crhbp/Atp1a3/D	13
MF	GO:0031802	type 5 metabotropic glutamate receptor	2/373	6/19943	0.004979	0.032778	0.0255171	Necab2/Dnm3	2
MF	GO:0033265	choline binding	2/373	6/19943	0.004979	0.032778	0.0255171	Slc5a7/Chat	2
MF	GO:0099507	ligand-gated ion channel activity involve	2/373	6/19943	0.004979	0.032778	0.0255171	Grin1/Kcnma1	2
MF	GO:0031489	myosin V binding	3/373	19/19943	0.005034	0.032843	0.02556785	Rab6b/Rab3c/Rab3a	3
MF	GO:0005242	inward rectifier potassium channel activi	3/373	20/19943	0.005841	0.037001	0.02880497	Kcnj3/Kcnj12/Kcnh7	3
MF	GO:0005540	hyaluronic acid binding	3/373	21/19943	0.006722	0.041964	0.03266815	Acan/Hapln1/Hapln4	3
MF	GO:0004972	NMDA glutamate receptor activity	2/373	7/19943	0.006885	0.041964	0.03266815	Grin1/Grin2b	2
MF	GO:0004985	opioid receptor activity	2/373	7/19943	0.006885	0.041964	0.03266815	Oprd1/Oprl1	2
MF	GO:0016907	G protein-coupled acetylcholine recepto	2/373	7/19943	0.006885	0.041964	0.03266815	Chrm2/Hrh3	2
MF	GO:0019992	diacylglycerol binding	2/373	7/19943	0.006885	0.041964	0.03266815	Rasgrp1/Unc13c	2

Activated genes_KEGG

ID	Description	GeneRatio	BgRatio	pvalue	p.adjust	qvalue	geneID	Count
rno04080	Neuroactive ligand-receptor interaction	27/98	284/6610	2.41849E-15	2.51523E-13	2.01116E-13	Grm4/Gabrg2/Glra2/Gabra1/Trhr/Drd2/Oprd1/	27
rno04020	Calcium signaling pathway	20/98	186/6610	1.76863E-12	9.1969E-11	7.35379E-11	Trhr/Grin1/Grm1/Ryr2/Atp2b2/Htr2c/Chrm2/C	20
rno04720	Long-term potentiation	8/98	69/6610	6.95357E-06	0.000241057	0.000192748	Rps6ka6/Grin1/Grm1/Camk2b/Camk2a/Adcy1/	8
rno04540	Gap junction	8/98	89/6610	4.60497E-05	0.001197292	0.000957349	Gjd2/Drd2/Grm1/Htr2c/Tuba8/Gnas/Adcy1/Htı	8
rno04972	Pancreatic secretion	8/98	104/6610	0.000139958	0.002632948	0.002105292	Ryr2/Atp1a3/Pla2g4e/Atp2b2/Atp2b3/Gnas/Ac	8
rno04010	MAPK signaling pathway	13/98	269/6610	0.000151901	0.002632948	0.002105292	Fgf12/Rps6ka6/Cacng2/Cacng3/Rasgrf1/Pla2g4	13
rno05414	Dilated cardiomyopathy	7/98	88/6610	0.000302343	0.004491952	0.003591743	Cacng2/Cacng3/Ryr2/Gnas/Adcy1/Cacna2d2/Ci	7
rno04970	Salivary secretion	6/98	78/6610	0.000989171	0.012859224	0.010282173	Atp1a3/Atp2b2/Atp2b3/Gnas/Adcy1/Kcnma1	6
rno04260	Cardiac muscle contraction	6/98	84/6610	0.001457718	0.016844736	0.013468969	Cacng2/Cacng3/Ryr2/Atp1a3/Cacna2d2/Cacna:	6
rno05014	Amyotrophic lateral sclerosis (ALS)	5/98	66/6610	0.002827798	0.029409096	0.02351537	Nefh/Grin1/Nefl/Nefm/Grin2b	5
rno04912	GnRH signaling pathway	6/98	99/6610	0.003363685	0.031802111	0.025428813	Pla2g4e/Camk2b/Camk2a/Gnas/Adcy1/Mapk1(	6
rno05412	Arrhythmogenic right ventricular cardiomyopathy	5/98	72/6610	0.00412902	0.035784842	0.028613386	Cacng2/Cacng3/Ryr2/Cacna2d2/Cacna2d1	5
rno04971	Gastric acid secretion	5/98	74/6610	0.004644749	0.037157991	0.029711349	Atp1a3/Camk2b/Camk2a/Gnas/Adcy1	5
rno05410	Hypertrophic cardiomyopathy (HCM)	5/98	80/6610	0.006465856	0.048032075	0.038406214	Cacng2/Cacng3/Ryr2/Cacna2d2/Cacna2d1	5

Table S2. Representative GO terms containing GABAARs in hUC-MSC-activated genes.

GO term	p.adjust	gene
ion channel complex	1.69×10^{-30}	*Gabrg2/Glra2/Gabra1/Scn8a/Kcnj3/Cacng2/Gabra5/Chrna4/Pex5l/Kcns2/Kcnab2/Grin1/Cacng3/Glra1/Dlgap3/Ryr2/Olfm3/Kcna2/Kcnc3/Scn4b/Dpp6/Kcnip4/Nrn1/Scn1a/Kcnc1/Gabra3/Kcnab1/Kcna1/Dpp10/Kcnh1/Cacna1b/Akap6/Scn2a/Kcnma1/Scn9a/Grin2b/Chrna7/Kcnq2/Lrrc7/Cacna2d2/Shank1/Chrnb2/Dlg2/Trpc5/Vwc2l/Cacna2d1/Kcng1/Hcn1/Cacna1i/Gabrb3*
GABA-ergic synapse	6.68×10^{-19}	*Gabrg2/Gabra1/Lrfn5/Drd2/Slitrk3/Gabra5/Rims1/Clstn3/Cntnap4/Slc32a1/Acan/Atp2b2/Gabra3/Atp2b3/Slc6a11/Rims2/Cntn5/Gabbr2/Nrg1/Erc2/Camk4/Syt7/Bsn/Nrxn3/Gabrb3*
synaptic transmission, GABAergic	9.40×10^{-10}	*Phf24/Grm4/Gabrg2/Gabra1/Drd2/Gabra5/Clstn3/Cntnap4/Car7/Grm1/Gabra3/Prkce/Gabrb3*
GABA receptor complex	3.29×10^{-5}	*Gabrg2/Gabra1/Gabra5/Gabra3/Gabbr2/Gabrb3*
GABA-gated chloride ion channel activity	4.58×10^{-5}	*Gabrg2/Gabra1/Gabra5/Gabra3/Gabrb3*
GABA-A receptor complex	3.20×10^{-4}	*Gabrg2/Gabra1/Gabra5/Gabra3/Gabrb3*
chloride transport	3.53×10^{-4}	*Gabrg2/Glra2/Gabra1/Gabra5/Car7/Glra1/Slc12a5/Gabra3/Slc12a3/Gabrb3*
chloride transmembrane transporter activity	1.09×10^{-3}	*Gabrg2/Glra2/Gabra1/Gabra5/Glra1/Slc12a5/Gabra3/Slc12a3/Gabrb3*

Table S3. Antibodies.

antibody	company	cat#	Application
Purified anti-GABRA1 Antibody (m)	Biolegend	832301	WB, IF
Anti-GABA A Receptor alpha 3/GABRA3 antibody (R)	Abcam	ab72446	WB, IF
Anti-GABA A Receptor alpha 5/GABRA5 antibody (R)	Abcam	ab10098	WB, IF
Anti-GABA A Receptor beta 3/GABRB3 antibody [N87/25] (m)	Abcam	ab98968	WB, IF
GABRG2 Antibody (R)	Proteintech	14104-1-AP	WB, IF
KCC2 Monoclonal Antibody (S1-12) (m)	Invitrogen	MA5-27610	WB, IF
Anti-K+/Cl- Cotransporter (KCC2) Antibody (R)	millpore	07-432	WB, IF
Monoclonal Anti-HA, clone HA-7, produced in mouse, ascites fluid (m)	sigma	H9658-200UL	WB, IF
HA tag Rabbit Polyclonal Antibody (R)	Proteintech	51064-2-AP-100ul	WB, IF
Anti-FLAG® Antibody produced in rabbit (R)	Sigma	F7425-.2MG	WB
Flag tag Antibody (m)	Proteintech	20543-1-AP	IF
Anti-NeuN Antibody, clone A60 (m)	millpore	MAB377	WB, IF
Recombinant Anti-NeuN antibody [EPR12763] - Neuronal Marker (R)	Abcam	ab177487-40ul	WB, IF
Anti-MAP2 antibody (chicken)	Abcam	ab5392	WB, IF
BDNF Monoclonal Antibody (m)	Proteintech	66292-1-Ig	WB
GAPDH Mouse Monoclonal Antibody	Beijing Ray antibody Biotech	RM2002	WB
Goat anti-Rabbit IgG (H+L) Cross-Adsorbed Secondary Antibody, Alexa Fluor 488	Thermo	A-11008	IF
Goat anti-Mouse IgG (H+L) Highly Cross-Adsorbed Secondary Antibody, Alexa Fluor Plus 488	Thermo	A32723	IF
Donkey anti-Rabbit IgG (H+L) Cross-Adsorbed Secondary Antibody, Alexa Fluor 594	Thermo	A21207	IF
Goat anti-Mouse IgG (H+L) Cross-Adsorbed Secondary Antibody, Alexa Fluor 594	Thermo	A-11005	IF
Goat anti-Chicken IgG (H+L) Cross-Adsorbed Secondary Antibody, Alexa Fluor 568	Thermo	A11041	IF
IGG(H+L)Peroxidase AffiniPure Goat Anti-Rabbit	Jackson	111-035-003	WB
IGG(H+L)Peroxidase AffiniPure Goat Anti-Mouse	Jackson	115-035-003	WB
CD105 (Endoglin) Monoclonal Antibody (SN6), PE, eBioscience™	invitrogen	12-1057-41	Flow
CD90 (Thy-1) Monoclonal Antibody (eBio5E10 (5E10)), PE-Cyanine7, eBioscience™	invitrogen	25-0909-41	Flow
CD166 (ALCAM) Monoclonal Antibody (3A6), PE, eBioscience™	invitrogen	12-1668-41	Flow
CD11b Monoclonal Antibody (ICRF44), PE-Cyanine5, eBioscience™	invitrogen	15-0118-41	Flow
CD45 Monoclonal Antibody (HI30), APC-eFluor 780, eBioscience™	invitrogen	47-0459-41	Flow
CD73 Monoclonal Antibody (AD2), APC, eBioscience™	Invitrogen	Cat # 17-0739-41	Flow
HLA-DR Monoclonal Antibody (LN3), FITC, eBioscience™	Invitrogen	Cat # 11-9956-41	Flow
CD34 Monoclonal Antibody (4H11), APC, eBioscience™	Invitrogen	Cat # 17-0349-41	Flow

Table S4. Primers.

Table S3. Primers

Primer Name	Sequence
Fwd_Gabrg2(r)_RT01	CTCCAGTTATGGTTATCCT
Rev_Gabrg2(r)_RT01	TCCATGACCTTGTGTCTC
Fwd_Gabra5(r)_RT01	TGCTTTTACAACTGGGAAG
Rev_Gabra5(r)_RT01	ATTGAGGCTGTACCCACA
Fwd_Gabrb3(r)_RT01	TTCTCCATTGTGGAGCAC
Rev_Gabrb3(r)_RT01	CCGAAAACTCAATGAGAG
Fwd_Slc12a5(r)_RT01	GGAGACAGACCCAGAGAAGG
Rev_Slc12a5(r)_RT01	TTGATCCCCTCCGAGGAGA
Fwd_Gabra1(r)_RT01	ACCATAGAACCGAAAGAA
Rev_Gabra1(r)_RT01	CGCTGTTAAAGGTTTTCT
Fwd_Gabra3(r)_RT01	ACTGTCATTCTGTCACAAGT
Rev_Gabra3(r)_RT01	TGACACCAAAGACTGTGC
Fwd_Gabra4(r)_RT01	ATCAGTGCTCGGCATTCT
Rev_Gabra4(r)_RT01	TATGAACCAATCCATGGC
Fwd_Gabrb1(r)_RT01	AGCAAACAAGACCAGAGT
Rev_Gabrb1(r)_RT01	AACCTGGACTTTGTTCAT
Fwd_Gabrb2(r)_RT01	TGTAAAAGCCATTGACATG
Rev_Gabrb2(r)_RT01	AAGAAGATGTAGTTGACCAA
Fwd_Gabrg3(r)_RT01	TAACCATGACCACACTCAGC
Rev_Gabrg3(r)_RT01	GTGACATAGGACACCCGA
Fwd_Gabrd(R)_RT01	GCGCCAGAGCAATGAATGAC
Rev_Gabrd(R)_RT01	GCGTAGCCCTCCATTAGTCC
Fwd_Gad1(R)_RT01	GATGGTTTTCGATGGT
Rev_Gad1(R)_RT01	CCATGGTTGTTCCTGA
Fwd_Gabbr1(R)_RT01	CTCTCGGGCTGGATGGTTAC
Rev-Gabbr1(R)_RT01	GGCTCTAGGGTCTTCCTCCA
Fwd_Gabbr2(R)_RT01	CAGGGAAGACTCCACAGCAG
Rev-Gabbr2(R)_RT01	AGGCGTACCCATGGAACTTG
Fwd_Gabra2(R)_RT01	CGTATGGTTTCCGCTGCTTG
Rev_Gabra2(R)_RT01	GTAATGCTGTCTCCCAGTCCT
Fwd_Gad2(R)_RT02	AATTATGCACTTCTCCACGCAACA
Rev_Gad2(R)_RT02	GAAATGCGAGAGTGGGCCTTT
Fwd_Gabrg1(R)_RT03	TCTGGTTTACTGGGTTGG
Rev_Gabrg1(R)_RT03	CGAAGTGATTATATTGGACT
Fwd_slc6a1(r)_RT01	TGTGTAGGCAATCTACCAGC
Rev_slc6a1(r)_RT01	AGCAGGTAGATGCCTCAG
Fwd_Slc12a2(r)_RT02	CTCTGTCATCCTAAGTGGAC
Rev_Slc12a2(r)_RT02	CGAAACACACAGCTCTCA
Fwd_Slc32a1(r)_RT01	TGCCCAGCCTCTTCCACTT
Rev_Slc32a1(r)_RT01	AGATGCCGCCGATGACGAA
Fwd_28srRNA(m)_RT01	GTGACGCGCATGAATGGA
Rev_28srRNA(m)_RT01	TGTGGTTTCGCTGGATAGTAGGT
dsDNA: 3XFlag-MCS-3XHA	CAATTGGCCACCATGGACTACAAGGACCATGACGGAGACTACAAGGACCATGACATCGACTACAAGGACGACGATGACAAGGAATTCCTC<br>GAGACTAGTTCTAGAGCGGCCGCGGATCCGTACCCATACGATGTTCCTGACTATGCGGGCTATCCCTATGACGTCCCGGACTATGCAGGAT<br>CGTATCCTTATGACGTTCCAGATTACGCTGGGTCCTAAAGATCT
Flag-HA F	GAGCGCAGTCGAGAAGGTACCCAATTGTGGCCACCATGGACTACAAGGACCATGACG
Flag-HA R	AGAGGTTGATTATCGATAAGCTTTTAGGACCCAGCGTAATCTGGAA
Gabra1 F	GGACGACGATGACAAGGAATTCatgaagaaaagtcggggtct
Gabra1 R	AACATCGTATGGGTACGGATCttgatggggtgtgggggcttttag
Gabra3 F	GGACGACGATGACAAGGAATTCatgataaccacacaaatgtg
Gabra3 R	AACATCGTATGGGTACGGATCctgtttgcggatcatgccct
rGABRA5-F	GGACGACGATGACAAGGAATTCatggacaatggaatgctctctaga
rGABRA5-R	GAACATCGTATGGGTACGGATCctttggagaggtagcccct
rKCC2-F	GGACGACGATGACAAGGAATTCatgctcaacaacctgacggact
rKCC2-R	GAACATCGTATGGGTACGGATCggagtagatggtgatgacct
Fwd_Gabrb3(R)_EcoRI_one step cloning02	GACGACGATGACAAGGAATTCATGTGGGGCTTTGCGGGAGG
Rev_Gabrb3 (R)_BamHI_one step cloning	ACATCGTATGGGTACGGATCCCAGTTAACATAGTACAGCCAG
Fwd_Gabrg2(R)_EcoRI_one step cloning02	GACGACGATGACAAGGAATTCATGTGGGGCTTTGCGGGAGG
Rev_Gabrg2(R)_BamHI_one step cloning	ACATCGTATGGGTACGGATCCCACAGATAAAGATAGGAGAC
Fwd_Kpn1_3XFLAG-2	GAGCGCAGTCGAGAAGGTACCGAATTCGGATCCCCATGGGACTACAAGGACCATGACGG
Rev_HindIII_3XFLAG	AGGTTGATTATCGATAAGCTTtcaCTTGTCATCGTCGTCCTTGT
Fwd_Kpn1_3XHA	GAGCGCAGTCGAGAAGGTACCCCATGGGAATTCCTCGAGACTAGTTC
Fwd-c-3XFLAG-KCC2(r)-KpnI	GAGCGCAGTCGAGAAGGTACCatgctcaacaacctgacggact
Rev-c-3XFLAG-KCC2(r)-BamHI	CTTGTAGTCCCATGGGGATCCggagtagatggtgatgacctc
Rev-c-3XHA-KCC2(r)-EcoRI	AGAACTAGTCTCGAGGAATTCggagtagatggtgatgacctc
Fwd_Bdnf(r)-EcoRI_one step cloning	GACGACGATGACAAGGAATTCatgtctgaccccagtgcctg
Rev_Bdnf(r)-NotI_one step cloning	AGAGGGGCGGGATCCGCGGCCGCctatcttccccttttaatgg
